# Supplementary material for: Predicted Reversal in N-Methylazepine/N-Methyl-7-azanorcaradiene Equilibrium upon Formation of Their N-Oxides
Source: Molecules. 2020 Oct 16;25(20):4767. doi: 10.3390/molecules25204767 (PMC7594072; doi:10.3390/molecules25204767)
Supplement: Supplementary file 1 [file molecules-25-04767-s001.zip › Supplemental/Supplemental Information.docx]

***Supporting Information***

**Predicted Reversal in N-Methylazepine/N-Methyl-7-azanorcaradiene Equilibrium Upon Formation of Their N-Oxides**

René Fournier^1^, Alexa R. Green^2^, Arthur Greenberg^2,*^, Edward Lee-Ruff^1,*^, Joel F. Liebman^3,*^ and Anita Rágyanszki^4,*^

1. Department of Chemistry, York University, Keele Campus, Toronto ON MJ3 1P3 Canada
2. Department of Chemistry, University of New Hampshire, Durham, NH 03824 USA
3. Department of Chemistry and Biochemistry, University of Maryland Baltimore County, Baltimore, MD 21250 USA
4. Department of Physics and Astronomy, York University, Keele Campus, Toronto ON MJ3 1P3 Canada

*E-mail: [art.greenberg@unh.edu](mailto:art.greenberg@unh.edu)

[leeruff@yorku.ca](mailto:leeruff@yorku.ca)

[jliebman@umbc.edu](mailto:jliebman@umbc.edu)

[a.ragyanszki@utoronto.ca](mailto:a.ragyanszki@utoronto.ca)

**Cartesian Coordinates (Å) and energies (Hartrees) for all structures calculated using Gaussian 09.**

**N-methylpyrrole**

The number of imaginary frequencies: 0

Total Energy (B3LYP/6-31G*)= -249.47784543

ZPE= -249.367168

Enthalpy= -249.360642

Gibb’s Free Energy= -249.396193

C -0.31737 -1.01923 0.17362

C 1.00946 -1.0133 -0.07679

C 1.4598 0.44188 -0.08284

C 0.36886 1.19813 0.16441

N -0.76109 0.33469 0.58538

H -0.95522 -1.87361 0.08373

H 1.62623 -1.86947 -0.25416

H 2.45195 0.79863 -0.26524

H 0.32489 2.26274 0.06655

C -2.04157 0.72846 -0.0198

H -2.29179 1.72172 0.28958

H -1.95658 0.69773 -1.08597

H -2.80837 0.05254 0.29651

**N-methylpyrrole**

The number of imaginary frequencies: 0

Total Energy (B3LYP/6-31G**)= -249.48868841

ZPE= -249.37822

Enthalpy= -249.371698

Gibb’s Free Energy= -249.407248

C 0. -0.17376 1.09496

C 0. -1.46657 0.6966

C 0. -1.46657 -0.6966

C 0. -0.17376 -1.09496

N 0. 0.60624 0.

H 0. 0.16939 2.10844

H 0. -2.32739 1.33212

H 0. -2.32739 -1.33212

H 0. 0.16939 -2.10844

C 0. 2.07624 0.

H 0.5044 2.43291 -0.87365

H -1.00881 2.43291 0.

H 0.5044 2.43291 0.87365

**N-methylpyrrole**

The number of imaginary frequencies: 0

Total Energy (M06/6-311G+**)= -249.34878244

ZPE= -249.239616

Enthalpy= -249.232966

Gibb’s Free Energy= -249.269338

C -0.31737 -1.01923 0.17362

C 1.00946 -1.0133 -0.07679

C 1.4598 0.44188 -0.08284

C 0.36886 1.19813 0.16441

N -0.76109 0.33469 0.58538

H -0.95522 -1.87361 0.08373

H 1.62623 -1.86947 -0.25416

H 2.45195 0.79863 -0.26524

H 0.32489 2.26274 0.06655

C -2.04157 0.72846 -0.0198

H -2.29179 1.72172 0.28958

H -1.95658 0.69773 -1.08597

H -2.80837 0.05254 0.29651

**N-Methylpyrrole N-oxide**

The number of imaginary frequencies: 0

Total Energy (B3LYP/6-31G*)= -324.56298019

ZPE= -324.449454

Enthalpy= -324.442285

Gibb’s Free Energy= -324.479007

C -0.3294 -1.01791 0.1578

C 1.00468 -1.01444 -0.06029

C 1.45655 0.44566 -0.06634

C 0.35814 1.20372 0.14859

N -0.75911 0.33403 0.57648

H -0.97374 -1.86404 0.04035

H 1.62512 -1.87187 -0.21771

H 2.45247 0.80154 -0.22879

H 0.30409 2.26497 0.02323

C -2.04454 0.72939 -0.01703

H -2.29215 1.72185 0.29697

H -1.96841 0.7014 -1.08395

H -2.80872 0.05267 0.30389

O -0.87602 0.37583 1.93081

**N-Methylpyrrole N-oxide**

The number of imaginary frequencies: 0

Total Energy (B3LYP/6-31G**)= -324.57360076

ZPE= -324.46035

Enthalpy= -324.453172

Gibb’s Free Energy= -324.489906

C -0.04298 0.35255 1.15778

C -0.04298 1.62083 0.7378

C -0.04298 1.62083 -0.7378

C -0.04298 0.35255 -1.15778

N -0.04755 -0.56912 0.

H -0.10099 -0.10602 2.13324

H -0.09948 2.50313 1.36302

H -0.09948 2.50313 -1.36302

H -0.10099 -0.10602 -2.13324

C 1.16596 -1.45463 0.

H 1.08767 -2.08133 -0.8887

H 2.08944 -0.86521 0.

H 1.08767 -2.08133 0.8887

O -1.18692 -1.34192 0.

**N-methylpyrrole N-oxide**

The number of imaginary frequencies: 0

Total Energy (M06/6-311G+**)= -324.43163272

ZPE= -324.31929

Enthalpy= -324.312193

Gibb’s Free Energy= -324.348742

C -0.3294 -1.01791 0.1578

C 1.00468 -1.01444 -0.06029

C 1.45655 0.44566 -0.06634

C 0.35814 1.20372 0.14859

N -0.75911 0.33403 0.57648

H -0.97374 -1.86404 0.04035

H 1.62512 -1.87187 -0.21771

H 2.45247 0.80154 -0.22879

H 0.30409 2.26497 0.02323

C -2.04454 0.72939 -0.01703

H -2.29215 1.72185 0.29697

H -1.96841 0.7014 -1.08395

H -2.80872 0.05267 0.30389

O -0.87602 0.37583 1.93081

**N-methylazepine**

The number of imaginary frequencies: 0

Total Energy (B3LYP/6-31G*)= -326.84608435

ZPE= -326.702253

Enthalpy= -326.693875

Gibb’s Free Energy= -326.733659

C 0.93863 0.15438 -0.2526

C 2.30347 0.15457 -0.25898

C 3.19274 1.20123 0.04876

C 0.05199 1.2008 0.06344

C 2.91009 2.40177 0.60544

C 0.33949 2.40141 0.61745

H 0.47792 -0.76674 -0.54277

H 2.7617 -0.76642 -0.55344

H 4.22198 1.0244 -0.18427

H -0.97933 1.02368 -0.15996

H 3.70896 3.11213 0.6512

H -0.45911 3.11155 0.67068

N 1.62734 2.81624 1.17006

C 1.63404 2.44107 2.59136

H 1.63673 3.32544 3.19368

H 0.76149 1.86222 2.81157

H 2.50877 1.86246 2.8034

**N-methylazepine**

The number of imaginary frequencies: 0

Total Energy (B3LYP/6-31G**)= -326.85965616

ZPE= -326.716154

Enthalpy= -326.70778

Gibb’s Free Energy= -326.747543

C 0.61021 1.77096 0.67637

C 0.61021 1.77096 -0.67637

C 0.61021 0.57868 -1.51904

C 0.61021 0.57868 1.51904

C 0.05418 -0.6014 -1.19368

C 0.05418 -0.6014 1.19368

H 0.69105 2.72773 1.19091

H 0.69105 2.72773 -1.19091

H 1.12518 0.64113 -2.47565

H 1.12518 0.64113 2.47565

H 0.1529 -1.46083 -1.86048

H 0.1529 -1.46083 1.86048

N -0.68116 -0.8274 0.

C -1.52746 -2.0092 0.

H -0.95778 -2.95729 0.

H -2.17143 -1.99535 0.88425

H -2.17143 -1.99535 -0.88425

**N-methylazepine**

The number of imaginary frequencies: 0

Total Energy (M06/6-311G+**)= -326.67492642

ZPE= -326.532731

Enthalpy= -326.524386

Gibb’s Free Energy= -326.563984

C 0.62245 1.75336 0.67222

C 0.62245 1.75336 -0.67222

C 0.62245 0.56474 -1.50646

C 0.62245 0.56474 1.50646

C 0.04331 -0.59604 -1.18419

C 0.04331 -0.59604 1.18419

H 0.70572 2.70987 1.18507

H 0.70572 2.70987 -1.18507

H 1.14071 0.61519 -2.46069

H 1.14071 0.61519 2.46069

H 0.11856 -1.45761 -1.85176

H 0.11856 -1.45761 1.85176

N -0.69474 -0.80586 0.

C -1.53524 -1.98153 0.

H -0.95466 -2.92166 0.

H -2.17953 -1.97387 0.88285

H -2.17953 -1.97387 -0.88285

**N-methylazepine N-oxide**

The number of imaginary frequencies: 0

Total Energy (B3LYP/6-31G*)= -401.97429587

ZPE= -401.825754

Enthalpy= -401.817041

Gibb’s Free Energy= -401.857431

C 0.82605 0.55465 -0.65489

C 2.17148 0.54639 -0.76242

C 3.08766 1.44622 -0.20978

C 0.02032 1.46506 0.03537

C 2.88442 2.40908 0.72364

C 0.38096 2.42445 0.92372

H 0.31053 -0.22416 -1.17699

H 2.58798 -0.23815 -1.35901

H 4.09119 1.36074 -0.57104

H -1.02882 1.39218 -0.16184

H 3.7184 3.0554 0.90153

H -0.40623 3.08073 1.23117

N 1.69221 2.67973 1.54823

C 1.78348 1.84441 2.75439

H 2.70185 2.05143 3.26295

H 0.96013 2.06213 3.40215

H 1.75489 0.81166 2.476

O 1.72659 3.99895 1.87699

**N-methylazepine N-oxide**

The number of imaginary frequencies: 0

Total Energy (B3LYP/6-31G**)= -401.98779312

ZPE= -401.839695

Enthalpy= -401.830971

Gibb’s Free Energy= -401.871365

C 0.75349 1.83108 0.68076

C 0.75349 1.83108 -0.68076

C 0.75349 0.64759 -1.50595

C 0.75349 0.64759 1.50595

C 0.29957 -0.58124 -1.1975

C 0.29957 -0.58124 1.1975

H 0.87786 2.78202 1.19381

H 0.87786 2.78202 -1.19381

H 1.21424 0.74192 -2.48817

H 1.21424 0.74192 2.48817

H 0.42486 -1.46195 -1.81406

H 0.42486 -1.46195 1.81406

N -0.50404 -0.94862 0.

C -1.83882 -0.25012 0.

H -2.35394 -0.60559 -0.89226

H -2.35394 -0.60559 0.89226

H -1.73292 0.83728 0.

O -0.7138 -2.29727 0.

**N-methylazepine N-oxide**

The number of imaginary frequencies: 0

Total Energy (M06/6-311G+**)= -401.79814103

ZPE= -401.651151

Enthalpy= -401.642487

Gibb’s Free Energy= -401.682651

C 0.82605 0.55465 -0.65489

C 2.17148 0.54639 -0.76242

C 3.08766 1.44622 -0.20978

C 0.02032 1.46506 0.03537

C 2.88442 2.40908 0.72364

C 0.38096 2.42445 0.92372

H 0.31053 -0.22416 -1.17699

H 2.58798 -0.23815 -1.35901

H 4.09119 1.36074 -0.57104

H -1.02882 1.39218 -0.16184

H 3.7184 3.0554 0.90153

H -0.40623 3.08073 1.23117

N 1.69221 2.67973 1.54823

C 1.78348 1.84441 2.75439

H 2.70185 2.05143 3.26295

H 0.96013 2.06213 3.40215

H 1.75489 0.81166 2.476

O 1.72659 3.99895 1.87699

**1,3-Cyclopentadiene**

The number of imaginary frequencies: 0

Total Energy (B3LYP/6-31G*)= -194.10105812

ZPE= -194.008165

Enthalpy= -194.003078

Gibb’s Free Energy= -194.034762

C -0.03204 0.83323 -0.57221

C 1.30621 0.8271 -0.35761

C 1.75514 2.2525 0.01266

C 0.56916 3.10434 -0.4754

C -0.50584 2.29711 -0.64793

H -0.65144 -0.03154 -0.68801

H 1.94617 -0.02657 -0.43883

H 1.85482 2.33968 1.07444

H 2.68483 2.53007 -0.43847

H 0.5915 4.15886 -0.65532

H -1.51004 2.62121 -0.82523

**1,3-Cyclopentadiene**

The number of imaginary frequencies: 0

Total Energy (B3LYP/6-31G**)= -194.11069027

ZPE= -194.017995

Enthalpy= -194.012901

Gibb’s Free Energy= -194.044595

C -0.03204 0.83323 -0.57221

C 1.30621 0.8271 -0.35761

C 1.75514 2.2525 0.01266

C 0.56916 3.10434 -0.4754

C -0.50584 2.29711 -0.64793

H -0.65144 -0.03154 -0.68801

H 1.94617 -0.02657 -0.43883

H 1.85482 2.33968 1.07444

H 2.68483 2.53007 -0.43847

H 0.5915 4.15886 -0.65532

H -1.51004 2.62121 -0.82523

**1,3-Cyclopentadiene**

The number of imaginary frequencies: 0

Total Energy (M06/6-311G+**)= -193.99039929

ZPE= -193.898575

Enthalpy= -193.893442

Gibb’s Free Energy= -193.925181

C -0.03204 0.83323 -0.57221

C 1.30621 0.8271 -0.35761

C 1.75514 2.2525 0.01266

C 0.56916 3.10434 -0.4754

C -0.50584 2.29711 -0.64793

H -0.65144 -0.03154 -0.68801

H 1.94617 -0.02657 -0.43883

H 1.85482 2.33968 1.07444

H 2.68483 2.53007 -0.43847

H 0.5915 4.15886 -0.65532

H -1.51004 2.62121 -0.82523

**1,3,5-Cycloheptatriene**

The number of imaginary frequencies: 0

Total Energy (B3LYP/6-31G*)= -271.50958752

ZPE=-271.381192

Enthalpy= -271.374505

Gibb’s Free Energy= -271.41033

C -0.20071 -0.81856 0.32651

C 1.06397 -0.49637 -0.04424

C 1.68974 0.89155 -0.31285

C -1.34606 0.10738 0.76151

C 1.19882 2.07325 0.5547

C -1.27725 1.41867 1.06715

C -0.03587 2.32272 1.05869

H 1.5021 1.14001 -1.33655

H -0.43367 -1.86275 0.30957

H 1.721 -1.32797 -0.19136

H 2.74077 0.79537 -0.13682

H -2.31134 -0.34609 0.84812

H 1.9384 2.81469 0.77422

H -2.19448 1.88081 1.36717

H -0.16354 3.28474 1.50936

**1,3,5-Cycloheptatriene**

The number of imaginary frequencies: 0

Total Energy (B3LYP/6-31G**)= -271.52195835

ZPE=-271.393853

Enthalpy= -271.387159

Gibb’s Free Energy= -271.422993

C -0.20071 -0.81856 0.32651

C 1.06397 -0.49637 -0.04424

C 1.68974 0.89155 -0.31285

C -1.34606 0.10738 0.76151

C 1.19882 2.07325 0.5547

C -1.27725 1.41867 1.06715

C -0.03587 2.32272 1.05869

H 1.5021 1.14001 -1.33655

H -0.43367 -1.86275 0.30957

H 1.721 -1.32797 -0.19136

H 2.74077 0.79537 -0.13682

H -2.31134 -0.34609 0.84812

H 1.9384 2.81469 0.77422

H -2.19448 1.88081 1.36717

H -0.16354 3.28474 1.50936

**1,3,5-Cycloheptatriene**

The number of imaginary frequencies: 0

Total Energy (M06/6-311G+**)= -271.35461618

ZPE=-271.227611

Enthalpy= -271.22088

Gibb’s Free Energy=-271.256734

C -0.20071 -0.81856 0.32651

C 1.06397 -0.49637 -0.04424

C 1.68974 0.89155 -0.31285

C -1.34606 0.10738 0.76151

C 1.19882 2.07325 0.5547

C -1.27725 1.41867 1.06715

C -0.03587 2.32272 1.05869

H 1.5021 1.14001 -1.33655

H -0.43367 -1.86275 0.30957

H 1.721 -1.32797 -0.19136

H 2.74077 0.79537 -0.13682

H -2.31134 -0.34609 0.84812

H 1.9384 2.81469 0.77422

H -2.19448 1.88081 1.36717

H -0.16354 3.28474 1.50936

**2,4-Cyclopentadienone**

The number of imaginary frequencies: 0

Total Energy (B3LYP/6-31G*)= -268.10366059

ZPE=-268.029249

Enthalpy= -268.023604

Gibb’s Free Energy= -268.057109

C -0.01597 0.56911 -0.63699

C 1.53525 0.57813 -0.59521

C 1.96549 1.79004 -0.15552

C 0.72782 2.6481 0.11742

C -0.48331 1.77579 -0.22148

H -0.62226 -0.25716 -0.94455

H 2.16679 -0.24094 -0.86942

H 2.9842 2.08978 -0.02407

H -1.51104 2.06363 -0.14516

O 0.70962 3.83439 0.53688

**2,4-Cyclopentadienone**

The number of imaginary frequencies: 0

Total Energy (B3LYP/6-31G**)= -268.11018199

ZPE=-268.035857

Enthalpy= -268.030211

Gibb’s Free Energy= -268.063716

C -0.01597 0.56911 -0.63699

C 1.53525 0.57813 -0.59521

C 1.96549 1.79004 -0.15552

C 0.72782 2.6481 0.11742

C -0.48331 1.77579 -0.22148

H -0.62226 -0.25716 -0.94455

H 2.16679 -0.24094 -0.86942

H 2.9842 2.08978 -0.02407

H -1.51104 2.06363 -0.14516

O 0.70962 3.83439 0.53688

**2,4-Cyclopentadienone**

The number of imaginary frequencies: 0

Total Energy (M06/6-311G+**)=-267.98852408

ZPE=-267.914874

Enthalpy= -267.909179

Gibb’s Free Energy= -267.942763

C -0.01597 0.56911 -0.63699

C 1.53525 0.57813 -0.59521

C 1.96549 1.79004 -0.15552

C 0.72782 2.6481 0.11742

C -0.48331 1.77579 -0.22148

H -0.62226 -0.25716 -0.94455

H 2.16679 -0.24094 -0.86942

H 2.9842 2.08978 -0.02407

H -1.51104 2.06363 -0.14516

O 0.70962 3.83439 0.53688

**2,4,6-Cycloheptatrienone**

The number of imaginary frequencies: 0

Total Energy (B3LYP/6-31G*)= -345.53739483

ZPE= -345.426757

Enthalpy= -345.41951

Gibb’s Free Energy= -345.457457

C 0.4044 -0.40001 -0.15775

C 1.77241 -0.40055 -0.15529

C 2.69105 0.61692 0.1737

C -0.51461 0.61819 0.16794

C 2.45079 1.88455 0.58091

C -0.2748 1.88563 0.576

C 1.08787 2.54419 0.79042

H -0.04191 -1.32554 -0.45626

H 2.21906 -1.32643 -0.4522

H 3.72353 0.34896 0.08952

H -1.54699 0.35106 0.08004

H 3.3092 2.49215 0.77797

H -1.13344 2.49392 0.76997

O 1.08765 3.74217 1.17569

**2,4,6-Cycloheptatrienone**

The number of imaginary frequencies: 0

Total Energy (B3LYP/6-31G**)= -345.54656227

ZPE=-345.436187

Enthalpy=-345.428926

Gibb’s Free Energy= -345.466909

C 0.4044 -0.40001 -0.15775

C 1.77241 -0.40055 -0.15529

C 2.69105 0.61692 0.1737

C -0.51461 0.61819 0.16794

C 2.45079 1.88455 0.58091

C -0.2748 1.88563 0.576

C 1.08787 2.54419 0.79042

H -0.04191 -1.32554 -0.45626

H 2.21906 -1.32643 -0.4522

H 3.72353 0.34896 0.08952

H -1.54699 0.35106 0.08004

H 3.3092 2.49215 0.77797

H -1.13344 2.49392 0.76997

O 1.08765 3.74217 1.17569

**2,4,6-Cycloheptatrienone**

The number of imaginary frequencies: 0

Total Energy (M06/6-311G+**)= -345.37465529

ZPE=-345.265377

Enthalpy=-345.257928

Gibb’s Free Energy= -345.297053

C 0.4044 -0.40001 -0.15775

C 1.77241 -0.40055 -0.15529

C 2.69105 0.61692 0.1737

C -0.51461 0.61819 0.16794

C 2.45079 1.88455 0.58091

C -0.2748 1.88563 0.576

C 1.08787 2.54419 0.79042

H -0.04191 -1.32554 -0.45626

H 2.21906 -1.32643 -0.4522

H 3.72353 0.34896 0.08952

H -1.54699 0.35106 0.08004

H 3.3092 2.49215 0.77797

H -1.13344 2.49392 0.76997

O 1.08765 3.74217 1.17569

**2,3-Epoxide N-Methylazepine**

The number of imaginary frequencies: 0

Total Energy (B3LYP/6-31G*)= -402.0126142

ZPE= -401.86298

Enthalpy= -401.854323

Gibb’s Free Energy= -401.894916

C -1.97153 -0.86665 0.21864

C -2.0792 0.477 0.09836

C -0.81657 1.03296 -0.45425

C -0.76447 -1.68529 -0.03687

C 0.35442 0.76433 0.37226

C 0.56633 -1.4101 -0.22187

H -2.85437 -1.44535 0.48705

H -2.99559 1.0283 0.28163

H -0.97814 -2.75026 -0.09528

H 1.23067 -2.25728 -0.38004

N 1.2452 -0.18133 -0.21943

C 2.65217 -0.16427 0.16546

H 2.80272 -0.32861 1.24518

H 3.19049 -0.94245 -0.38388

H 3.08022 0.8047 -0.10428

O 0.07826 2.101 -0.03692

H 0.21628 0.62946 1.44876

H -0.68167 0.83496 -1.51815

**2,3-Epoxide N-Methylazepine**

The number of imaginary frequencies: 0

Total Energy (B3LYP/6-31G**)= -402.02568814

ZPE= -401.876485

Enthalpy= -401.867821

Gibb’s Free Energy= -401.908422

C 0.98337 1.56503 0.95991

C 1.51419 1.48097 -0.29992

C 0.77098 0.59913 -1.22425

C -0.27211 0.73245 1.52549

C 0.33821 -0.65376 -0.82677

C -1.08316 -0.29294 0.95543

H 1.45087 2.2507 1.63534

H 2.39553 2.01271 -0.59215

H -0.54747 0.99384 2.52587

H -1.84555 -0.69634 1.58863

N -0.9916 -0.87598 -0.41373

C -1.31788 -2.30927 -0.40307

H -0.64737 -2.82283 0.25387

H -2.32339 -2.44364 -0.06277

H -1.22039 -2.70462 -1.39256

O 1.10315 -0.57651 -2.16386

H 0.91807 -1.18081 -0.09815

H 0.06457 1.25633 -1.68681

**2,3-Epoxide N-Methylazepine**

The number of imaginary frequencies: 0

Total Energy (M06/6-311G+**)= -401.83976324

ZPE= -401.691752

Enthalpy= -401.683102

Gibb’s Free Energy= -401.72367

C -1.97153 -0.86665 0.21864

C -2.0792 0.477 0.09836

C -0.81657 1.03296 -0.45425

C -0.76447 -1.68529 -0.03687

C 0.35442 0.76433 0.37226

C 0.56633 -1.4101 -0.22187

H -2.85437 -1.44535 0.48705

H -2.99559 1.0283 0.28163

H -0.97814 -2.75026 -0.09528

H 1.23067 -2.25728 -0.38004

N 1.2452 -0.18133 -0.21943

C 2.65217 -0.16427 0.16546

H 2.80272 -0.32861 1.24518

H 3.19049 -0.94245 -0.38388

H 3.08022 0.8047 -0.10428

O 0.07826 2.101 -0.03692

H 0.21628 0.62946 1.44876

H -0.68167 0.83496 -1.51815

**4,5-Epoxide N-Methylazepine**

The number of imaginary frequencies: 0

Total Energy (B3LYP/6-31G*)= -402.04752008

ZPE= -401.897386

Enthalpy= -401.888458

Gibb’s Free Energy= -401.930104

C -1.64868 -0.71215 0.39745

C -1.655 0.71913 0.35793

C -0.44281 1.62606 0.11899

C -0.44434 -1.62081 0.12824

C 0.86032 1.29035 -0.06609

C 0.85605 -1.28843 -0.07968

H -2.26809 -1.18847 1.12845

H -0.65255 2.67515 0.10108

H -0.6576 -2.66917 0.10912

H 1.55506 2.09233 0.07199

H 1.54753 -2.09766 0.02944

N 1.47105 0.0019 -0.46648

C 2.81324 -0.00515 0.133

H 3.34229 -0.87836 -0.18718

H 3.34692 0.86891 -0.177

H 2.72748 -0.01113 1.19955

H -2.23316 1.23268 1.09746

O -2.37974 -0.01144 -0.75584

**4,5-Epoxide N-Methylazepine**

The number of imaginary frequencies: 0

Total Energy (B3LYP/6-31G**)= -402.0607848

ZPE= -401.911089

Enthalpy=-401.902152

Gibb’s Free Energy= -401.943808

C -1.62994 -0.73255 0.39014

C -1.62993 0.73255 0.39016

C -0.4817 1.5723 -0.0053

C -0.48168 -1.57231 -0.00529

C 0.81741 1.23806 -0.12102

C 0.81742 -1.23805 -0.12103

H -2.3009 -1.21971 1.10274

H -0.71282 2.61654 -0.20338

H -0.7128 -2.61655 -0.20337

H 1.52499 2.03453 -0.34095

H 1.52502 -2.03452 -0.34094

N 1.44508 0.00001 -0.0493

C 2.8978 0. 0.11175

H 3.32482 -0.88576 -0.36736

H 3.3248 0.88584 -0.36723

H 3.19608 -0.00007 1.16878

H -2.30097 1.21969 1.1027

O -2.35501 -0.00001 -0.63029

**4,5-Epoxide N-Methylazepine**

The number of imaginary frequencies: 0

Total Energy (M06/6-311G+**)= -401.87335865

ZPE= -401.724678

Enthalpy= -401.715624

Gibb’s Free Energy= -401.757962

C -1.62994 -0.73255 0.39014

C -1.62993 0.73255 0.39016

C -0.4817 1.5723 -0.0053

C -0.48168 -1.57231 -0.00529

C 0.81741 1.23806 -0.12102

C 0.81742 -1.23805 -0.12103

H -2.3009 -1.21971 1.10274

H -0.71282 2.61654 -0.20338

H -0.7128 -2.61655 -0.20337

H 1.52499 2.03453 -0.34095

H 1.52502 -2.03452 -0.34094

N 1.44508 0.00001 -0.0493

C 2.8978 0. 0.11175

H 3.32482 -0.88576 -0.36736

H 3.3248 0.88584 -0.36723

H 3.19608 -0.00007 1.16878

H -2.30097 1.21969 1.1027

O -2.35501 -0.00001 -0.63029

**N-methylpyrrolidine**

The number of imaginary frequencies: 0

Total Energy (B3LYP/6-31G*)= -251.88956837

ZPE= -251.731361

Enthalpy= -251.724164

Gibb’s Free Energy=-251.761212

C -1.15663 -0.45327 0.19297

C 1.15639 -0.45362 0.19329

C 0.77798 1.0303 -0.07053

C -0.77756 1.03066 -0.07012

H -2.05637 -0.7771 -0.33946

H -1.33544 -0.61146 1.26475

H 1.33453 -0.61147 1.26524

H 2.05631 -0.77799 -0.33851

H 1.19788 1.70083 0.6869

H 1.1653 1.36132 -1.04085

H -1.19669 1.70085 0.68805

H -1.16528 1.36262 -1.03996

N -0.00017 -1.27389 -0.20026

C 0.00003 -1.3903 -1.66564

H 0.87482 -1.91947 -1.98127

H -0.87248 -1.92325 -1.98122

H -0.0021 -0.41291 -2.10107

**N-methylpyrrolidine**

The number of imaginary frequencies: 0

Total Energy (B3LYP/6-31G**)= -251.904379

ZPE= -251.746764

Enthalpy= -251.739572

Gibb’s Free Energy=-251.776517

C -0.07138 1.15044 -0.53372

C -0.07129 -1.15029 -0.53401

C -1.2628 -0.77695 0.3945

C -1.26312 0.77669 0.39438

H 0.45507 2.05955 -0.22703

H -0.43402 1.30923 -1.55803

H -0.43417 -1.30864 -1.55831

H 0.45527 -2.0595 -0.22783

H -2.20509 -1.19792 0.02894

H -1.11008 -1.16732 1.4062

H -2.20542 1.19716 0.02825

H -1.11112 1.16732 1.40608

N 0.84465 0.00011 -0.54566

C 1.76509 0. 0.59063

H 2.40898 -0.88422 0.53277

H 2.40905 0.88419 0.53288

H 1.27997 -0.00004 1.58496

**N-methylpyrrolidine**

The number of imaginary frequencies: 0

Total Energy (M06/6-311G+**)= -251.7568498

ZPE= -251.601035

Enthalpy= -251.593775

Gibb’s Free Energy= -251.630876

C -0.07138 1.15044 -0.53372

C -0.07129 -1.15029 -0.53401

C -1.2628 -0.77695 0.3945

C -1.26312 0.77669 0.39438

H 0.45507 2.05955 -0.22703

H -0.43402 1.30923 -1.55803

H -0.43417 -1.30864 -1.55831

H 0.45527 -2.0595 -0.22783

H -2.20509 -1.19792 0.02894

H -1.11008 -1.16732 1.4062

H -2.20542 1.19716 0.02825

H -1.11112 1.16732 1.40608

N 0.84465 0.00011 -0.54566

C 1.76509 0. 0.59063

H 2.40898 -0.88422 0.53277

H 2.40905 0.88419 0.53288

H 1.27997 -0.00004 1.58496

**N-methylpyrrolidine N-oxide**

The number of imaginary frequencies: 0

Total Energy (B3LYP/6-31G*)= -327.03549275

ZPE= -326.872485

Enthalpy= -326.864715

Gibb’s Free Energy= -326.90296

C -1.15663 -0.45327 0.19297

C 1.15639 -0.45362 0.19329

C 0.77798 1.0303 -0.07053

C -0.77756 1.03066 -0.07012

H -2.05637 -0.7771 -0.33946

H -1.33544 -0.61146 1.26475

H 1.33453 -0.61147 1.26524

H 2.05631 -0.77799 -0.33851

H 1.19788 1.70083 0.6869

H 1.1653 1.36132 -1.04085

H -1.19669 1.70085 0.68805

H -1.16528 1.36262 -1.03996

N -0.00017 -1.27389 -0.20026

C 0.00003 -1.3903 -1.66564

H 0.87482 -1.91947 -1.98127

H -0.87248 -1.92325 -1.98122

H -0.0021 -0.41291 -2.10107

O -0.00044 -2.50637 0.37472

**N-methylpyrrolidine N-oxide**

The number of imaginary frequencies: 0

Total Energy (B3LYP/6-31G**)= -327.05036743

ZPE= -326.888069

Enthalpy= -326.880274

Gibb’s Free Energy= -326.918536

C -0.07138 1.15044 -0.53372

C -0.07129 -1.15029 -0.53401

C -1.2628 -0.77695 0.3945

C -1.26312 0.77669 0.39438

H 0.45507 2.05955 -0.22703

H -0.43402 1.30923 -1.55803

H -0.43417 -1.30864 -1.55831

H 0.45527 -2.0595 -0.22783

H -2.20509 -1.19792 0.02894

H -1.11008 -1.16732 1.4062

H -2.20542 1.19716 0.02825

H -1.11112 1.16732 1.40608

N 0.84465 0.00011 -0.54566

C 1.76509 0. 0.59063

H 2.40898 -0.88422 0.53277

H 2.40905 0.88419 0.53288

H 1.27997 -0.00004 1.58496

**N-methylpyrrolidine N-oxide**

The number of imaginary frequencies: 0

Total Energy (M06/6-311G+**)= -326.90051885

ZPE= -326.739215

Enthalpy= -326.731606

Gibb’s Free Energy= -326.769334

C -0.20642 1.18006 -0.39819

C -0.20641 -1.17988 -0.39867

C -1.60313 -0.78221 0.08929

C -1.60323 0.78213 0.08935

H 0.22053 2.074 0.05958

H -0.14015 1.27502 -1.4834

H -0.14032 -1.27427 -1.48394

H 0.22064 -2.07405 0.05855

H -2.37717 -1.19037 -0.56706

H -1.80187 -1.16923 1.09398

H -2.37711 1.1902 -0.56724

H -1.80238 1.16905 1.094

N 0.71326 0.00002 -0.10279

C 1.10744 -0.00026 1.34796

H 1.72001 -0.88912 1.49744

H 1.72009 0.8885 1.49774

H 0.24363 -0.00033 2.02335

O 1.82397 0.00017 -0.86024

**N-methyl-2-pyrroline**

The number of imaginary frequencies: 0

Total Energy (B3LYP/6-31G*)= -250.66822024

ZPE= -250.534319

Enthalpy= -250.527508

Gibb’s Free Energy= -250.563344

C -0.02855 -0.57318 -0.15105

C 1.26736 -0.4801 -0.57951

C 1.79391 0.87643 -0.11602

C 0.48097 1.64558 -0.06848

N -0.40721 0.67078 0.57784

H -0.67354 -1.41071 -0.31672

H 1.80823 -1.21651 -1.13634

H 2.51541 1.32205 -0.76853

H 0.13582 1.83115 -1.06414

H 2.23671 0.78728 0.85396

H 0.54979 2.5825 0.44371

C -1.82735 1.03316 0.46483

H -2.08752 1.13839 -0.56772

H -2.42756 0.26588 0.90747

H -2.00069 1.95899 0.97246

**N-methyl-2-pyrroline**

The number of imaginary frequencies: 0

Total Energy (B3LYP/6-31G**)= -250.68085752

ZPE= -250.547406

Enthalpy= -250.540583

Gibb’s Free Energy= -250.576436

C 0.10596 1.1811 -0.02571

C 1.41809 0.82958 0.13671

C 1.51846 -0.66607 -0.15518

C 0.10274 -1.08391 0.21738

N -0.69193 -0.01873 -0.4077

H -0.28835 2.16721 0.10471

H 2.22092 1.47863 0.41794

H 2.26785 -1.18171 0.40825

H -0.01891 -1.03759 1.27943

H 1.71863 -0.8255 -1.19413

H -0.1541 -2.0704 -0.10785

C -2.08449 0.00233 0.06267

H -2.09981 0.10969 1.12716

H -2.60019 0.82538 -0.38628

H -2.5671 -0.91275 -0.21051

**N-methyl-2-pyrroline**

The number of imaginary frequencies: 0

Total Energy (M06/6-311+*G*)= -250.53777695

ZPE= -250.405638

Enthalpy= -250.398822

Gibb’s Free Energy= -250.434595

C -0.02855 -0.57318 -0.15105

C 1.26736 -0.4801 -0.57951

C 1.79391 0.87643 -0.11602

C 0.48097 1.64558 -0.06848

N -0.40721 0.67078 0.57784

H -0.67354 -1.41071 -0.31672

H 1.80823 -1.21651 -1.13634

H 2.51541 1.32205 -0.76853

H 0.13582 1.83115 -1.06414

H 2.23671 0.78728 0.85396

H 0.54979 2.5825 0.44371

C -1.82735 1.03316 0.46483

H -2.08752 1.13839 -0.56772

H -2.42756 0.26588 0.90747

H -2.00069 1.95899 0.97246

**N-methyl-2-pyrroline N-oxide**

The number of imaginary frequencies: 0

Total Energy (B3LYP/6-31G*)= -325.80112215

ZPE= -325.662988

Enthalpy= -325.655506

Gibb’s Free Energy= -325.693031

C -0.03777 -0.57726 -0.15465

C 1.26273 -0.48143 -0.57967

C 1.79166 0.88052 -0.11066

C 0.47827 1.64772 -0.07115

N -0.39638 0.66679 0.57367

H -0.6877 -1.41074 -0.32137

H 1.8054 -1.21671 -1.13624

H 2.51631 1.32576 -0.75993

H 0.13792 1.82472 -1.07001

H 2.23026 0.79201 0.8613

H 0.5341 2.58902 0.43455

C -1.81636 1.03147 0.46606

H -2.07939 1.14064 -0.56534

H -2.4163 0.26345 0.90778

H -1.98697 1.95575 0.97742

O -0.09195 0.52844 1.89192

**N-methyl-2-pyrroline N-oxide**

The number of imaginary frequencies: 0

Total Energy (B3LYP/6-31G**)= -325.81385944

ZPE= -325.676246

Enthalpy= -325.668741

Gibb’s Free Energy= -325.706303

C -0.23379 1.18102 -0.17993

C -1.52502 0.87048 -0.19439

C -1.72932 -0.61116 0.01612

C -0.32087 -1.17138 -0.21707

N 0.61792 0.00123 0.08597

H 0.2688 2.13771 -0.18968

H -2.33435 1.58937 -0.23912

H -2.45724 -1.05337 -0.67185

H -0.19276 -1.46986 -1.26185

H -2.07849 -0.80218 1.03742

H -0.00953 -1.96861 0.45469

C 1.84379 -0.04037 -0.77097

H 1.5974 -0.0178 -1.83721

H 2.44721 0.82048 -0.48615

H 2.37766 -0.95301 -0.50559

O 0.98089 0.04214 1.39687

**N-methyl-2-pyrroline N-oxide**

The number of imaginary frequencies: 0

Total Energy (M06/6-311+*G*)= -325.66847024

ZPE= -325.531815

Enthalpy= -325.524448

Gibb’s Free Energy= -325.56161

C -0.03777 -0.57726 -0.15465

C 1.26273 -0.48143 -0.57967

C 1.79166 0.88052 -0.11066

C 0.47827 1.64772 -0.07115

N -0.39638 0.66679 0.57367

H -0.6877 -1.41074 -0.32137

H 1.8054 -1.21671 -1.13624

H 2.51631 1.32576 -0.75993

H 0.13792 1.82472 -1.07001

H 2.23026 0.79201 0.8613

H 0.5341 2.58902 0.43455

C -1.81636 1.03147 0.46606

H -2.07939 1.14064 -0.56534

H -2.4163 0.26345 0.90778

H -1.98697 1.95575 0.97742

O -0.09195 0.52844 1.89192

**N-methyl-3-pyrroline**

The number of imaginary frequencies: 0

Total Energy (B3LYP/6-31G*)= -250.6603928

ZPE= -250.527109

Enthalpy= -250.520299

Gibb’s Free Energy= -250.556164

C 1.07009 -1.23529 -0.0877

C 2.59979 -1.2302 0.08945

C 3.00239 0.06358 0.08894

C 1.74564 0.93562 -0.08855

N 0.70686 0.06853 0.48636

H 0.58497 -2.04788 0.41156

H 3.22822 -2.08949 0.19723

H 4.00746 0.41463 0.19625

H 1.56874 1.11503 -1.12846

C -0.66822 0.49631 0.19132

H -0.83888 1.46456 0.61354

H -0.80863 0.53959 -0.86855

H -1.35805 -0.20383 0.6142

H 0.82239 -1.28338 -1.12752

H 1.80737 1.88035 0.41002

**N-methyl-3-pyrroline**

The number of imaginary frequencies: 0

Total Energy (B3LYP/6-31G**)= -250.67291059

ZPE= -250.540105

Enthalpy= -250.533291

Gibb’s Free Energy= -250.569157

C 0.01026 -0.11685 1.19135

C 0.01026 -1.52903 0.66633

C 0.01026 -1.52903 -0.66633

C 0.01026 -0.11685 -1.19135

N 0.32891 0.68022 0.

H 0.74953 0.05319 1.98809

H -0.01543 -2.40073 1.31123

H -0.01543 -2.40073 -1.31123

H -0.98169 0.1315 -1.62443

C -0.18554 2.03252 0.

H 0.1764 2.56965 -0.88334

H -1.29288 2.08671 0.

H 0.1764 2.56965 0.88334

H -0.98169 0.1315 1.62443

H 0.74953 0.05319 -1.98809

**N-methyl-3-pyrroline**

The number of imaginary frequencies: 0

Total Energy (M06/6-311+*G*)= -250.53030478

ZPE= -250.398839

Enthalpy= -250.392016

Gibb’s Free Energy= -250.427847

C -0.00138 -0.1147 1.17913

C -0.00138 -1.51818 0.66335

C -0.00138 -1.51818 -0.66335

C -0.00138 -0.1147 -1.17913

N 0.33927 0.66756 0.

H 0.71625 0.05989 1.99335

H -0.03111 -2.39025 1.3058

H -0.03111 -2.39025 -1.3058

H -1.00567 0.14611 -1.5787

C -0.14704 2.01858 0.

H 0.21801 2.55165 -0.88417

H -1.25452 2.07526 0.

H 0.21801 2.55165 0.88417

H -1.00567 0.14611 1.5787

H 0.71625 0.05989 -1.99335

**N-methyl-3-pyrroline N-oxide**

The number of imaginary frequencies: 0

Total Energy (B3LYP/6-31G*)= -325.80062068

ZPE= -325.662821

Enthalpy= -325.655372

Gibb’s Free Energy= -325.692895

C 1.05926 -1.23237 -0.09866

C 2.58767 -1.2308 0.1087

C 2.99275 0.071 0.10821

C 1.73505 0.93937 -0.09948

N 0.711 0.06723 0.47757

H 0.55417 -2.04303 0.38364

H 3.21271 -2.0904 0.23245

H 3.9952 0.42426 0.2315

H 1.56998 1.10051 -1.14432

C -0.66662 0.4958 0.1958

H -0.83355 1.46287 0.62216

H -0.81634 0.54199 -0.86267

H -1.35271 -0.20552 0.62279

H 0.83169 -1.27213 -1.14343

H 1.7792 1.89382 0.38215

O 0.87454 0.01685 1.82676

**N-methyl-3-pyrroline N-oxide**

The number of imaginary frequencies: 0

Total Energy (B3LYP/6-31G**)= -325.81340875

ZPE= -325.676093

Enthalpy= -325.668646

Gibb’s Free Energy= -325.706136

C 0.22103 0.28223 1.21642

C 0.22103 1.67477 0.66657

C 0.22103 1.67477 -0.66657

C 0.22103 0.28223 -1.21642

N -0.15212 -0.58721 0.

H -0.55767 0.06533 1.94953

H 0.20969 2.55257 1.30413

H 0.20969 2.55257 -1.30413

H 1.20189 -0.01004 -1.61561

C 0.60337 -1.88315 0.

H 0.28233 -2.42675 -0.88876

H 1.68864 -1.73164 0.

H 0.28233 -2.42675 0.88876

H 1.20189 -0.01004 1.61561

H -0.55767 0.06533 -1.94953

O -1.47766 -0.83814 0.

**N-methyl-3-pyrroline N-oxide**

The number of imaginary frequencies: 0

Total Energy (M06/6-311+*G*)= -325.6680588

ZPE= -325.53171

Enthalpy= -325.524342

Gibb’s Free Energy= -325.561595

C 1.05926 -1.23237 -0.09866

C 2.58767 -1.2308 0.1087

C 2.99275 0.071 0.10821

C 1.73505 0.93937 -0.09948

N 0.711 0.06723 0.47757

H 0.55417 -2.04303 0.38364

H 3.21271 -2.0904 0.23245

H 3.9952 0.42426 0.2315

H 1.56998 1.10051 -1.14432

C -0.66662 0.4958 0.1958

H -0.83355 1.46287 0.62216

H -0.81634 0.54199 -0.86267

H -1.35271 -0.20552 0.62279

H 0.83169 -1.27213 -1.14343

H 1.7792 1.89382 0.38215

O 0.87454 0.01685 1.82676

**N-methylazepane**

The number of imaginary frequencies: 0

Total Energy (B3LYP/6-31G*)= -330.51413505

ZPE= -330.297713

Enthalpy= -330.288427

Gibb’s Free Energy= -330.330325

C 0.73212 1.51003 -0.4035

C -0.71609 1.25523 0.03895

C 1.7927 0.77847 0.43621

C -0.52079 -1.191 0.31221

C 2.04124 -0.69458 0.05474

C 0.80307 -1.48614 -0.4115

N -1.26022 -0.06586 -0.27351

C -2.67256 -0.11883 0.08393

H -3.21445 0.67915 -0.4361

H -3.10036 -1.07735 -0.22923

H -2.85932 0.00024 1.1703

H -0.35545 -1.0579 1.40146

H -1.16564 -2.07111 0.20855

H 1.02594 -2.55654 -0.31156

H 0.62107 -1.31016 -1.47825

H 2.74655 1.31783 0.37304

H 1.48852 0.83963 1.4906

H 0.89962 2.59119 -0.30925

H 0.83983 1.27152 -1.46966

H -0.78672 1.46965 1.12797

H -1.36351 1.98763 -0.46024

H 2.79112 -0.74141 -0.74624

H 2.49617 -1.20044 0.91699

**N-methylazepane**

The number of imaginary frequencies: 0

Total Energy (B3LYP/6-31G**)= -330.53806647

ZPE= -330.322355

Enthalpy= -330.313145

Gibb’s Free Energy= -330.354675

C 0.7311 1.51068 -0.40214

C -0.71639 1.25629 0.04082

C 1.79174 0.77753 0.43555

C -0.51944 -1.18994 0.31525

C 2.04163 -0.69371 0.05083

C 0.80285 -1.48699 -0.41006

N -1.25882 -0.06547 -0.27216

C -2.67258 -0.12059 0.08033

H -3.21461 0.67542 -0.44073

H -3.09839 -1.07873 -0.23368

H -2.86468 -0.00198 1.16495

H -0.35201 -1.05591 1.40334

H -1.16385 -2.06989 0.21472

H 1.02568 -2.55627 -0.30792

H 0.61762 -1.31452 -1.47605

H 2.74434 1.31751 0.37445

H 1.48778 0.83602 1.48928

H 0.8994 2.59073 -0.30712

H 0.83784 1.27383 -1.468

H -0.78576 1.47129 1.12915

H -1.36395 1.98832 -0.45733

H 2.78754 -0.73783 -0.75285

H 2.50134 -1.19937 0.90949

**N-methylazepane**

The number of imaginary frequencies: 0

Total Energy (M06/6-311G+**)= -330.32162526

ZPE= -330.107821

Enthalpy= -330.09853

Gibb’s Free Energy= -330.140342

C 1.51265 0.65777 0.68028

C 1.78687 -0.75362 0.1749

C 0.55687 -1.62184 -0.03627

C 0.78962 1.53617 -0.33554

C -0.53615 -0.9995 -0.90604

C -0.73095 1.41941 -0.33053

H 0.94042 0.62182 1.62108

H 2.33902 -0.67556 -0.77611

H 0.12066 -1.891 0.93777

H -0.10723 -0.64267 -1.8541

H 1.18303 1.31841 -1.33996

H -1.12031 1.98155 -1.19752

H 2.47396 1.12315 0.93659

H 2.46827 -1.2637 0.87008

H 0.86559 -2.57467 -0.49074

H 1.02842 2.59515 -0.16187

H -1.25018 -1.7895 -1.18581

H -1.11088 1.94537 0.55708

N -1.29038 0.07892 -0.30783

C -2.04279 -0.26955 0.86647

H -2.8496 0.45522 1.02999

H -2.51372 -1.25069 0.72696

H -1.45141 -0.31833 1.80185

**N-methylazepane N-oxide**

The number of imaginary frequencies: 0

Total Energy (B3LYP/6-31G*)= -405.65530091

ZPE= -405.433782

Enthalpy= -405.424009

Gibb’s Free Energy= -405.46685

C -0.68389 -0.0867 -0.0655

C 0.86341 -0.02909 -0.0243

C 1.52087 1.3621 0.05709

C -1.21057 0.349 1.28887

C 1.10761 2.30191 1.22874

C -1.33626 1.86899 1.50908

H -1.10813 0.4975 -0.85521

H 1.16889 -0.58993 0.83421

H 1.29525 1.88657 -0.84785

H 1.22426 1.8509 2.19201

H -0.53585 -0.07286 2.00419

H -1.42434 1.98096 2.56955

H -0.94122 -1.11544 -0.20832

H 1.22986 -0.48397 -0.92079

H 2.57332 1.18802 0.14045

H -2.1988 -0.03456 1.43448

H 1.74807 3.15582 1.15428

H -2.21801 2.2191 1.01425

N -0.26066 2.75574 1.02141

C -0.38413 2.94763 -0.43077

H -1.35701 3.33045 -0.6585

H 0.35913 3.64182 -0.76328

H -0.2444 2.01031 -0.92755

O -0.45463 3.92978 1.6799

**N-methylazepane N-oxide**

The number of imaginary frequencies: 0

Total Energy (B3LYP/6-31G**)= -405.68570133

ZPE= -405.465245

Enthalpy= -405.455415

Gibb’s Free Energy= -405.498192

C -0.87731 1.5007 0.36873

C 0.51088 1.33419 -0.23712

C -2.00082 0.77897 -0.39556

C 0.32701 -1.17285 -0.5794

C -2.18916 -0.71157 -0.04683

C -0.90207 -1.5005 0.25743

N 1.14604 -0.0214 0.04394

C 2.50298 -0.03427 -0.59869

H 3.06617 0.79804 -0.17874

H 2.97609 -0.96957 -0.30406

H 2.43584 0.04876 -1.68895

H 0.10455 -0.91863 -1.62206

H 1.01517 -2.01981 -0.56491

H -1.11006 -2.57108 0.1513

H -0.57176 -1.34045 1.28934

H -2.95098 1.29611 -0.21842

H -1.80879 0.88583 -1.47256

H -1.08295 2.57776 0.37236

H -0.81883 1.18252 1.41452

H 0.49467 1.50062 -1.32195

H 1.20591 2.03715 0.22537

H -2.84025 -0.79465 0.83193

H -2.73665 -1.18964 -0.86925

O 1.2963 -0.19264 1.37965

**N-methylazepane N-oxide**

The number of imaginary frequencies: 0

Total Energy (M06/6-311G+**)= -405.47684909

ZPE= -405.258482

Enthalpy= -405.24872

Gibb’s Free Energy= -405.291518

C -0.68389 -0.0867 -0.0655

C 0.86341 -0.02909 -0.0243

C 1.52087 1.3621 0.05709

C -1.21057 0.349 1.28887

C 1.10761 2.30191 1.22874

C -1.33626 1.86899 1.50908

H -1.10813 0.4975 -0.85521

H 1.16889 -0.58993 0.83421

H 1.29525 1.88657 -0.84785

H 1.22426 1.8509 2.19201

H -0.53585 -0.07286 2.00419

H -1.42434 1.98096 2.56955

H -0.94122 -1.11544 -0.20832

H 1.22986 -0.48397 -0.92079

H 2.57332 1.18802 0.14045

H -2.1988 -0.03456 1.43448

H 1.74807 3.15582 1.15428

H -2.21801 2.2191 1.01425

N -0.26066 2.75574 1.02141

C -0.38413 2.94763 -0.43077

H -1.35701 3.33045 -0.6585

H 0.35913 3.64182 -0.76328

H -0.2444 2.01031 -0.92755

O -0.45463 3.92978 1.6799

**1-methyl-2,3,4,5-tetrahydro-1H-azepine**

The number of imaginary frequencies: 0

Total Energy (B3LYP/6-31G*)= -329.29734188

ZPE=-329.104678

Enthalpy= -329.095641

Gibb’s Free Energy= -329.137249

C -1.93668 0.79405 -0.16536

C -1.91369 -0.66619 0.29955

C -0.79253 -1.50046 -0.32623

C -0.69176 1.60985 0.07132

C 0.60281 -1.21219 0.23494

C 0.60875 1.26272 0.01777

H -2.16893 0.8014 -1.24481

H -1.81819 -0.70387 1.39418

H -0.7804 -1.37039 -1.41717

H 0.55268 -1.24755 1.34067

H -2.78411 1.30138 0.31486

H -2.88152 -1.12282 0.05332

H -0.99385 -2.56353 -0.13769

H -0.86179 2.67477 0.21549

H 1.27277 -2.02244 -0.07532

H 1.32978 2.07134 0.1237

N 1.23158 0.03078 -0.20714

C 2.67576 0.02755 -0.00353

H 3.11431 0.93087 -0.43659

H 2.96525 -0.02091 1.0617

H 3.11702 -0.83567 -0.51311

**1-methyl-2,3,4,5-tetrahydro-1H-azepine**

The number of imaginary frequencies: 0

Total Energy (B3LYP/6-31G**)= -329.31548004

ZPE=-329.123508

Enthalpy= -329.114461

Gibb’s Free Energy= -329.156073

C -1.93249 0.79521 -0.17278

C -1.91545 -0.66385 0.29603

C -0.79336 -1.50269 -0.32111

C -0.69029 1.61075 0.07493

C 0.60107 -1.21171 0.23984

C 0.60955 1.26312 0.02435

H -2.15202 0.79989 -1.25399

H -1.826 -0.69823 1.39045

H -0.77885 -1.37979 -1.41203

H 0.54996 -1.2447 1.34503

H -2.78407 1.30421 0.29592

H -2.88213 -1.11899 0.04648

H -0.99535 -2.56362 -0.12654

H -0.86042 2.67389 0.22642

H 1.27056 -2.02283 -0.06704

H 1.33035 2.07028 0.13838

N 1.22934 0.03054 -0.20594

C 2.67513 0.02562 -0.01156

H 3.11207 0.92845 -0.44494

H 2.97241 -0.02535 1.05051

H 3.11319 -0.83562 -0.52531

**1-methyl-2,3,4,5-tetrahydro-1H-azepine**

The number of imaginary frequencies: 0

Total Energy (M06/6-311G+**)= -329.12249802

ZPE=-328.932494

Enthalpy= -328.923377

Gibb’s Free Energy= -328.965286

C -1.93321 0.77636 -0.14385

C -1.88554 -0.6686 0.31918

C -0.78336 -1.47754 -0.34095

C -0.69705 1.5925 0.05953

C 0.60182 -1.20425 0.21188

C 0.5986 1.25289 -0.00482

H -2.18579 0.77908 -1.21868

H -1.7509 -0.70474 1.41034

H -0.78202 -1.3067 -1.42726

H 0.55727 -1.23898 1.31985

H -2.7735 1.28283 0.34757

H -2.85654 -1.13455 0.10971

H -0.98156 -2.54636 -0.19233

H -0.86444 2.65919 0.18934

H 1.27299 -2.01417 -0.09815

H 1.31253 2.07096 0.08133

N 1.22736 0.03279 -0.21823

C 2.65472 0.03368 0.02568

H 3.1068 0.94119 -0.38139

H 2.90006 -0.0243 1.10077

H 3.11779 -0.8232 -0.47342

**1-methyl-2,3,4,5-tetrahydro-1H-azepine N-oxide**

The number of imaginary frequencies: 0

Total Energy (B3LYP/6-31G*)= -404.42502271

ZPE=-404.228193

Enthalpy= -404.218652

Gibb’s Free Energy= -404.26106

C 0.49565 0.4599 -0.25257

C 1.9606 0.36039 0.11322

C 2.62584 1.76027 0.11912

C -0.31101 1.01637 0.91325

C 2.07129 2.81315 1.12848

C -0.36455 2.31347 1.21761

H 0.40807 1.08914 -1.11355

H 2.05677 -0.11513 1.06691

H 2.5273 2.17619 -0.8618

H 2.12361 2.40779 2.11735

H 0.10558 -0.51126 -0.47523

H 2.46182 -0.22127 -0.63201

H 3.65685 1.60579 0.36006

H -0.87414 0.33125 1.5119

H 2.7101 3.66688 1.03929

H -1.22433 2.65914 1.75256

N 0.67244 3.30432 0.89169

C 0.52912 3.73516 -0.50642

H -0.45373 4.12962 -0.65912

H 1.25453 4.49139 -0.72275

H 0.68223 2.89808 -1.15508

O 0.47962 4.35813 1.72949

**1-methyl-2,3,4,5-tetrahydro-1H-azepine N-oxide**

The number of imaginary frequencies: 0

Total Energy (B3LYP/6-31G**)= -404.44322686

ZPE=-404.247194

Enthalpy= -404.237619

Gibb’s Free Energy= -404.280085

C 1.96356 0.81312 0.33863

C 2.10879 -0.60245 -0.23877

C 0.95175 -1.54601 0.10421

C 0.81163 1.62637 -0.20456

C -0.39354 -1.20835 -0.53642

C -0.47022 1.30415 -0.36417

H 1.87606 0.74597 1.43407

H 2.21458 -0.53759 -1.32906

H 0.8498 -1.63399 1.19366

H -0.30582 -1.05072 -1.61285

H 2.89003 1.36631 0.15354

H 3.04175 -1.03582 0.14115

H 1.20446 -2.55412 -0.24839

H 1.0502 2.64764 -0.4964

H -1.11915 -2.00795 -0.38101

H -1.21661 1.9886 -0.74614

N -1.16189 0.01815 -0.06544

C -1.45306 -0.0504 1.41038

H -2.09483 0.80177 1.62617

H -2.01237 -0.97213 1.57198

H -0.5458 -0.01981 2.02088

O -2.35157 0.01453 -0.74067

**1-methyl-2,3,4,5-tetrahydro-1H-azepine N-oxide**

The number of imaginary frequencies: 0

Total Energy (M06/6-311G+**)= -404.2475018

ZPE=-404.053202

Enthalpy= -404.043727

Gibb’s Free Energy=-404.085774

C 1.77906 0.80797 0.47768

C 2.12165 -0.49627 -0.20889

C 0.96929 -1.52143 -0.05614

C 0.75836 1.59069 -0.33779

C -0.4141 -1.14154 -0.66999

C -0.54738 1.32049 -0.33598

H 1.399 0.58295 1.45227

H 2.33546 -0.30886 -1.24042

H 0.81786 -1.69384 0.98897

H -0.28559 -0.92713 -1.71039

H 2.65349 1.41851 0.56438

H 2.98187 -0.91903 0.26673

H 1.2937 -2.41794 -0.54186

H 1.10631 2.40827 -0.93395

H -1.03706 -2.00156 -0.5389

H -1.21606 2.1179 -0.5848

N -1.14134 0.00879 -0.03599

C -1.20649 -0.18842 1.41927

H -1.76721 0.60792 1.86236

H -1.68405 -1.12208 1.63164

H -0.21563 -0.19464 1.82306

O -2.39439 0.01988 -0.56451

**1-methyl-2,3,4,7-tetrahydro-1H-azepine**

The number of imaginary frequencies: 0

Total Energy (B3LYP/6-31G*)= -329.28700224

ZPE=-329.094545

Enthalpy= -329.085733

Gibb’s Free Energy= -329.126397

C 0.46295 0.17234 0.24131

C 1.97537 0.39078 0.04423

C 2.50256 1.83166 -0.01181

C -0.37683 0.88382 1.02888

C 2.13658 2.73783 1.18466

C -0.37265 2.36391 1.46212

H 2.45636 -0.09513 0.8673

H 2.13696 2.3141 -0.89413

H 2.30148 2.24591 2.12046

H -0.43758 2.35657 2.53013

H 0.04036 -0.67463 -0.25765

H 2.25285 -0.06973 -0.88088

H 3.56837 1.74437 -0.04804

H -1.18608 0.31194 1.43255

H 2.76419 3.60186 1.11792

H -1.23298 2.83435 1.03385

N 0.75876 3.21509 1.05177

C 0.62038 3.49949 -0.38381

H -0.33935 3.93424 -0.57039

H 1.38631 4.1821 -0.68762

H 0.71346 2.58915 -0.93835

**1-methyl-2,3,4,7-tetrahydro-1H-azepine**

The number of imaginary frequencies: 0

Total Energy (B3LYP/6-31G**)= -329.30507753

ZPE=-329.113362

Enthalpy= -329.104525

Gibb’s Free Energy= -329.145233

C -1.51433 0.99972 -0.29773

C -1.85588 -0.45955 0.03708

C -0.7246 -1.48048 -0.17996

C -0.38439 1.68338 -0.01036

C 0.52496 -1.30198 0.70533

C 0.87964 1.19436 0.72044

H -2.16918 -0.50204 1.05931

H -0.41509 -1.44656 -1.20366

H 0.23039 -1.23137 1.73156

H 0.68845 1.21373 1.77305

H -2.27829 1.55365 -0.80214

H -2.65614 -0.75274 -0.60986

H -1.13997 -2.43881 0.05236

H -0.37641 2.71001 -0.31182

H 1.1295 -2.17173 0.55372

H 1.66412 1.87817 0.47169

N 1.36727 -0.14493 0.35048

C 1.56196 -0.17639 -1.10623

H 2.22667 0.61105 -1.39432

H 1.98159 -1.11897 -1.38971

H 0.61935 -0.04415 -1.59499

**1-methyl-2,3,4,7-tetrahydro-1H-azepine**

The number of imaginary frequencies: 0

Total Energy (M06/6-311G+**)= -329.11354081

ZPE=-328.923838

Enthalpy= -328.915004

Gibb’s Free Energy= -328.955568

C 0.46295 0.17234 0.24131

C 1.97537 0.39078 0.04423

C 2.50256 1.83166 -0.01181

C -0.37683 0.88382 1.02888

C 2.13658 2.73783 1.18466

C -0.37265 2.36391 1.46212

H 2.45636 -0.09513 0.8673

H 2.13696 2.3141 -0.89413

H 2.30148 2.24591 2.12046

H -0.43758 2.35657 2.53013

H 0.04036 -0.67463 -0.25765

H 2.25285 -0.06973 -0.88088

H 3.56837 1.74437 -0.04804

H -1.18608 0.31194 1.43255

H 2.76419 3.60186 1.11792

H -1.23298 2.83435 1.03385

N 0.75876 3.21509 1.05177

C 0.62038 3.49949 -0.38381

H -0.33935 3.93424 -0.57039

H 1.38631 4.1821 -0.68762

H 0.71346 2.58915 -0.93835

**1-methyl-2,3,4,7-tetrahydro-1H-azepine N-oxide**

The number of imaginary frequencies: 0

Total Energy (B3LYP/6-31G*)= -404.43129113

ZPE=-404.234067

Enthalpy= -404.224616

Gibb’s Free Energy= -404.266611

C 0.45874 0.16613 0.2198

C 1.97398 0.36932 0.06143

C 2.50955 1.80472 0.00757

C -0.37234 0.87672 1.00869

C 2.14031 2.74606 1.1813

C -0.34893 2.34664 1.4712

H 2.4313 -0.11719 0.89753

H 2.15919 2.27549 -0.88715

H 2.30708 2.2883 2.13395

H -0.37312 2.30691 2.54019

H 0.03122 -0.66405 -0.30263

H 2.27038 -0.09692 -0.8549

H 3.57512 1.70908 -0.01004

H -1.19202 0.30979 1.39806

H 2.77068 3.60456 1.07872

H -1.22489 2.82669 1.0876

N 0.76135 3.22591 1.04414

C 0.60023 3.53211 -0.38456

H -0.36828 3.95516 -0.55165

H 1.35178 4.23135 -0.68644

H 0.6999 2.63269 -0.95554

O 0.62535 4.34769 1.80092

**1-methyl-2,3,4,7-tetrahydro-1H-azepine N-oxide**

The number of imaginary frequencies: 0

Total Energy (B3LYP/6-31G**)= -404.44946699

ZPE=-404.253037

Enthalpy= -404.243554

Gibb’s Free Energy= -404.285602

C 1.92307 0.87597 0.11409

C 2.09044 -0.62982 -0.10959

C 0.90465 -1.50203 0.33143

C 0.83399 1.6357 -0.12024

C -0.4226 -1.28929 -0.42733

C -0.54165 1.21328 -0.66868

H 2.27679 -0.7957 -1.1501

H 0.72165 -1.33798 1.37282

H -0.2463 -1.3535 -1.48075

H -0.45947 1.11461 -1.73094

H 2.78523 1.39413 0.47889

H 2.93107 -0.94405 0.47308

H 1.2025 -2.51523 0.15937

H 0.94975 2.68046 0.07972

H -1.07357 -2.0774 -0.11109

H -1.2242 1.99791 -0.41691

N -1.1286 -0.02679 -0.1228

C -1.22486 0.09889 1.33866

H -1.79047 0.97314 1.58497

H -1.71157 -0.76551 1.73969

H -0.24261 0.18052 1.75509

O -2.36594 -0.12909 -0.67789

**1-methyl-2,3,4,7-tetrahydro-1H-azepine N-oxide**

The number of imaginary frequencies: 0

Total Energy (M06/6-311G+**)= -404.25465228

ZPE=-404.059795

Enthalpy= -404.050431

Gibb’s Free Energy=-404.092144

C 0.45874 0.16613 0.2198

C 1.97398 0.36932 0.06143

C 2.50955 1.80472 0.00757

C -0.37234 0.87672 1.00869

C 2.14031 2.74606 1.1813

C -0.34893 2.34664 1.4712

H 2.4313 -0.11719 0.89753

H 2.15919 2.27549 -0.88715

H 2.30708 2.2883 2.13395

H -0.37312 2.30691 2.54019

H 0.03122 -0.66405 -0.30263

H 2.27038 -0.09692 -0.8549

H 3.57512 1.70908 -0.01004

H -1.19202 0.30979 1.39806

H 2.77068 3.60456 1.07872

H -1.22489 2.82669 1.0876

N 0.76135 3.22591 1.04414

C 0.60023 3.53211 -0.38456

H -0.36828 3.95516 -0.55165

H 1.35178 4.23135 -0.68644

H 0.6999 2.63269 -0.95554

O 0.62535 4.34769 1.80092

**1-methyl-2,3,6,7-tetrahydro-1H-azepine**

The number of imaginary frequencies: 0

Total Energy (B3LYP/6-31G*)= -329.28622475

ZPE=-329.093718

Enthalpy= -329.084945

Gibb’s Free Energy= -329.125486

C 0.65622 0.04817 0.22592

C 1.80932 0.47645 -0.33881

C 2.60467 1.76621 -0.02668

C -0.01152 0.68273 1.44487

C 2.19411 2.5403 1.25704

C -0.26028 2.20759 1.41592

H 2.46107 2.44806 -0.83869

H 2.22732 1.94966 2.14863

H 0.62496 0.45232 2.27355

H -0.52473 2.45334 2.42318

H 0.2084 -0.83519 -0.17907

H 2.23622 -0.17011 -1.07679

H 3.63467 1.48634 0.04875

H -0.97652 0.23944 1.57593

H 2.87517 3.35964 1.3557

H -1.06514 2.43583 0.74883

N 0.8605 3.06522 1.00312

C 0.85443 3.27624 -0.45164

H -0.07965 3.7075 -0.74559

H 1.65233 3.93692 -0.71954

H 0.9862 2.33791 -0.9487

**1-methyl-2,3,6,7-tetrahydro-1H-azepine**

The number of imaginary frequencies: 0

Total Energy (B3LYP/6-31G**)= -329.30420567

ZPE= -329.112519

Enthalpy= -329.103711

Gibb’s Free Energy= -329.144319

C 1.99797 -0.4302 -0.07796

C 1.76928 0.8603 -0.34826

C 0.45115 1.58846 -0.22273

C 0.97454 -1.48002 0.30279

C -0.51524 0.94658 0.79829

C -0.42038 -1.271 -0.29984

H -0.03894 1.68785 -1.20393

H 0.0587 0.71542 1.70449

H 0.89381 -1.58222 1.39549

H -0.97602 -2.21782 -0.25329

H 3.0231 -0.79045 -0.16407

H 2.61791 1.47108 -0.65701

H 0.65873 2.61831 0.10046

H 1.34125 -2.45151 -0.05297

H -1.27386 1.68024 1.09353

H -0.29162 -1.03931 -1.37605

N -1.23345 -0.25848 0.37624

C -2.43499 0.02608 -0.39246

H -2.9834 -0.90701 -0.56853

H -3.09029 0.69863 0.17161

H -2.23924 0.48507 -1.38242

**1-methyl-2,3,6,7-tetrahydro-1H-azepine**

The number of imaginary frequencies: 0

Total Energy (M06/6-311G+**)= -329.11349961

ZPE=-328.923679

Enthalpy= -328.914937

Gibb’s Free Energy= -328.955271

C 1.98583 -0.41519 -0.07008

C 1.7457 0.86549 -0.34366

C 0.42498 1.56747 -0.23043

C 0.97413 -1.46395 0.2974

C -0.51329 0.93413 0.79927

C -0.40349 -1.25533 -0.30763

H -0.07178 1.63208 -1.21126

H 0.06905 0.7131 1.70383

H 0.88737 -1.5684 1.38944

H -0.95658 -2.20448 -0.29285

H 3.01627 -0.76022 -0.14325

H 2.59056 1.48325 -0.64527

H 0.61224 2.60884 0.06172

H 1.34832 -2.43297 -0.05422

H -1.27575 1.66095 1.10211

H -0.27248 -0.99307 -1.37912

N -1.21805 -0.26762 0.3788

C -2.41151 0.01528 -0.38202

H -2.94669 -0.91825 -0.58815

H -3.08156 0.66692 0.18734

H -2.20669 0.49821 -1.35893

**1-methyl-2,3,6,7-tetrahydro-1H-azepine N-oxide**

The number of imaginary frequencies: 0

Total Energy (B3LYP/6-31G*)= -404.42326899

ZPE=-404.225979

Enthalpy= -404.216486

Gibb’s Free Energy= -404.259077

C 0.63577 0.05068 0.19107

C 1.81061 0.46269 -0.32535

C 2.616 1.74208 0.00561

C -0.01818 0.66913 1.41353

C 2.20367 2.57041 1.26497

C -0.23454 2.19901 1.44017

H 2.50056 2.40935 -0.82284

H 2.24317 2.03388 2.18989

H 0.61702 0.39986 2.23141

H -0.43813 2.40267 2.47069

H 0.16758 -0.80395 -0.25087

H 2.25465 -0.192 -1.04585

H 3.63859 1.44571 0.11224

H -0.99328 0.2458 1.53552

H 2.88742 3.39231 1.308

H -1.06983 2.46574 0.82695

N 0.86699 3.07773 0.99765

C 0.83582 3.27861 -0.45823

H -0.1133 3.68284 -0.74226

H 1.61213 3.95846 -0.74117

H 0.98595 2.34076 -0.95097

O 0.65027 4.25335 1.64615

**1-methyl-2,3,6,7-tetrahydro-1H-azepine N-oxide**

The number of imaginary frequencies: 0

Total Energy (B3LYP/6-31G**)= -404.44160371

ZPE=-404.245096

Enthalpy= -404.23558

Gibb’s Free Energy= -404.278124

C 2.00147 0.51596 0.29064

C 1.72629 -0.78975 0.30569

C 0.67339 -1.51602 -0.58728

C 1.07751 1.51412 -0.35375

C -0.55367 -0.67522 -1.07595

C -0.41156 1.41351 0.09795

H 0.29215 -2.31051 0.01964

H -0.3307 0.01214 -1.86508

H 1.14759 1.35966 -1.41022

H -0.93173 2.05724 -0.58024

H 2.91412 0.85939 0.73113

H 2.29074 -1.39738 0.98178

H 1.15724 -1.90733 -1.45772

H 1.39723 2.50376 -0.10218

H -1.27141 -1.39032 -1.42004

H -0.52046 1.75918 1.1047

N -1.05058 0.07145 0.04715

C -0.77633 -0.74779 1.23649

H -1.12619 -0.23751 2.10948

H -1.28047 -1.68727 1.14643

H 0.2773 -0.9149 1.31926

O -2.3855 0.31311 -0.04871

**1-methyl-2,3,6,7-tetrahydro-1H-azepine N-oxide**

The number of imaginary frequencies: 0

Total Energy (M06/6-311G+**)= -404.25593024

ZPE=-404.061382

Enthalpy= -404.05197

Gibb’s Free Energy=-404.093798

C -2.13243 -0.62773 -0.00619

C -2.1129 0.69917 0.0899

C -0.89012 1.57286 0.06504

C -0.93812 -1.53555 -0.02644

C 0.26186 1.00473 -0.74248

C 0.2522 -1.00399 0.73689

H -0.57255 1.84981 1.08181

H -0.10086 0.52604 -1.65426

H -0.61122 -1.78372 -1.04644

H 0.94894 -1.80485 0.99417

H -3.1038 -1.11763 -0.04322

H -3.06576 1.22109 0.15518

H -1.16244 2.52684 -0.4037

H -1.22239 -2.49315 0.42425

H 0.97609 1.77506 -1.04426

H -0.06828 -0.49279 1.65335

N 1.10204 -0.06467 -0.08457

C 2.08847 0.57282 0.8329

H 2.6448 -0.23081 1.31627

H 2.76704 1.15301 0.20788

H 1.59677 1.20693 1.57869

O 1.76069 -0.74713 -1.04068

**Triplet Oxygen**

The number of imaginary frequencies: 0

Total Energy (B3LYP/6-31G*)= -75.06062312

ZPE= -75.060623

Enthalpy= -75.058263

Gibb’s Free Energy= -75.075575

O 0. 0. 0.

**Triplet Oxygen**

The number of imaginary frequencies: 0

Total Energy (B3LYP/6-31G**)= -75.06062312

ZPE= -75.060623

Enthalpy= -75.058263

Gibb’s Free Energy= -75.075575

O 0. 0. 0.

**Triplet Oxygen**

The number of imaginary frequencies: 0

Total Energy (M06/6-311G+**)= -75.05344297

ZPE= -75.053443

Enthalpy= -75.051083

Gibb’s Free Energy= -75.068395

O 0. 0. 0.

**Singlet Oxygen**

The number of imaginary frequencies: 0

Total Energy (B3LYP/6-31G*)= -74.95739789

ZPE= -74.957398

Enthalpy= -74.955037

Gibb’s Free Energy= -74.971313

O 0. 0. 0.

**Singlet Oxygen**

The number of imaginary frequencies: 0

Total Energy (B3LYP/6-31G**)= -74.95739789

ZPE= -74.957398

Enthalpy= -74.955037

Gibb’s Free Energy= -74.971313

O 0. 0. 0.

**Singlet Oxygen**

The number of imaginary frequencies: 0

Total Energy (M06/6-311G+**)= -74.95543188

ZPE= -74.955432

Enthalpy= -74.953071

Gibb’s Free Energy= -74.969347

O 0. 0. 0.

**7-methyl-7-azanorcaradiene**

The number of imaginary frequencies: 0

Total Energy (B3LYP/6-31G*)=-326.83454058

ZPE= -326.689984

Enthalpy=-326.682268

Gibb’s Free Energy= -326.720491

C 1.96758 -0.78106 -0.2494

C 1.9667 0.61802 -0.34996

C 0.88802 1.40813 -0.02297

C -0.41571 0.93722 0.70222

C -0.35664 -0.57731 0.48024

C 0.86155 -1.41833 0.14091

H 2.85043 -1.33594 -0.48935

H 2.85186 1.09785 -0.71211

H 0.94559 2.44192 -0.29287

H 0.83621 -2.48521 0.21865

H -0.39413 0.92431 1.77192

H -0.82068 -1.13466 1.26695

N -1.35975 0.10157 -0.24957

C -2.7971 -0.13006 -0.45273

H -3.23944 0.73849 -0.89414

H -2.93485 -0.96948 -1.1018

H -3.26323 -0.3278 0.48988

**7-methyl-7-azanorcaradiene**

The number of imaginary frequencies: 0

Total Energy (B3LYP/6-31G**)= -326.84784237

ZPE= -326.703582

Enthalpy= -326.695868

Gibb’s Free Energy= -326.734078

C 1.86658 -0.72695 -0.30896

C 1.86652 0.72697 -0.30892

C 0.82595 1.43776 0.17935

C -0.43635 0.77203 0.53789

C -0.43622 -0.77204 0.53792

C 0.82604 -1.43774 0.17938

H 2.78195 -1.23639 -0.59808

H 2.78187 1.23649 -0.59798

H 0.91474 2.50678 0.35361

H 0.91476 -2.50677 0.35368

H -1.07173 1.2685 1.2729

H -1.07168 -1.26843 1.27288

N -1.12942 0.00003 -0.49691

C -2.58389 -0.00006 -0.38983

H -2.97899 0.88754 -0.89449

H -2.97884 -0.88773 -0.89446

H -2.95796 -0.00007 0.64929

**7-methyl-7-azanorcaradiene**

The number of imaginary frequencies: 0

Total Energy (M06/6-311G+**)= -326.6688665

ZPE= -326.525862

Enthalpy= -326.51812

Gibb’s Free Energy= -326.556372

C 1.86736 -0.72717 -0.30848

C 1.86727 0.72699 -0.3085

C 0.82599 1.43804 0.17889

C -0.43647 0.77206 0.53738

C -0.43635 -0.77192 0.53743

C 0.82597 -1.43801 0.179

H 2.78349 -1.23668 -0.59757

H 2.78347 1.23657 -0.5974

H 0.91428 2.50789 0.35285

H 0.91386 -2.5079 0.35303

H -1.07253 1.26895 1.27227

H -1.07282 -1.26867 1.272

N -1.13026 0.0002 -0.49695

C -2.58436 -0.00015 -0.38897

H -2.97867 0.88861 -0.89404

H -2.97838 -0.88884 -0.89439

H -2.95739 -0.0004 0.65145

**7-methyl-7-azanorcaradiene N-oxide**

The number of imaginary frequencies: 0

Total Energy (B3LYP/6-31G*)= -401.97620874

ZPE= -401.828039

Enthalpy=-401.81928

Gibb’s Free Energy= -401.859745

C 1.90702 -0.76695 -0.26941

C 1.92231 0.77444 -0.25264

C 0.86515 1.47579 0.22253

C -0.47927 0.78862 0.52423

C -0.47307 -0.74424 0.56603

C 0.82831 -1.46426 0.16868

H 2.76561 -1.29679 -0.62576

H 2.78738 1.29707 -0.60395

H 0.96946 2.52581 0.39994

H 0.87985 -2.53127 0.22968

H -0.99883 1.39917 1.23287

H -1.02755 -1.30025 1.29287

N -1.19431 -0.01676 -0.49064

C -2.6622 -0.01992 -0.41196

H -3.0487 0.83602 -0.92467

H -3.0419 -0.91034 -0.86789

H -2.96445 0.01226 0.61396

O -0.81195 -0.0583 -1.79513

**7-methyl-7-azanorcaradiene N-oxide**

The number of imaginary frequencies: 0

Total Energy (B3LYP/6-31G**)= -401.98966351

ZPE= -401.841848

Enthalpy= -401.83308

Gibb’s Free Energy= -401.873557

C 1.90489 0.72696 0.28203

C 1.90523 -0.72705 0.28062

C 0.94617 -1.43689 -0.35466

C -0.25337 -0.76724 -0.84109

C -0.2525 0.76857 -0.84149

C 0.94613 1.43748 -0.35318

H 2.76352 1.23737 0.70874

H 2.76448 -1.23784 0.70567

H 1.02988 -2.51044 -0.48731

H 1.02931 2.51139 -0.48328

H -0.87801 -1.28311 -1.56504

H -0.87785 1.28469 -1.56453

N -1.06841 -0.00086 0.23813

C -2.53029 0.00055 0.01848

H -2.92878 -0.89135 0.50462

H -2.92741 0.89181 0.50682

H -2.77843 0.00199 -1.04642

O -0.71442 -0.00159 1.48871

**7-methyl-7-azanorcaradiene N-oxide**

The number of imaginary frequencies: 0

Total Energy (M06/6-311G+**)= -401.80644197

ZPE= -401.659005

Enthalpy= -401.650406

Gibb’s Free Energy= -401.690561

C -1.9366 -0.69675 0.32594

C -1.91983 0.72422 0.30748

C -0.97434 1.43326 -0.37774

C 0.29984 0.74459 -0.87686

C 0.29666 -0.79492 -0.8178

C -0.99917 -1.44498 -0.32655

H -2.72311 -1.19699 0.85135

H -2.68794 1.25638 0.82873

H -1.12616 2.47491 -0.56963

H -1.17031 -2.48784 -0.49408

H 0.79835 1.24075 -1.68322

H 0.73905 -1.36195 -1.61006

N 1.09013 -0.00025 0.11397

C 2.56009 -0.00873 0.1194

H 2.92013 0.87937 0.59538

H 2.90984 -0.86691 0.65429

H 2.92028 -0.04482 -0.88751

O 0.72863 0.04609 1.42422

**Pyridine**

The number of imaginary frequencies: 0

Total Energy (B3LYP/6-31G**)= -248.29259287

ZPE= -248.203718

Enthalpy= -248.198501

Gibb’s Free Energy= -248.231119 C -1.14249 -0.72173 -0.00022

C -1.19869 0.67323 -0.00012

C 0.0002 1.38545 0.0001

C 1.19888 0.67291 0.00022

C 1.14229 -0.72204 0.00013

N -0.0002 -1.42093 -0.0001

H 0.00032 2.47233 0.00016

H -2.05988 -1.30864 -0.00037

H -2.15775 1.18284 -0.00023

H 2.1581 1.18221 0.00036

H 2.05951 -1.3092 0.00019

**Pyridine N-oxide**

The number of imaginary frequencies: 0

Total Energy (B3LYP/6-31G**)= -323.45500559

ZPE= -323.361911

Enthalpy= -323.355908

Gibb’s Free Energy= -323.390607

C 0.28383 -1.18039 -0.00018

C -1.09931 -1.19458 -0.00009

C -1.82222 0. 0.00012

C -1.09931 1.19458 0.00024

C 0.28383 1.18039 0.00014

N 0.98713 0. -0.00007

H -2.90654 0. 0.0002

H 0.91433 -2.05913 -0.00034

H -1.60303 -2.15629 -0.00019

H -1.60303 2.15629 0.00041

H 0.91433 2.05913 0.00022

O 2.26163 0. -0.00015

**Trimethylamine**

The number of imaginary frequencies: 0

Total Energy (B3LYP/6-31G**)= -174.4861591

ZPE= -174.365595

Enthalpy= -174.359219

Gibb’s Free Energy= -174.391860

N 0. 0. 0.37142

C 0. 1.38908 -0.06099

H -0.88689 1.89889 0.33175

H 0.88689 1.89889 0.33175

H 0. 1.5053 -1.16421

C -1.20298 -0.69454 -0.06099

H -1.20104 -1.71751 0.33175

H -2.08793 -0.18138 0.33175

H -1.30363 -0.75265 -1.16421

C 1.20298 -0.69454 -0.06099

H 2.08793 -0.18138 0.33175

H 1.20104 -1.71751 0.33175

H 1.30363 -0.75265 -1.16421

**Trimethylamine N-oxide**

The number of imaginary frequencies: 0

Total Energy (B3LYP/6-31G**)= -249.62754733

ZPE= -249.502133

Enthalpy= -249.495143

Gibb’s Free Energy= -249.529500

N 0. 0. 0.08312

C 0. 1.41754 -0.41881

H -0.88839 1.89255 -0.0028

H 0.88839 1.89255 -0.0028

H 0. 1.4678 -1.51383

C -1.22763 -0.70877 -0.41881

H -1.1948 -1.71564 -0.0028

H -2.08319 -0.1769 -0.0028

H -1.27115 -0.7339 -1.51383

C 1.22763 -0.70877 -0.41881

H 2.08319 -0.1769 -0.0028

H 1.1948 -1.71564 -0.0028

H 1.27115 -0.7339 -1.51383

O 0. 0. 1.43938

**N-methylaziridine**

The number of imaginary frequencies: 0

Total Energy (B3LYP/6-31G*)= -173.23135212

ZPE= -173.13292

Enthalpy= -173.127558

Gibb’s Free Energy= -173.159205

C -0.81263 0.01988 -0.00942

C 0.69188 0.01646 0.01901

H -1.31927 -0.34205 -0.8796

H -1.36422 -0.28283 0.85604

H 1.30576 -0.2704 -0.80909

H 1.16767 -0.2429 0.94165

N -0.06563 1.32111 -0.03859

C -0.13378 2.19462 -1.21895

H -1.01422 2.80019 -1.16415

H 0.73094 2.82418 -1.24716

H -0.16768 1.59529 -2.1047

**N-methylaziridine N-oxide**

The number of imaginary frequencies: 0

Total Energy (B3LYP/6-31G*)= -248.36984065

ZPE= -248.267481

Enthalpy= -248.261373

Gibb’s Free Energy= -248.295118

C 0.91154 0.77392 0.11511

C 0.89824 -0.77227 0.10721

H 0.7465 1.28391 1.04117

H 1.58558 1.27929 -0.54457

H 0.78061 -1.29457 1.03364

H 1.59987 -1.28788 -0.51468

N -0.20087 0.00088 -0.47225

C -1.55546 0.00289 0.09869

H -2.07808 0.87829 -0.22604

H -2.08198 -0.86901 -0.22913

H -1.49231 0.00086 1.16682

O -0.38148 0.00368 -1.8202

**N-methylaziridine**

The number of imaginary frequencies: 0

Total Energy (B3LYP/6-31G**)= -173.23135212

ZPE= -173.13292

Enthalpy= -173.127558

Gibb’s Free Energy= -173.159205

C 0.9011 0.74593 0.12103

C 0.90182 -0.74557 0.12108

H 0.65933 1.24157 1.06284

H 1.57628 1.30308 -0.52521

H 0.66068 -1.24143 1.06294

H 1.57806 -1.30166 -0.52499

N -0.1564 -0.00029 -0.55436

C -1.45929 -0.00019 0.09732

H -2.01925 0.88759 -0.21681

H -2.0191 -0.88812 -0.2166

H -1.40297 0.00001 1.20182

**N-methylaziridine N-oxide**

The number of imaginary frequencies: 0

Total Energy (B3LYP/6-31G**)= -248.36984065

ZPE= -248.267481

Enthalpy= -248.261373

Gibb’s Free Energy= -248.295118

C 0.91197 0.77358 0.11543

C 0.89812 -0.77261 0.10775

H 0.74695 1.28376 1.04138

H 1.58629 1.27861 -0.54422

H 0.78015 -1.29473 1.03424

H 1.59968 -1.28856 -0.51395

N -0.20061 0.00085 -0.47201

C -1.5553 0.00342 0.0987

H -2.07756 0.87895 -0.22625

H -2.08207 -0.86834 -0.22908

H -1.49234 0.00152 1.16684

O -0.38099 0.0035 -1.81999

**N-methylaziridine**

The number of imaginary frequencies: 0

Total Energy (M06/6-311G+**)= -173.14757437

ZPE= -173.050266

Enthalpy= -173.044925

Gibb’s Free Energy= -173.076507

C -0.93158 -0.75352 0.1074

C -0.93188 0.74813 0.12172

H -0.69467 -1.35042 0.96325

H -1.68532 -1.25779 -0.46048

H -0.82027 1.35583 0.99531

H -1.61782 1.23386 -0.54044

N 0.19908 0.00654 -0.54255

C 1.51627 0.00101 0.11005

H 2.06081 -0.86878 -0.193

H 2.05878 0.87842 -0.1741

H 1.388 -0.01062 1.17227

**N-methylaziridine N-oxide**

The number of imaginary frequencies: 0

Total Energy (M06/6-311G+**)= -248.28292193

ZPE= -248.181688

Enthalpy= -248.175647

Gibb’s Free Energy= -248.209266

C 0.89389 0.78517 0.1286

C 0.91063 -0.76037 0.13715

H 0.78837 1.31758 1.05072

H 1.55257 1.28652 -0.5494

H 0.69589 -1.26128 1.05795

H 1.65022 -1.27839 -0.43692

N -0.19019 -0.00268 -0.47178

C -1.55579 -0.0167 0.07213

H -2.08411 0.84935 -0.26804

H -2.06334 -0.89781 -0.26095

H -1.51393 -0.01187 1.1413

O -0.34374 -0.00959 -1.82307

**Cartesian Coordinates (Å) and energies (Hartrees) for all structures calculated using Gaussian 16.**

**N-methylazepine N-oxide**

The number of imaginary frequencies: 0

B3LYP/6-31G**

Enthalpy = -401.987790971

6 1.969668 -0.680418 0.208142

6 1.969400 0.680840 0.208107

6 0.928373 1.505631 -0.354441

6 0.928837 -1.505655 -0.354106

6 -0.368340 1.197552 -0.538867

6 -0.368016 -1.198005 -0.538327

1 2.865300 -1.192417 0.550726

1 2.864894 1.193165 0.550561

1 1.229467 2.487553 -0.714713

1 1.230211 -2.487594 -0.714117

1 -1.084432 1.813237 -1.067255

1 -1.084025 -1.814112 -1.066334

7 -1.072525 -0.000194 -0.007446

8 -2.358751 -0.000265 -0.468970

6 -1.098760 0.000428 1.498914

1 -1.659893 -0.889641 1.780260

1 -0.094888 -0.001947 1.928068

1 -1.655926 0.893004 1.780144

**N-methylazepine N-oxide**

The number of imaginary frequencies: 0

APFD/aug-cc-pVDZ

Enthalpy = -401.683840334

6 0.267783 -0.765912 -0.850829

6 0.266149 0.768828 -0.851379

1 -1.024147 -2.511770 -0.522626

6 -0.930445 -1.434678 -0.374622

6 -0.930346 1.435990 -0.371472

6 -1.873434 0.725588 0.290486

6 -1.874281 -0.725756 0.287379

1 -1.023187 2.513828 -0.514689

1 -2.730252 1.239817 0.732943

1 -2.732553 -1.240742 0.726194

7 1.044658 -0.001765 0.246063

6 2.499037 0.001073 0.057002

1 2.886681 0.896917 0.558671

1 2.889451 -0.896205 0.554123

1 2.772801 0.004022 -1.007715

1 0.911605 -1.285576 -1.565411

1 0.911128 1.288788 -1.564505

8 0.659887 -0.003440 1.470147

**N-methylazepine N-oxide**

The number of imaginary frequencies: 0

APFD/aug-cc-pVTZ

Enthalpy = -401.788822197

6 1.936336 -0.677553 0.211900

6 1.936345 0.677535 0.211970

6 0.910417 1.491864 -0.369788

6 0.910389 -1.491833 -0.369887

6 -0.376347 1.175332 -0.561500

6 -0.376385 -1.175288 -0.561548

1 2.827436 -1.187874 0.561969

1 2.827444 1.187808 0.562110

1 1.212970 2.464927 -0.745423

1 1.212936 -2.464858 -0.745620

1 -1.089698 1.768307 -1.115084

1 -1.089730 -1.768214 -1.115195

7 -1.066001 0.000025 0.000727

8 -2.357261 0.000062 -0.397984

6 -1.018836 -0.000100 1.490928

1 -1.558371 -0.890416 1.801015

1 0.004805 0.000220 1.861470

1 -1.559211 0.889686 1.801089

**1-methyl-2,3,4,5-tetrahydro-1H-azepine N-oxide**

The number of imaginary frequencies: 0

B3LYP/6-31G**

Enthalpy= -404.439377914

6 2.023212 -0.761124 0.181705

6 1.777779 0.725632 0.493583

6 0.968236 1.412198 -0.618470

6 0.889170 -1.523008 -0.448140

6 -0.551882 1.280359 -0.480836

6 -0.405432 -1.224603 -0.528919

1 2.877818 -0.848634 -0.504799

1 2.750829 1.219588 0.596870

1 1.178119 2.488396 -0.631154

1 1.166901 -2.471881 -0.905311

1 -1.034354 1.436125 -1.452725

7 -1.047619 -0.042198 0.106373

8 -0.851457 -0.062281 1.463086

6 -2.530440 -0.120374 -0.136997

1 -2.769823 -0.148817 -1.205644

1 -2.872303 -1.020394 0.371731

1 -2.970022 0.752761 0.341584

1 -1.109400 -1.896943 -1.006396

1 2.327864 -1.292376 1.094489

1 1.233461 0.813478 1.434658

1 1.286555 1.030340 -1.596322

1 -0.944518 1.997513 0.242163

**1-methyl-2,3,4,5-tetrahydro-1H-azepine N-oxide**

The number of imaginary frequencies: 0

APFD/aug-cc-pVDZ

Enthalpy= -404.123157784

6 1.941457 -0.789644 0.253272

6 1.779678 0.726556 0.468894

6 0.962522 1.389394 -0.641095

6 0.859247 -1.497314 -0.506966

6 -0.548692 1.281066 -0.459394

6 -0.430196 -1.180479 -0.608579

1 2.884612 -0.981306 -0.288916

1 2.784230 1.180227 0.502029

1 1.188853 2.467696 -0.692664

1 1.157943 -2.407691 -1.037976

1 -1.066516 1.463152 -1.415800

7 -1.031542 -0.038921 0.113207

8 -0.752767 -0.110983 1.433507

6 -2.511481 -0.098481 -0.054054

1 -2.808945 -0.080569 -1.114444

1 -2.834200 -1.022026 0.438811

1 -2.921344 0.760541 0.487075

1 -1.134485 -1.782349 -1.184919

1 2.050641 -1.296140 1.229102

1 1.281831 0.907106 1.430062

1 1.253863 0.970370 -1.620094

1 -0.908765 1.994716 0.294767

**1-methyl-2,3,4,5-tetrahydro-1H-azepine N-oxide**

The number of imaginary frequencies: 0

APFD/aug-cc-pVTZ

Enthalpy= -404.240506936

6 1.978245 -0.771779 0.209821

6 1.777074 0.723720 0.477927

6 0.963680 1.392242 -0.627109

6 0.866795 -1.501145 -0.473464

6 -0.544562 1.273814 -0.456823

6 -0.417313 -1.195643 -0.560395

1 2.869416 -0.906540 -0.414722

1 2.761400 1.195441 0.543579

1 1.183005 2.463067 -0.668433

1 1.147926 -2.429522 -0.963314

1 -1.047170 1.443774 -1.412615

7 -1.037342 -0.040732 0.113352

8 -0.812266 -0.095833 1.449193

6 -2.507697 -0.100082 -0.110604

1 -2.752460 -0.105179 -1.175110

1 -2.855852 -1.004500 0.380636

1 -2.935467 0.765894 0.386018

1 -1.120898 -1.834517 -1.076943

1 2.198355 -1.295440 1.147590

1 1.267225 0.860499 1.430010

1 1.259835 0.983476 -1.598280

1 -0.913122 1.988571 0.278456

**1-methyl-2,3,6,7-tetrahydro-1H-azepine N-oxide**

The number of imaginary frequencies: 0

B3LYP/6-31G**

Enthalpy= -404.433978813

6 -0.576807 1.842742 0.666223

6 -0.576807 1.842742 -0.666223

6 0.508217 1.117225 -1.423688

6 0.508217 1.117225 1.423688

6 0.508217 -0.419616 -1.292730

6 0.508217 -0.419616 1.292730

1 0.449230 1.341231 -2.493823

1 1.525868 -0.802219 -1.437872

7 -0.065214 -1.005938 -0.000000

8 -1.418000 -0.893579 -0.000000

6 0.277768 -2.475051 -0.000000

1 1.359134 -2.651960 -0.000000

1 -0.190055 -2.900739 0.886380

1 -0.190055 -2.900739 -0.886380

1 1.525868 -0.802219 1.437872

1 -1.366336 2.335326 1.225810

1 -1.366336 2.335326 -1.225810

1 1.485460 1.495474 -1.093683

1 -0.154553 -0.864943 -2.034635

1 0.449230 1.341231 2.493823

1 -0.154553 -0.864943 2.034635

1 1.485460 1.495474 1.093683

**1-methyl-2,3,6,7-tetrahydro-1H-azepine N-oxide**

The number of imaginary frequencies: 0

APFD/aug-cc-pVDZ

Enthalpy=-404.118296570

6 -0.580349 1.816900 0.667109

6 -0.580349 1.816900 -0.667109

6 0.516659 1.107151 -1.410951

6 0.516659 1.107151 1.410951

6 0.516659 -0.422673 -1.278441

6 0.516659 -0.422673 1.278441

1 0.478492 1.336464 -2.487119

1 1.540076 -0.813529 -1.409237

7 -0.070970 -0.994026 -0.000000

8 -1.403662 -0.848300 -0.000000

6 0.245188 -2.455492 -0.000000

1 1.329340 -2.652048 -0.000000

1 -0.233266 -2.876859 0.890642

1 -0.233266 -2.876859 -0.890642

1 1.540076 -0.813529 1.409237

1 -1.388686 2.290435 1.228873

1 -1.388686 2.290435 -1.228873

1 1.490200 1.493395 -1.057620

1 -0.141820 -0.873389 -2.031483

1 0.478492 1.336464 2.487119

1 -0.141820 -0.873389 2.031483

1 1.490200 1.493395 1.057620

**1-methyl-2,3,6,7-tetrahydro-1H-azepine N-oxide**

The number of imaginary frequencies: 0

APFD/aug-cc-pVTZ

Enthalpy=-404.236049675

6 -0.574719 1.823334 0.662558

6 -0.574719 1.823334 -0.662558

6 0.510721 1.106581 -1.409081

6 0.510721 1.106581 1.409081

6 0.510721 -0.421084 -1.273461

6 0.510721 -0.421084 1.273461

1 0.463560 1.331838 -2.476481

1 1.528360 -0.803455 -1.394617

7 -0.071517 -0.995877 -0.000000

8 -1.410929 -0.869542 0.000000

6 0.259926 -2.450388 -0.000000

1 1.338205 -2.625857 -0.000000

1 -0.206064 -2.876044 0.884313

1 -0.206064 -2.876044 -0.884313

1 1.528360 -0.803455 1.394617

1 -1.369087 2.304794 1.219809

1 -1.369087 2.304794 -1.219809

1 1.479264 1.487992 -1.065618

1 -0.131227 -0.870277 -2.028562

1 0.463560 1.331838 2.476481

1 -0.131227 -0.870277 2.028562

1 1.479264 1.487992 1.065618

**1-methyl-2,3,4,7-tetrahydro-1H-azepine N-oxide**

The number of imaginary frequencies: 0

B3LYP/6-31G**

Enthalpy= -404.444680715

6 -1.958168 0.798791 0.156899

6 -1.884165 -0.677640 0.437836

6 -1.005620 -1.427777 -0.577975

6 -0.979128 1.559437 -0.336153

6 0.502697 -1.340071 -0.351742

6 0.427614 1.166537 -0.695513

1 -2.896859 -1.096736 0.403419

1 -1.252424 -2.496320 -0.561356

1 1.032607 -1.620590 -1.270068

7 1.016583 0.015684 0.124606

8 0.769016 0.175018 1.446679

6 2.509277 0.029320 -0.083156

1 2.780036 -0.002802 -1.144270

1 2.871169 0.938979 0.392858

1 2.909483 -0.834453 0.446194

1 0.532992 0.920207 -1.761754

1 -2.901162 1.289462 0.392927

1 -1.489531 -0.825230 1.449740

1 -1.255107 -1.074703 -1.586040

1 0.817881 -1.993295 0.462973

1 -1.176692 2.619443 -0.484723

1 1.084359 2.014525 -0.486750

**1-methyl-2,3,4,7-tetrahydro-1H-azepine N-oxide**

The number of imaginary frequencies: 0

APFD/aug-cc-pVDZ

Enthalpy= -404.129255205

6 -1.943774 0.792334 0.160870

6 -1.864537 -0.678237 0.440491

6 -0.997440 -1.416429 -0.583048

6 -0.967470 1.553729 -0.340857

6 0.504767 -1.327512 -0.356899

6 0.432762 1.153455 -0.697393

1 -2.882164 -1.102341 0.421575

1 -1.245076 -2.491298 -0.582848

1 1.043561 -1.600439 -1.279999

7 1.007662 0.015371 0.129391

8 0.738792 0.173323 1.430003

6 2.488691 0.030164 -0.062706

1 2.770414 0.012281 -1.127500

1 2.848037 0.938204 0.432983

1 2.887481 -0.844567 0.462825

1 0.536299 0.894306 -1.767247

1 -2.890611 1.288014 0.402619

1 -1.450380 -0.820152 1.453266

1 -1.252459 -1.046847 -1.591906

1 0.822725 -1.990523 0.458506

1 -1.168570 2.619345 -0.492161

1 1.098777 2.004814 -0.498624

**1-methyl-2,3,4,7-tetrahydro-1H-azepine N-oxide**

The number of imaginary frequencies: 0

APFD/aug-cc-pVTZ

Enthalpy= -404.246538534

6 -1.937752 0.796618 0.159803

6 -1.877343 -0.673056 0.427883

6 -0.995392 -1.413871 -0.575379

6 -0.964637 1.545607 -0.342658

6 0.499574 -1.331134 -0.323577

6 0.426871 1.135744 -0.707140

1 -2.890669 -1.082608 0.379541

1 -1.246806 -2.478591 -0.577394

1 1.044330 -1.624119 -1.225637

7 1.009679 0.016386 0.131013

8 0.760071 0.200090 1.436158

6 2.484110 0.024091 -0.082977

1 2.739314 -0.012614 -1.144578

1 2.855564 0.932333 0.382703

1 2.887891 -0.837101 0.442810

1 0.515419 0.851957 -1.762379

1 -2.869250 1.295662 0.412803

1 -1.503932 -0.830847 1.444706

1 -1.227361 -1.045564 -1.579855

1 0.796683 -1.977416 0.500914

1 -1.153116 2.606147 -0.478459

1 1.091029 1.983349 -0.537259

**N-methylazepane N-oxide**

The number of imaginary frequencies: 0

B3LYP/6-31G**

Enthalpy = -405.675354659

6 -0.607085 1.792740 0.777922

6 -0.607085 1.792740 -0.777922

6 0.568106 1.038282 -1.419616

6 0.568106 1.038282 1.419616

6 0.568106 -0.490602 -1.278837

6 0.568106 -0.490602 1.278837

1 -0.562496 2.826694 1.142109

1 -0.562496 2.826694 -1.142109

1 0.568826 1.238510 -2.498135

1 1.516849 1.443877 1.051641

1 1.587025 -0.881026 -1.390306

1 -0.069590 -0.940647 2.040868

7 -0.037297 -1.055132 -0.000000

8 -1.387282 -0.860707 -0.000000

6 0.213796 -2.540083 -0.000000

1 1.282444 -2.779776 -0.000000

1 -0.277798 -2.937635 0.886526

1 -0.277798 -2.937635 -0.886526

1 1.587025 -0.881026 1.390306

1 0.568826 1.238510 2.498135

1 -1.540526 1.353638 1.131392

1 -1.540526 1.353638 -1.131392

1 1.516849 1.443877 -1.051641

1 -0.069590 -0.940647 -2.040868

**N-methylazepane N-oxide**

The number of imaginary frequencies: 0

APFD/aug-cc-pVDZ

Enthalpy = -405.357516692

6 -0.608566 1.775840 0.774611

6 -0.608566 1.775840 -0.774611

6 0.569551 1.031910 -1.406300

6 0.569551 1.031910 1.406300

6 0.569551 -0.490908 -1.264864

6 0.569551 -0.490908 1.264864

1 -0.572153 2.815685 1.142885

1 -0.572153 2.815685 -1.142885

1 0.584187 1.234582 -2.490714

1 1.519222 1.442856 1.026234

1 1.594027 -0.887282 -1.366734

1 -0.065050 -0.946299 2.036392

7 -0.042627 -1.047369 0.000000

8 -1.373301 -0.839667 -0.000000

6 0.200612 -2.520014 0.000000

1 1.273957 -2.766223 0.000000

1 -0.296638 -2.918428 0.890819

1 -0.296638 -2.918428 -0.890819

1 1.594027 -0.887282 1.366734

1 0.584187 1.234582 2.490714

1 -1.543225 1.325445 1.133449

1 -1.543225 1.325445 -1.133449

1 1.519222 1.442856 -1.026234

1 -0.065050 -0.946299 -2.036392

**N-methylazepane N-oxide**

The number of imaginary frequencies: 0

APFD/aug-cc-pVTZ

Enthalpy = -405.475774305

6 -0.608880 1.778496 0.773162

6 -0.608880 1.778496 -0.773162

6 0.564014 1.032637 -1.404527

6 0.564014 1.032637 1.404527

6 0.564014 -0.487644 -1.261500

6 0.564014 -0.487644 1.261500

1 -0.571183 2.809741 1.137832

1 -0.571183 2.809741 -1.137832

1 0.574914 1.233755 -2.480047

1 1.505771 1.438754 1.027194

1 1.582187 -0.875613 -1.357279

1 -0.058439 -0.939919 2.031967

7 -0.041192 -1.048597 0.000000

8 -1.378162 -0.860724 0.000000

6 0.221575 -2.514703 0.000000

1 1.290643 -2.737348 0.000000

1 -0.262589 -2.919327 0.884357

1 -0.262589 -2.919327 -0.884357

1 1.582187 -0.875613 1.357279

1 0.574914 1.233755 2.480047

1 -1.538770 1.337450 1.128002

1 -1.538770 1.337450 -1.128002

1 1.505771 1.438754 -1.027194

1 -0.058439 -0.939919 -2.031967

**N-methylazepine**

The number of imaginary frequencies: 0

B3LYP/6-31G**

Enthalpy = -326.859656161

6 -0.609487 -1.771782 0.676167

6 -0.609487 -1.771782 -0.676167

6 -0.609487 -0.579396 -1.518592

6 -0.609487 -0.579396 1.518592

6 -0.053329 0.600352 -1.193902

6 -0.053329 0.600352 1.193902

1 -0.689963 -2.728232 1.190057

1 -0.689963 -2.728232 -1.190057

1 -1.123176 -0.641556 -2.475142

1 -1.123176 -0.641556 2.475142

1 -0.151617 1.457795 -1.862488

1 -0.151617 1.457795 1.862488

7 0.681491 0.827495 0.000000

6 1.523504 2.013041 0.000000

1 0.950836 2.958234 0.000000

1 2.167430 2.002472 0.883203

1 2.167430 2.002472 -0.883203

**N-methylazepine**

The number of imaginary frequencies: 0

APFD/aug-cc-pVDZ

Enthalpy = -326.594812304

6 -0.634887 -1.747142 0.677373

6 -0.634887 -1.747142 -0.677373

6 -0.634887 -0.554397 -1.512618

6 -0.634887 -0.554397 1.512618

6 -0.050612 0.611857 -1.181001

6 -0.050612 0.611857 1.181001

1 -0.727264 -2.709068 1.193506

1 -0.727264 -2.709068 -1.193506

1 -1.168036 -0.600160 -2.466926

1 -1.168036 -0.600160 2.466926

1 -0.142786 1.489207 -1.836567

1 -0.142786 1.489207 1.836567

7 0.706137 0.791799 0.000000

6 1.580894 1.940897 0.000000

1 1.035579 2.909451 0.000000

1 2.228447 1.909404 0.887501

1 2.228447 1.909404 -0.887501

**N-methylazepine**

The number of imaginary frequencies: 0

APFD/zug-cc-pVTZ

Enthalpy = -326.682346079

6 -0.621889 -1.751999 0.672556

6 -0.621889 -1.751999 -0.672556

6 -0.621889 -0.563235 -1.506411

6 -0.621889 -0.563235 1.506411

6 -0.045794 0.598633 -1.181834

6 -0.045794 0.598633 1.181834

1 -0.705900 -2.706045 1.185584

1 -0.705900 -2.706045 -1.185584

1 -1.139456 -0.614618 -2.458628

1 -1.139456 -0.614618 2.458628

1 -0.127540 1.459628 -1.844287

1 -0.127540 1.459628 1.844287

7 0.693358 0.805277 0.000000

6 1.539113 1.974540 0.000000

1 0.970210 2.917998 0.000000

1 2.181132 1.959552 0.880610

1 2.181132 1.959552 -0.880610

**1-methyl-2,3,4,5-tetrahydro-1H-azepine**

The number of imaginary frequencies: 0

B3LYP/6-31G**

Enthalpy= -329.310375943

6 -1.660774 -0.814901 -0.590395

6 -2.050443 0.546084 0.020195

6 -0.847314 1.381365 0.510666

6 -0.607181 -1.584990 0.176633

6 0.455989 1.204551 -0.281741

6 0.652078 -1.179921 0.419891

1 -2.561653 -1.430564 -0.686480

1 -2.731133 0.391165 0.865819

1 -1.121086 2.442831 0.493684

1 -0.875705 -2.567305 0.554317

1 1.085612 2.088935 -0.123744

7 1.244816 0.060230 0.179915

6 2.629155 0.050140 -0.259049

1 3.103870 1.009002 -0.022126

1 3.175884 -0.739000 0.266142

1 2.744132 -0.126581 -1.342437

1 1.333690 -1.868786 0.918603

1 -1.313274 -0.652734 -1.619447

1 -2.618875 1.113319 -0.728478

1 -0.622567 1.139710 1.554890

1 0.258342 1.164435 -1.367346

**1-methyl-2,3,4,5-tetrahydro-1H-azepine**

The number of imaginary frequencies: 0

APFD/aug-cc-pVDZ

Enthalpy= -329.040184702

6 -1.633891 -0.805465 -0.607611

6 -2.037924 0.542103 0.004637

6 -0.846602 1.361647 0.525568

6 -0.611450 -1.578749 0.186986

6 0.452563 1.204956 -0.264153

6 0.648827 -1.172085 0.432424

1 -2.534883 -1.422368 -0.753229

1 -2.743157 0.379817 0.837815

1 -1.122150 2.429166 0.544853

1 -0.894314 -2.555576 0.587196

1 1.087822 2.089626 -0.087390

7 1.235104 0.059056 0.173200

6 2.605490 0.049902 -0.272368

1 3.090609 1.009164 -0.028756

1 3.159006 -0.751845 0.239068

1 2.709858 -0.114779 -1.365143

1 1.334187 -1.856290 0.947418

1 -1.238966 -0.631742 -1.624588

1 -2.593302 1.122752 -0.753707

1 -0.621824 1.085202 1.569104

1 0.259301 1.189624 -1.357934

**1-methyl-2,3,4,5-tetrahydro-1H-azepine**

The number of imaginary frequencies: 0

APFD/aug-cc-pVTZ

Enthalpy= -329.132074646

6 -1.631766 -0.804752 -0.602114

6 -2.033773 0.543621 0.003461

6 -0.843468 1.355649 0.528558

6 -0.607016 -1.571976 0.188732

6 0.448144 1.201462 -0.268138

6 0.647505 -1.169017 0.426087

1 -2.525747 -1.418677 -0.735123

1 -2.737962 0.385343 0.825777

1 -1.115489 2.414309 0.558836

1 -0.884640 -2.543172 0.580897

1 1.079520 2.078817 -0.097631

7 1.229899 0.055877 0.162681

6 2.603797 0.053700 -0.264817

1 3.076679 1.007055 -0.015750

1 3.148743 -0.740833 0.247504

1 2.717513 -0.108027 -1.347490

1 1.328222 -1.849614 0.932174

1 -1.246927 -0.637754 -1.613791

1 -2.575874 1.121057 -0.753304

1 -0.620765 1.067129 1.559085

1 0.246905 1.181100 -1.350566

**1-methyl-2,3,6,7-tetrahydro-1H-azepine**

The number of imaginary frequencies: 0

B3LYP/6-31G**

Enthalpy= -329.298190209

6 -0.117334 -1.817960 0.668012

6 -0.117334 -1.817960 -0.668012

6 -0.795123 -0.702640 -1.426087

6 -0.795123 -0.702640 1.426087

6 -0.117334 0.685480 -1.243606

6 -0.117334 0.685480 1.243606

1 -0.837360 -0.923310 -2.497320

1 -0.887863 1.472632 -1.374158

7 0.631652 0.846287 -0.000000

6 1.381910 2.091312 -0.000000

1 0.743786 2.997527 -0.000000

1 2.024232 2.137182 0.885874

1 2.024232 2.137182 -0.885874

1 -0.887863 1.472632 1.374158

1 0.382770 -2.607309 1.224157

1 0.382770 -2.607309 -1.224157

1 -1.839519 -0.633339 -1.093264

1 0.608080 0.830160 -2.052764

1 -0.837360 -0.923310 2.497320

1 0.608080 0.830160 2.052764

1 -1.839519 -0.633339 1.093264

**1-methyl-2,3,6,7-tetrahydro-1H-azepine**

The number of imaginary frequencies: 0

APFD/aug-cc-pVDZ

Enthalpy= -329.028619850

6 -0.117214 -1.802277 0.669165

6 -0.117214 -1.802277 -0.669165

6 -0.803991 -0.690553 -1.413492

6 -0.803991 -0.690553 1.413492

6 -0.117214 0.684780 -1.232261

6 -0.117214 0.684780 1.232261

1 -0.871377 -0.908076 -2.490298

1 -0.882342 1.488555 -1.351028

7 0.635103 0.828533 -0.000000

6 1.394302 2.055214 -0.000000

1 0.760324 2.972453 -0.000000

1 2.040767 2.097090 0.890427

1 2.040767 2.097090 -0.890427

1 -0.882342 1.488555 1.351028

1 0.395616 -2.590891 1.227973

1 0.395616 -2.590891 -1.227973

1 -1.846947 -0.621006 -1.054953

1 0.608872 0.830894 -2.049088

1 -0.871377 -0.908076 2.490298

1 0.608872 0.830894 2.049088

1 -1.846947 -0.621006 1.054953

**1-methyl-2,3,6,7-tetrahydro-1H-azepine**

The number of imaginary frequencies: 0

APFD/aug-cc-pVTZ

Enthalpy= -329.120153118

6 -0.115721 -1.800088 0.664583

6 -0.115721 -1.800088 -0.664583

6 -0.799039 -0.692754 -1.410560

6 -0.799039 -0.692754 1.410560

6 -0.115721 0.681737 -1.231163

6 -0.115721 0.681737 1.231163

1 -0.859878 -0.911409 -2.478286

1 -0.878052 1.475381 -1.347917

7 0.631095 0.832311 0.000000

6 1.384059 2.060551 0.000000

1 0.747002 2.964152 0.000000

1 2.024250 2.104781 0.883534

1 2.024250 2.104781 -0.883534

1 -0.878052 1.475381 1.347917

1 0.392059 -2.582268 1.218953

1 0.392059 -2.582268 -1.218953

1 -1.834151 -0.623112 -1.058122

1 0.604144 0.826434 -2.041318

1 -0.859878 -0.911409 2.478286

1 0.604144 0.826434 2.041318

1 -1.834151 -0.623112 1.058122

**1-methyl-2,3,4,7-tetrahydro-1H-azepine**

The number of imaginary frequencies: 0

B3LYP/6-31G**

Enthalpy= -329.304274524

6 1.833298 0.796941 -0.311605

6 1.748059 -0.695644 -0.533662

6 0.866298 -1.402891 0.509448

6 0.867290 1.577518 0.179704

6 -0.642176 -1.269839 0.261397

6 -0.555417 1.179060 0.506237

1 2.762488 -1.109411 -0.480336

1 1.104056 -2.473073 0.539757

1 -1.178569 -1.523506 1.199666

7 -1.050247 0.032799 -0.259575

6 -2.492718 0.078889 -0.449834

1 -3.068776 0.001519 0.493455

1 -2.776431 1.012719 -0.945075

1 -2.803289 -0.750401 -1.094210

1 -0.677565 1.037558 1.601189

1 2.790792 1.261848 -0.542959

1 1.384903 -0.914766 -1.547568

1 1.121763 -1.005611 1.498725

1 -0.952149 -2.016047 -0.482058

1 1.091063 2.630467 0.347851

1 -1.194361 2.034910 0.258478

**1-methyl-2,3,4,7-tetrahydro-1H-azepine**

The number of imaginary frequencies: 0

APFD/aug-cc-pVDZ

Enthalpy= -329.034597535

6 1.823517 0.786564 -0.317485

6 1.719667 -0.698792 -0.545825

6 0.860971 -1.392823 0.515894

6 0.864605 1.570924 0.186838

6 -0.641458 -1.258127 0.275438

6 -0.551759 1.169588 0.508676

1 2.736338 -1.125749 -0.529289

1 1.101803 -2.467998 0.565011

1 -1.185867 -1.494335 1.221682

7 -1.034729 0.032347 -0.259505

6 -2.464660 0.082312 -0.461276

1 -3.049428 0.018332 0.485324

1 -2.744602 1.015251 -0.973467

1 -2.778119 -0.758139 -1.099903

1 -0.676766 1.018477 1.608760

1 2.788360 1.250660 -0.552126

1 1.318455 -0.906143 -1.555104

1 1.129319 -0.974515 1.501246

1 -0.961500 -2.015588 -0.461592

1 1.098782 2.626912 0.365175

1 -1.198967 2.028534 0.267271

**1-methyl-2,3,4,7-tetrahydro-1H-azepine**

The number of imaginary frequencies: 0

APFD/aug-cc-pVTZ

Enthalpy= -329.126037128

6 1.819980 0.784657 -0.316078

6 1.720924 -0.698150 -0.537649

6 0.855284 -1.387898 0.516836

6 0.866581 1.564342 0.182039

6 -0.641341 -1.259149 0.260372

6 -0.545663 1.165131 0.508275

1 2.730083 -1.119362 -0.509365

1 1.097178 -2.453102 0.572715

1 -1.188598 -1.502230 1.191995

7 -1.036478 0.034366 -0.258923

6 -2.465314 0.086886 -0.452967

1 -3.036045 0.015298 0.490714

1 -2.744277 1.017512 -0.949909

1 -2.779700 -0.741011 -1.091816

1 -0.662443 1.006823 1.598721

1 2.775630 1.244882 -0.553316

1 1.332289 -0.908651 -1.540997

1 1.107009 -0.965185 1.493915

1 -0.947250 -2.003837 -0.481906

1 1.095623 2.613578 0.350556

1 -1.186864 2.019811 0.276185

**N-methylazepane**

The number of imaginary frequencies: 0

B3LYP/6-31G**

Enthalpy = -330.534354811

6 -0.123724 -1.768559 0.779985

6 -0.123724 -1.768559 -0.779985

6 -0.850466 -0.578342 -1.425059

6 -0.850466 -0.578342 1.425059

6 -0.123724 0.768839 -1.236262

6 -0.123724 0.768839 1.236262

1 -0.594277 -2.690315 1.142355

1 -0.594277 -2.690315 -1.142355

1 -0.951502 -0.757329 -2.502387

1 -1.875529 -0.518078 1.044370

1 -0.851133 1.600321 -1.335329

1 0.595416 0.893796 2.055068

7 0.652331 0.862959 -0.000000

6 1.492463 2.051620 -0.000000

1 0.917986 2.998952 -0.000000

1 2.136105 2.052321 0.885746

1 2.136105 2.052321 -0.885746

1 -0.851133 1.600321 1.335329

1 -0.951502 -0.757329 2.502387

1 0.908855 -1.787038 1.144541

1 0.908855 -1.787038 -1.144541

1 -1.875529 -0.518078 -1.044370

1 0.595416 0.893796 -2.055068

**N-methylazepane**

The number of imaginary frequencies: 0

APFD/aug-cc-pVDZ

Enthalpy = -330.262189970

6 -0.124173 -1.749599 0.776635

6 -0.124173 -1.749599 -0.776635

6 -0.857783 -0.567553 -1.412621

6 -0.857783 -0.567553 1.412621

6 -0.124173 0.767764 -1.224833

6 -0.124173 0.767764 1.224833

1 -0.586118 -2.681431 1.144048

1 -0.586118 -2.681431 -1.144048

1 -0.976436 -0.744531 -2.494881

1 -1.884294 -0.506792 1.016680

1 -0.844754 1.615359 -1.311636

1 0.595917 0.894194 2.050876

7 0.653599 0.843264 -0.000000

6 1.503736 2.011210 -0.000000

1 0.935913 2.970142 -0.000000

1 2.151151 2.006138 0.890306

1 2.151151 2.006138 -0.890306

1 -0.844754 1.615359 1.311636

1 -0.976436 -0.744531 2.494881

1 0.914548 -1.756732 1.145893

1 0.914548 -1.756732 -1.145893

1 -1.884294 -0.506792 -1.016680

1 0.595917 0.894194 -2.050876

**N-methylazepane**

The number of imaginary frequencies: 0

APFD/aug-cc-pVTZ

Enthalpy = -330.355334937

6 -0.121742 -1.749206 0.775353

6 -0.121742 -1.749206 -0.775353

6 -0.853859 -0.569530 -1.410241

6 -0.853859 -0.569530 1.410241

6 -0.121742 0.763356 -1.224235

6 -0.121742 0.763356 1.224235

1 -0.578486 -2.673973 1.139279

1 -0.578486 -2.673973 -1.139279

1 -0.970148 -0.746248 -2.483503

1 -1.870596 -0.507585 1.015801

1 -0.838599 1.601933 -1.310440

1 0.593033 0.887046 2.043155

7 0.650728 0.846893 0.000000

6 1.490725 2.019665 0.000000

1 0.915859 2.963631 0.000000

1 2.132120 2.019859 0.883400

1 2.132120 2.019859 -0.883400

1 -0.838599 1.601933 1.310440

1 -0.970148 -0.746248 2.483503

1 0.909085 -1.753690 1.138273

1 0.909085 -1.753690 -1.138273

1 -1.870596 -0.507585 -1.015801

1 0.593033 0.887046 -2.043155

**N-Methylpyrrole N-oxide**

The number of imaginary frequencies: 0

B3LYP/6-31G**

Enthalpy= -324.573601410

6 1.618482 0.737646 0.097116

6 1.618414 -0.737501 0.098435

6 0.355178 -1.157788 -0.009827

6 0.355196 1.158013 -0.012549

1 2.501313 1.363007 0.116943

1 2.501241 -1.362844 0.118411

1 -0.094951 -2.133540 -0.104607

1 -0.095166 2.133563 -0.108432

7 -0.562707 -0.000157 -0.096040

8 -1.231366 -0.001924 -1.299503

6 -1.552719 0.001445 1.033634

1 -2.170095 0.888862 0.898347

1 -2.170107 -0.886355 0.900952

1 -1.049673 0.002897 2.005836

**N-Methylpyrrole N-oxide**

The number of imaginary frequencies: 0

APFD/aug-cc-pVDZ

Enthalpy= -324.322161700

6 1.610683 0.736027 0.095017

6 1.610665 -0.736086 0.095032

6 0.345214 -1.151806 -0.017876

6 0.345274 1.151814 -0.017496

1 2.496476 1.368927 0.116952

1 2.496422 -1.369018 0.117473

1 -0.112032 -2.131874 -0.113321

1 -0.111836 2.131884 -0.113512

7 -0.564700 0.000018 -0.102412

8 -1.230268 0.000086 -1.284835

6 -1.525150 -0.000050 1.037748

1 -2.149438 0.892362 0.915318

1 -2.149489 -0.892440 0.915342

1 -0.995171 -0.000046 2.002758

**N-Methylpyrrole N-oxide**

The number of imaginary frequencies: 0

APFD/aug-cc-pVTZ

Enthalpy= -324.417780070

6 1.602973 0.734263 0.088198

6 1.602915 -0.734116 0.089678

6 0.346661 -1.149278 -0.016996

6 0.346632 1.149492 -0.019776

1 2.481669 1.360937 0.117130

1 2.481615 -1.360728 0.119017

1 -0.106248 -2.121296 -0.109862

1 -0.106523 2.121310 -0.113620

7 -0.561759 -0.000171 -0.104665

8 -1.240514 -0.001994 -1.282137

6 -1.510277 0.001515 1.044399

1 -2.129362 0.887896 0.934686

1 -2.129527 -0.885055 0.937175

1 -0.968622 0.002830 1.992206

**N-methyl-2-pyrroline N-oxide**

The number of imaginary frequencies: 0

B3LYP/6-31G**

Enthalpy= -325.813525020

6 1.724798 0.621573 0.102158

6 1.525707 -0.878176 0.092168

6 0.240457 -1.196333 0.009033

6 0.351491 1.129373 -0.362271

1 1.988975 0.981248 1.105913

1 -0.268072 -2.148829 -0.029761

1 0.299008 1.241941 -1.443708

7 -0.647327 -0.014763 -0.108996

8 -1.525491 -0.166833 -1.127449

6 -1.401523 0.197798 1.174408

1 -2.060329 1.048181 0.998616

1 -2.003484 -0.695379 1.337294

1 -0.727303 0.375710 2.019018

1 2.339449 -1.592086 0.147047

1 2.526514 0.944681 -0.570564

1 -0.005125 2.037122 0.125734

**N-methyl-2-pyrroline N-oxide**

The number of imaginary frequencies: 0

APFD/aug-cc-pVDZ

Enthalpy= -325.559554428

6 1.699228 0.626505 0.099662

6 1.519410 -0.869653 0.081635

6 0.234035 -1.192443 -0.005468

6 0.338910 1.105798 -0.400816

1 1.929546 0.986067 1.119459

1 -0.279209 -2.150390 -0.043246

1 0.302095 1.165155 -1.494631

7 -0.648950 -0.016217 -0.112443

8 -1.552804 -0.168406 -1.083635

6 -1.335260 0.208120 1.193122

1 -2.009696 1.058643 1.037583

1 -1.929268 -0.689577 1.398113

1 -0.614306 0.399022 2.003893

1 2.341219 -1.584606 0.139856

1 2.522242 0.966507 -0.548808

1 -0.035484 2.039994 0.035154

**N-methyl-2-pyrroline N-oxide**

The number of imaginary frequencies: 0

APFD/aug-cc-pVTZ

Enthalpy= -325.657265137

6 1.700621 0.619649 0.098995

6 1.511624 -0.871104 0.073950

6 0.234307 -1.188048 -0.011483

6 0.337682 1.114584 -0.374683

1 1.948166 0.967604 1.108245

1 -0.276017 -2.136720 -0.053793

1 0.296959 1.220866 -1.455370

7 -0.644978 -0.014008 -0.111152

8 -1.543340 -0.154901 -1.096392

6 -1.341990 0.189963 1.188782

1 -2.008656 1.037663 1.049725

1 -1.929672 -0.704090 1.379165

1 -0.626813 0.366110 1.995222

1 2.323617 -1.583048 0.131502

1 2.506973 0.954709 -0.557000

1 -0.026455 2.023903 0.098124

**N-methyl-3-pyrroline N-oxide**

The number of imaginary frequencies: 0

B3LYP/6-31G**

Enthalpy= -325.814117087

6 1.659112 0.667757 0.078666

6 1.659089 -0.667824 0.078611

6 0.306599 -1.205279 -0.282567

6 0.306694 1.205298 -0.282477

1 0.205106 -1.468262 -1.338934

1 0.204970 1.468429 -1.338757

7 -0.643806 0.000008 -0.112990

8 -1.660544 0.000082 -0.983480

6 -1.186601 -0.000046 1.292211

1 -1.811178 0.888082 1.377663

1 -1.811409 -0.888032 1.377394

1 -0.387746 -0.000248 2.040368

1 2.513539 -1.301454 0.291465

1 2.513650 1.301364 0.291211

1 -0.052479 2.039171 0.326025

1 -0.052812 -2.039200 0.325669

**N-methyl-3-pyrroline N-oxide**

The number of imaginary frequencies: 0

APFD/aug-cc-pVDZ

Enthalpy= -325.560494446

6 1.645691 0.668572 0.073567

6 1.645454 -0.668675 0.073390

6 0.296916 -1.192751 -0.296104

6 0.297074 1.193022 -0.295011

1 0.199026 -1.441229 -1.363506

1 0.199004 1.442315 -1.362187

7 -0.643142 0.000052 -0.113380

8 -1.670807 0.000594 -0.948348

6 -1.141950 -0.000658 1.295399

1 -1.768933 0.891751 1.398233

1 -1.769241 -0.892986 1.397129

1 -0.315842 -0.001240 2.023173

1 2.503806 -1.307034 0.291256

1 2.504150 1.306504 0.292150

1 -0.071332 2.040938 0.299241

1 -0.071305 -2.041200 0.297504

**N-methyl-3-pyrroline N-oxide**

The number of imaginary frequencies: 0

APFD/aug-cc-pVTZ

Enthalpy= -325.657786634

6 1.645070 0.663747 0.071136

6 1.644692 -0.663946 0.071139

6 0.296218 -1.193971 -0.276184

6 0.296470 1.194302 -0.274766

1 0.197894 -1.476386 -1.325430

1 0.197546 1.478063 -1.323537

7 -0.637502 0.000082 -0.112470

8 -1.651287 0.000779 -0.974962

6 -1.162751 -0.000816 1.282626

1 -1.783542 0.885363 1.380999

1 -1.784105 -0.886783 1.379457

1 -0.349976 -0.001704 2.011704

1 2.500305 -1.297236 0.266788

1 2.500777 1.296522 0.267837

1 -0.061962 2.016985 0.344062

1 -0.062319 -2.017520 0.341402

**N-methylpyrrolidine N-oxide**

The number of imaginary frequencies: 0

B3LYP/6-31G**

Enthalpy= -327.050367463

6 1.603663 0.781996 0.090072

6 1.603543 -0.782088 0.090005

6 0.207002 -1.179916 -0.397304

6 0.206897 1.180170 -0.396436

1 1.804230 1.168334 1.093772

1 1.803291 -1.168510 1.093835

1 0.140440 -1.273784 -1.482064

1 0.140075 1.275167 -1.481082

7 -0.712799 0.000065 -0.102554

8 -1.821085 0.000278 -0.863836

6 -1.111849 -0.000465 1.347292

1 -1.725599 0.887101 1.494613

1 -1.724655 -0.888716 1.494402

1 -0.251770 -0.000117 2.025907

1 -0.218069 -2.074408 0.059459

1 2.376478 -1.189930 -0.566460

1 2.376240 1.189714 -0.566904

1 -0.217920 2.074288 0.061322

**N-methylpyrrolidine N-oxide**

The number of imaginary frequencies: 0

APFD/aug-cc-pVDZ

Enthalpy= -326.793508853

6 1.589304 0.777796 0.086399

6 1.589429 -0.777691 0.086687

6 0.199576 -1.167377 -0.402643

6 0.199576 1.167034 -0.403601

1 1.787755 1.170543 1.095653

1 1.788462 -1.170027 1.095978

1 0.130996 -1.252875 -1.494831

1 0.131267 1.251498 -1.495891

7 -0.711118 -0.000052 -0.102188

8 -1.817101 -0.000383 -0.838490

6 -1.078865 0.000575 1.343453

1 -1.693654 0.893068 1.502963

1 -1.693458 -0.891899 1.503806

1 -0.199700 0.000991 2.007486

1 -0.233713 -2.070548 0.044126

1 2.368126 -1.192045 -0.570014

1 2.368265 1.192085 -0.570035

1 -0.233833 2.070615 0.042220

**N-methylpyrrolidine N-oxide**

The number of imaginary frequencies: 0

APFD/aug-cc-pVTZ

Enthalpy= -326.893346480

6 1.583689 0.776507 0.082222

6 1.583959 -0.776276 0.082592

6 0.195638 -1.166252 -0.404176

6 0.195629 1.165640 -0.405808

1 1.779868 1.164568 1.083134

1 1.781225 -1.163802 1.083488

1 0.131965 -1.257010 -1.487102

1 0.132421 1.254540 -1.488921

7 -0.707597 -0.000090 -0.099394

8 -1.821665 -0.000610 -0.832887

6 -1.063806 0.000937 1.345564

1 -1.668754 0.887275 1.514681

1 -1.668737 -0.885172 1.515927

1 -0.180650 0.001392 1.988105

1 -0.230929 -2.060249 0.045229

1 2.355238 -1.185419 -0.570063

1 2.355403 1.185689 -0.569891

1 -0.231199 2.060364 0.041897

**N-Methylpyrrole**

The number of imaginary frequencies: 0

B3LYP/6-31G**

Enthalpy= -249.488688331

6 1.491542 0.711677 0.016064

6 1.491605 -0.711621 0.016055

6 0.175200 -1.119627 -0.014329

6 0.175126 1.119618 -0.014423

1 2.354859 1.362200 0.022539

1 2.354977 -1.362074 0.022380

1 -0.261470 -2.107877 -0.023476

1 -0.261582 2.107854 -0.023313

7 -0.625375 -0.000019 -0.039882

6 -2.073948 -0.000003 0.026688

1 -2.465245 0.883791 -0.482987

1 -2.464783 -0.886868 -0.477929

1 -2.436274 0.002840 1.061627

**N-Methylpyrrole**

The number of imaginary frequencies: 0

APFD/aug-cc-pVDZ

Enthalpy= -249.288183211

6 1.489036 0.710705 0.015404

6 1.488925 -0.710786 0.015503

6 0.170288 -1.116176 -0.014162

6 0.170438 1.116181 -0.014125

1 2.356556 1.366817 0.022926

1 2.356410 -1.366932 0.022099

1 -0.271895 -2.109242 -0.022631

1 -0.271591 2.109316 -0.023091

7 -0.623432 0.000002 -0.038810

6 -2.062873 0.000080 0.026085

1 -2.457582 0.887737 -0.486729

1 -2.457117 -0.891483 -0.480206

1 -2.425637 0.003751 1.067072

**N-Methylpyrrole**

The number of imaginary frequencies: 0

APFD/aug-cc-pVTZ

Enthalpy= -249.356058342

6 1.482743 0.707757 0.014014

6 1.482795 -0.707706 0.014044

6 0.170933 -1.111677 -0.012696

6 0.170896 1.111651 -0.012853

1 2.343550 1.357411 0.020707

1 2.343654 -1.357296 0.020302

1 -0.266361 -2.096940 -0.021290

1 -0.266420 2.096907 -0.021004

7 -0.620071 -0.000019 -0.035081

6 -2.057917 0.000016 0.023689

1 -2.446181 0.881288 -0.485809

1 -2.445715 -0.884811 -0.479914

1 -2.418727 0.003332 1.055381

**N-methyl-2-pyrroline**

The number of imaginary frequencies: 0

B3LYP/6-31G**

Enthalpy= -250.680844113

6 -1.580164 -0.617095 -0.028381

6 -1.374505 0.875399 0.139141

6 -0.078183 1.152311 -0.057952

6 -0.126568 -1.141239 0.048724

1 -2.221183 -1.073692 0.733501

1 0.400225 2.125893 -0.075806

1 0.069547 -1.998032 -0.603363

7 0.703034 0.017737 -0.334592

6 2.082715 -0.009804 0.113135

1 2.605033 -0.853831 -0.348712

1 2.590996 0.907491 -0.198498

1 2.181199 -0.106315 1.209794

1 -2.167178 1.601922 0.257257

1 -2.031563 -0.845992 -1.005419

1 0.111921 -1.439029 1.085388

**N-methyl-2-pyrroline**

The number of imaginary frequencies: 0

APFD/aug-cc-pVDZ

Enthalpy= -250.474897760

6 -1.568707 -0.618424 -0.041824

6 -1.373422 0.867641 0.143669

6 -0.075428 1.146756 -0.055101

6 -0.122719 -1.129168 0.063129

1 -2.232117 -1.089974 0.700208

1 0.406399 2.126023 -0.067721

1 0.090651 -2.007614 -0.564609

7 0.697385 0.016705 -0.335908

6 2.068680 -0.009515 0.106068

1 2.586282 -0.873344 -0.338055

1 2.588705 0.899868 -0.229335

1 2.166937 -0.082383 1.211143

1 -2.171183 1.598095 0.266286

1 -1.996673 -0.835249 -1.040073

1 0.108883 -1.396092 1.117864

**N-methyl-2-pyrroline**

The number of imaginary frequencies: 0

APFD/aug-cc-pVTZ

Enthalpy= -250.545365644

6 -1.566783 -0.615622 -0.040830

6 -1.366675 0.867463 0.140258

6 -0.075913 1.142950 -0.054270

6 -0.124034 -1.129298 0.062163

1 -2.223076 -1.076798 0.699968

1 0.401825 2.114080 -0.065610

1 0.085885 -1.996953 -0.565256

7 0.694942 0.016811 -0.330911

6 2.066120 -0.009751 0.104384

1 2.579776 -0.862289 -0.343329

1 2.577202 0.896919 -0.221821

1 2.162310 -0.089243 1.199546

1 -2.156465 1.592280 0.264383

1 -1.994180 -0.832908 -1.028568

1 0.105839 -1.397218 1.106833

**N-methyl-3-pyrroline**

The number of imaginary frequencies: 0

B3LYP/6-31G**

Enthalpy= -250.672907553

6 1.528447 -0.666301 -0.042760

6 1.528344 0.666445 -0.042837

6 0.117095 1.191194 0.005632

6 0.117228 -1.191234 0.005572

1 -0.027772 1.988358 0.749633

1 -0.027693 -1.988552 0.749369

7 -0.668125 -0.000121 0.352374

6 -2.037816 -0.000011 -0.113710

1 -2.562067 -0.882936 0.267128

1 -2.561699 0.883243 0.266871

1 -2.131388 -0.000136 -1.218156

1 2.398683 1.311000 -0.099085

1 2.398909 -1.310726 -0.098700

1 -0.164736 -1.623648 -0.977650

1 -0.165147 1.623686 -0.977416

**N-methyl-3-pyrroline**

The number of imaginary frequencies: 0

APFD/aug-cc-pVDZ

Enthalpy= -250.466571606

6 1.521902 -0.667274 -0.035307

6 1.521939 0.667277 -0.035246

6 0.112892 1.181910 -0.002914

6 0.112879 -1.181905 -0.002892

1 -0.043965 1.994056 0.731027

1 -0.043930 -1.994057 0.731061

7 -0.661297 -0.000001 0.350387

6 -2.024341 0.000009 -0.104413

1 -2.550089 -0.887833 0.280100

1 -2.550051 0.887912 0.280008

1 -2.122706 -0.000046 -1.215231

1 2.398626 1.315138 -0.086399

1 2.398551 -1.315206 -0.086377

1 -0.164501 -1.601313 -1.001119

1 -0.164484 1.601249 -1.001150

**N-methyl-3-pyrroline**

The number of imaginary frequencies: 0

APFD/aug-cc-pVTZ

Enthalpy= -250.536929435

6 1.518221 -0.662893 -0.036200

6 1.518247 0.662871 -0.036217

6 0.114080 1.180486 -0.001852

6 0.114036 -1.180456 -0.001870

1 -0.039768 1.983059 0.728936

1 -0.039806 -1.983093 0.728869

7 -0.660293 -0.000017 0.348406

6 -2.022204 0.000017 -0.104755

1 -2.543078 -0.881206 0.275184

1 -2.543166 0.881056 0.275492

1 -2.113460 0.000194 -1.206318

1 2.388019 1.304497 -0.087063

1 2.387994 -1.304525 -0.086916

1 -0.164467 -1.595390 -0.990852

1 -0.164497 1.595380 -0.990808

**N-methylpyrrolidine**

The number of imaginary frequencies: 0

B3LYP/6-31G**

Enthalpy= -251.909652936

6 1.497577 -0.778513 -0.057205

6 1.497372 0.778557 -0.058524

6 0.020846 1.157260 0.162650

6 0.020546 -1.157422 0.161652

1 1.855186 -1.162445 -1.016488

1 1.852351 1.160794 -1.019470

1 -0.174773 1.320605 1.242580

1 -0.176284 -1.322363 1.241118

7 -0.713585 0.000201 -0.346134

6 -2.126797 0.000069 -0.023954

1 -2.324282 -0.001009 1.066758

1 -2.607763 0.885506 -0.452595

1 -2.607865 -0.884463 -0.454333

1 -0.279500 2.069013 -0.365571

1 2.140735 1.200005 0.718830

1 2.139154 -1.198258 0.722541

1 -0.279129 -2.068498 -0.368147

**N-methylpyrrolidine**

The number of imaginary frequencies: 0

APFD/aug-cc-pVDZ

Enthalpy= -251.695112499

6 1.244938 -0.772727 -0.398674

6 1.244436 0.773124 -0.398851

6 0.070332 1.139457 0.542006

6 0.070461 -1.139704 0.541547

1 1.078591 -1.168279 -1.412898

1 1.076987 1.168300 -1.413042

1 0.444189 1.283586 1.570522

1 0.443966 -1.284497 1.570097

7 -0.842947 -0.000173 0.548978

6 -1.734764 -0.000001 -0.598183

1 -2.384292 -0.887405 -0.557362

1 -2.384301 0.887383 -0.557085

1 -1.224334 0.000150 -1.586110

1 -0.457805 2.060597 0.254965

1 2.196103 1.199785 -0.046899

1 2.196639 -1.198606 -0.045870

1 -0.457536 -2.060693 0.253766

**N-methylpyrrolidine**

The number of imaginary frequencies: 0

APFD/aug-cc-pVTZ

Enthalpy= -251.767456643

6 1.242315 -0.771295 -0.397569

6 1.241516 0.771955 -0.397780

6 0.069801 1.139314 0.541076

6 0.070235 -1.139596 0.540488

1 1.077140 -1.162833 -1.403038

1 1.074833 1.162985 -1.403207

1 0.440998 1.284660 1.560273

1 0.441155 -1.285562 1.559710

7 -0.839682 -0.000299 0.547297

6 -1.732126 -0.000122 -0.597051

1 -2.374768 -0.881005 -0.556023

1 -2.374482 0.880962 -0.555981

1 -1.220270 -0.000179 -1.573315

1 -0.454804 2.050376 0.250511

1 2.185336 1.192933 -0.047769

1 2.186243 -1.191140 -0.046466

1 -0.454047 -2.050634 0.249240

**7-methyl-7-azanorcaradiene**

The number of imaginary frequencies: 0

B3LYP/6-31G**

Enthalpy=-326.847852849

6 -0.436335 -0.772082 0.538090

6 -0.436471 0.772316 0.537614

1 0.915223 -2.506512 0.354302

6 0.825961 -1.437596 0.179389

6 0.826126 1.437617 0.179439

6 1.866633 0.726845 -0.309153

6 1.866540 -0.726960 -0.309293

1 0.915317 2.506552 0.354254

1 2.781998 1.236432 -0.598102

1 2.781823 -1.236652 -0.598315

7 -1.129485 -0.000070 -0.496887

6 -2.583903 -0.000008 -0.389471

1 -2.978859 0.887802 -0.893875

1 -2.978971 -0.887530 -0.894316

1 -2.957761 -0.000254 0.649659

1 -1.071784 -1.269090 1.272531

1 -1.071898 1.268947 1.272386

**7-methyl-7-azanorcaradiene**

The number of imaginary frequencies: 0

APFD/aug-cc-pVDZ

Enthalpy=-326.587401162

6 -0.440908 -0.771702 0.540513

6 -0.441103 0.771773 0.540032

1 0.915933 -2.507708 0.378060

6 0.818491 -1.435448 0.187430

6 0.818462 1.435488 0.187555

6 1.854984 0.725602 -0.315009

6 1.854986 -0.725524 -0.315176

1 0.915700 2.507750 0.378255

1 2.774525 1.239771 -0.607674

1 2.774473 -1.239686 -0.608029

7 -1.113813 -0.000122 -0.493746

6 -2.558149 0.000035 -0.400311

1 -2.951795 0.891930 -0.911486

1 -2.951887 -0.892435 -0.910438

1 -2.943607 0.000631 0.641665

1 -1.088457 -1.270433 1.272807

1 -1.088778 1.269698 1.272864

**7-methyl-7-azanorcaradiene**

The number of imaginary frequencies: 0

APFD/aug-cc-pVTZ

Enthalpy=-326.673466629

6 -0.436903 -0.768745 0.535988

6 -0.437204 0.768606 0.535592

1 0.913056 -2.492779 0.369844

6 0.818727 -1.428362 0.184642

6 0.818439 1.428441 0.184832

6 1.849643 0.723140 -0.312072

6 1.849784 -0.722830 -0.312262

1 0.912374 2.492856 0.370199

1 2.762042 1.233209 -0.600944

1 2.762210 -1.232713 -0.601385

7 -1.114056 -0.000207 -0.491731

6 -2.556809 0.000055 -0.393940

1 -2.948535 0.885330 -0.898164

1 -2.948769 -0.885248 -0.897951

1 -2.928430 0.000200 0.642626

1 -1.079516 -1.263954 1.260498

1 -1.080095 1.262722 1.260705

**7-methyl-7-azanorcaradiene N-oxide**

The number of imaginary frequencies: 0

B3LYP/6-31G**

Enthalpy=-401.989673111

6 0.254634 -0.764735 -0.841923

6 0.252136 0.771185 -0.840037

1 -1.028447 -2.509205 -0.492686

6 -0.945139 -1.435976 -0.356914

6 -0.946719 1.438057 -0.350508

6 -1.905222 0.725971 0.283316

6 -1.905061 -0.727865 0.278690

1 -1.030156 2.512205 -0.478847

1 -2.764047 1.235150 0.711207

1 -2.764700 -1.239780 0.701733

7 1.068366 -0.002117 0.237861

6 2.529856 0.001846 0.018614

1 2.925968 0.891977 0.509904

1 2.929522 -0.891087 0.502142

1 2.778608 0.006678 -1.046313

1 0.878737 -1.280348 -1.566656

1 0.877476 1.288921 -1.561995

8 0.713946 -0.006324 1.488632

**7-methyl-7-azanorcaradiene N-oxide**

The number of imaginary frequencies: 0

APFD/aug-cc-pVDZ

Enthalpy=-401.683840334

6 0.267783 -0.765912 -0.850829

6 0.266149 0.768828 -0.851379

1 -1.024147 -2.511770 -0.522626

6 -0.930445 -1.434678 -0.374622

6 -0.930346 1.435990 -0.371472

6 -1.873434 0.725588 0.290486

6 -1.874281 -0.725756 0.287379

1 -1.023187 2.513828 -0.514689

1 -2.730252 1.239817 0.732943

1 -2.732553 -1.240742 0.726194

7 1.044658 -0.001765 0.246063

6 2.499037 0.001073 0.057002

1 2.886681 0.896917 0.558671

1 2.889451 -0.896205 0.554123

1 2.772801 0.004022 -1.007715

1 0.911605 -1.285576 -1.565411

1 0.911128 1.288788 -1.564505

8 0.659887 -0.003440 1.470147

**7-methyl-7-azanorcaradiene N-oxide**

The number of imaginary frequencies: 0

APFD/aug-cc-pVTZ

Enthalpy=-401.795253802

6 0.263928 -0.763667 -0.830803

6 0.262819 0.766694 -0.829889

1 -1.020975 -2.497238 -0.498128

6 -0.934220 -1.427234 -0.358865

6 -0.934720 1.428339 -0.355848

6 -1.888396 0.722350 0.276093

6 -1.888491 -0.722963 0.273851

1 -1.021181 2.498858 -0.491328

1 -2.745236 1.233196 0.700001

1 -2.745925 -1.234922 0.695253

7 1.051552 -0.001200 0.238865

6 2.499999 0.000973 0.024261

1 2.896081 0.890650 0.510218

1 2.898170 -0.888494 0.509050

1 2.746718 0.001794 -1.038041

1 0.897166 -1.279772 -1.542379

1 0.897013 1.283080 -1.540378

8 0.693974 -0.003214 1.478860

**Benzene**

The number of imaginary frequencies: 0

B3LYP/6-31G**

Enthalpy= -232.258203982

6 0.814686 -1.134019 -0.000280

6 -0.574873 -1.272443 0.000166

1 2.470537 0.246283 0.000304

6 1.389539 0.138440 0.000083

6 -1.389553 -0.138436 -0.000008

6 -0.814646 1.134003 -0.000131

6 0.574828 1.272409 0.000062

1 -2.470548 -0.246213 0.000090

1 -1.448104 2.016576 -0.000143

1 1.021853 2.262547 0.000193

1 1.448267 -2.016423 -0.000160

1 -1.021898 -2.262492 0.000362

**Benzene**

The number of imaginary frequencies: 0

APFD/aug-cc-pVDZ

Enthalpy= -232.065334419

6 1.311421 -0.479269 0.000555

6 0.240609 -1.375214 -0.000334

1 1.909110 1.597376 -0.000326

6 1.070691 0.896171 -0.000158

6 -1.070981 -0.895852 0.000030

6 -1.311271 0.479172 0.000275

6 -0.240382 1.375153 -0.000145

1 -1.908984 -1.597560 -0.000090

1 -2.337620 0.855130 0.000015

1 -0.429291 2.451781 -0.000324

1 2.337521 -0.855853 -0.000059

1 0.428743 -2.451840 -0.000558

**Benzene**

The number of imaginary frequencies: 0

APFD/aug-cc-pVTZ

Enthalpy= -232.124979909

6 0.746072 -1.171792 -0.000259

6 -0.641901 -1.231916 0.000215

1 2.471100 0.107159 0.000252

6 1.387945 0.060138 0.000090

6 -1.387961 -0.060107 -0.000025

6 -0.746027 1.171770 -0.000111

6 0.641863 1.231872 0.000077

1 -2.471107 -0.107112 -0.000005

1 -1.327926 2.086560 -0.000240

1 1.142586 2.193496 0.000142

1 1.328037 -2.086456 -0.000386

1 -1.142632 -2.193439 0.000322

**Trimethylamine**

The number of imaginary frequencies: 0

B3LYP/6-31G**

Enthalpy= -169.795990862

6 0.955144 -0.570336 0.000000

1 1.975859 -0.187524 0.000000

1 0.766308 -1.179993 0.889170

1 0.766308 -1.179993 -0.889170

7 -0.000000 0.590677 -0.000000

8 -1.154917 0.229348 -0.000000

**Trimethylamine**

The number of imaginary frequencies: 0

APFD/aug-cc-pVDZ

Enthalpy= -169.665311125

6 0.946529 -0.566157 0.000000

1 1.974762 -0.185648 0.000000

1 0.754389 -1.180394 0.893556

1 0.754389 -1.180394 -0.893556

7 -0.000000 0.584434 0.000000

8 -1.145339 0.231542 -0.000000

**Trimethylamine**

The number of imaginary frequencies: 0

APFD/aug-cc-pVTZ

Enthalpy= -169.716346849

6 0.945294 -0.566816 -0.000000

1 1.963677 -0.186875 -0.000000

1 0.755055 -1.174879 0.886696

1 0.755055 -1.174879 -0.886696

7 -0.000000 0.579570 0.000000

8 -1.143194 0.235067 0.000000

**Propane**

The number of imaginary frequencies: 0

Total Energy (B3LYP/6-31G*)= -119.14424274

ZPE= -119.040122

Enthalpy= -119.034676

Gibb’s Free Energy= -119.065764

C 1.27719 -0.25986 0.00002

C 0. 0.5865 0.

H 2.1765 0.36717 0.00003

H 1.32187 -0.90744 0.8847

H 1.3219 -0.90745 -0.88465

C -1.27719 -0.25986 -0.00002

H -0.00001 1.24736 0.8776

H 0.00001 1.24735 -0.87762

H -2.1765 0.36717 -0.00004

H -1.32187 -0.90745 -0.88469

H -1.3219 -0.90744 0.88467

**Propane**

The number of imaginary frequencies: 0

Total Energy (B3LYP/6-31G**)= -119.15535976

ZPE= -119.051645

Enthalpy= -119.046182

Gibb’s Free Energy= -119.077296

C 1.27719 -0.25986 0.00002

C 0. 0.5865 0.

H 2.1765 0.36717 0.00003

H 1.32187 -0.90744 0.8847

H 1.3219 -0.90745 -0.88465

C -1.27719 -0.25986 -0.00002

H -0.00001 1.24736 0.8776

H 0.00001 1.24735 -0.87762

H -2.1765 0.36717 -0.00004

H -1.32187 -0.90745 -0.88469

H -1.3219 -0.90744 0.88467

**Propane**

The number of imaginary frequencies: 0

Total Energy (M06/6-311G+(d,p))= -119.06693725

ZPE= -118.964632

Enthalpy= -118.959147

Gibb’s Free Energy= -118.990276

C 1.27719 -0.25986 0.00002

C 0. 0.5865 0.

H 2.1765 0.36717 0.00003

H 1.32187 -0.90744 0.8847

H 1.3219 -0.90745 -0.88465

C -1.27719 -0.25986 -0.00002

H -0.00001 1.24736 0.8776

H 0.00001 1.24735 -0.87762

H -2.1765 0.36717 -0.00004

H -1.32187 -0.90745 -0.88469

H -1.3219 -0.90744 0.88467
