# Supplementary material for: Predicted Reversal in N-Methylazepine/N-Methyl-7-azanorcaradiene Equilibrium upon Formation of Their N-Oxides
Source: Molecules. 2020 Oct 16;25(20):4767. doi: 10.3390/molecules25204767 (PMC7594072; doi:10.3390/molecules25204767)
Supplement: Supplementary file 1 [file molecules-25-04767-s001.zip › Supplemental/Supplemental Information.pdf]

Supporting Information

Predicted Reversal in N-Methylazepine/N-Methyl-7-azanorcaradiene Equilibrium Upon Formation of Their N-Oxides

René Fournier[1], Alexa R. Green[2], Arthur Greenberg[2,*], Edward Lee-Ruff[1,*], Joel F. Liebman[3,*] and Anita Rágyanszki[4,*]

1. Department of Chemistry, York University, Keele Campus, Toronto ON MJ3 1P3 Canada
2. Department of Chemistry, University of New Hampshire, Durham, NH 03824 USA
3. Department of Chemistry and Biochemistry, University of Maryland Baltimore County, Baltimore, MD 21250 USA
4. Department of Physics and Astronomy, York University, Keele Campus, Toronto ON MJ3 1P3 Canada

*E-mail: art.greenberg@unh.edu
leeruff@yorku.ca
jliebman@umbc.edu
a.ragyanszki@utoronto.ca

Cartesian Coordinates (Å) and energies (Hartrees) for all structures calculated using Gaussian 09.

N-methylpyrrole
The number of imaginary frequencies: 0
Total Energy (B3LYP/6-31G*)= -249.47784543
ZPE= -249.367168
Enthalpy= -249.360642
Gibb's Free Energy= -249.396193

C	-0.31737	-1.01923	0.17362
C	1.00946	-1.0133	-0.07679
C	1.4598	0.44188	-0.08284
C	0.36886	1.19813	0.16441
N	-0.76109	0.33469	0.58538
H	-0.95522	-1.87361	0.08373
H	1.62623	-1.86947	-0.25416
H	2.45195	0.79863	-0.26524
H	0.32489	2.26274	0.06655
C	-2.04157	0.72846	-0.0198
H	-2.29179	1.72172	0.28958
H	-1.95658	0.69773	-1.08597
H	-2.80837	0.05254	0.29651

N-methylpyrrole
The number of imaginary frequencies: 0
Total Energy (B3LYP/6-31G**)= -249.48868841
ZPE= -249.37822
Enthalpy= -249.371698
Gibb's Free Energy= -249.407248

C	0.	-0.17376	1.09496
C	0.	-1.46657	0.6966
C	0.	-1.46657	-0.6966
C	0.	-0.17376	-1.09496
N	0.	0.60624	0.
H	0.	0.16939	2.10844
H	0.	-2.32739	1.33212
H	0.	-2.32739	-1.33212
H	0.	0.16939	-2.10844
C	0.	2.07624	0.
H	0.5044	2.43291	-0.87365
H	-1.00881	2.43291	0.
H	0.5044	2.43291	0.87365

N-methylpyrrole
The number of imaginary frequencies: 0
Total Energy (M06/6-311G+**)= -249.34878244
ZPE= -249.239616
Enthalpy= -249.232966
Gibb's Free Energy= -249.269338

C	-0.31737	-1.01923	0.17362
C	1.00946	-1.0133	-0.07679
C	1.4598	0.44188	-0.08284
C	0.36886	1.19813	0.16441
N	-0.76109	0.33469	0.58538
H	-0.95522	-1.87361	0.08373
H	1.62623	-1.86947	-0.25416
H	2.45195	0.79863	-0.26524
H	0.32489	2.26274	0.06655
C	-2.04157	0.72846	-0.0198
H	-2.29179	1.72172	0.28958
H	-1.95658	0.69773	-1.08597
H	-2.80837	0.05254	0.29651

N-Methylpyrrole N-oxide
The number of imaginary frequencies: 0
Total Energy (B3LYP/6-31G*)= -324.56298019
ZPE= -324.449454
Enthalpy= -324.442285
Gibb's Free Energy= -324.479007

C	-0.3294	-1.01791	0.1578
C	1.00468	-1.01444	-0.06029
C	1.45655	0.44566	-0.06634
C	0.35814	1.20372	0.14859
N	-0.75911	0.33403	0.57648
H	-0.97374	-1.86404	0.04035
H	1.62512	-1.87187	-0.21771
H	2.45247	0.80154	-0.22879
H	0.30409	2.26497	0.02323
C	-2.04454	0.72939	-0.01703
H	-2.29215	1.72185	0.29697
H	-1.96841	0.7014	-1.08395
H	-2.80872	0.05267	0.30389
O	-0.87602	0.37583	1.93081

N-Methylpyrrole N-oxide
The number of imaginary frequencies: 0
Total Energy (B3LYP/6-31G**)= -324.57360076
ZPE= -324.46035
Enthalpy= -324.453172
Gibb's Free Energy= -324.489906

C	-0.04298	0.35255	1.15778
C	-0.04298	1.62083	0.7378
C	-0.04298	1.62083	-0.7378
C	-0.04298	0.35255	-1.15778

N	-0.04755	-0.56912	0.
H	-0.10099	-0.10602	2.13324
H	-0.09948	2.50313	1.36302
H	-0.09948	2.50313	-1.36302
H	-0.10099	-0.10602	-2.13324
C	1.16596	-1.45463	0.
H	1.08767	-2.08133	-0.8887
H	2.08944	-0.86521	0.
H	1.08767	-2.08133	0.8887
O	-1.18692	-1.34192	0.

N-methylpyrrole N-oxide
The number of imaginary frequencies: 0
Total Energy (M06/6-311G+**)= -324.43163272
ZPE= -324.31929
Enthalpy= -324.312193
Gibb's Free Energy= -324.348742

C	-0.3294	-1.01791	0.1578
C	1.00468	-1.01444	-0.06029
C	1.45655	0.44566	-0.06634
C	0.35814	1.20372	0.14859
N	-0.75911	0.33403	0.57648
H	-0.97374	-1.86404	0.04035
H	1.62512	-1.87187	-0.21771
H	2.45247	0.80154	-0.22879
H	0.30409	2.26497	0.02323
C	-2.04454	0.72939	-0.01703
H	-2.29215	1.72185	0.29697
H	-1.96841	0.7014	-1.08395
H	-2.80872	0.05267	0.30389
O	-0.87602	0.37583	1.93081

N-methylazepine
The number of imaginary frequencies: 0
Total Energy (B3LYP/6-31G*)= -326.84608435
ZPE= -326.702253
Enthalpy= -326.693875
Gibb's Free Energy= -326.733659

C	0.93863	0.15438	-0.2526
C	2.30347	0.15457	-0.25898
C	3.19274	1.20123	0.04876
C	0.05199	1.2008	0.06344
C	2.91009	2.40177	0.60544
C	0.33949	2.40141	0.61745
H	0.47792	-0.76674	-0.54277
H	2.7617	-0.76642	-0.55344
H	4.22198	1.0244	-0.18427
H	-0.97933	1.02368	-0.15996
H	3.70896	3.11213	0.6512
H	-0.45911	3.11155	0.67068
N	1.62734	2.81624	1.17006
C	1.63404	2.44107	2.59136
H	1.63673	3.32544	3.19368
H	0.76149	1.86222	2.81157
H	2.50877	1.86246	2.8034

N-methylazepine
The number of imaginary frequencies: 0
Total Energy (B3LYP/6-31G**)= -326.85965616
ZPE= -326.716154
Enthalpy= -326.70778
Gibb's Free Energy= -326.747543

C	0.61021	1.77096	0.67637
C	0.61021	1.77096	-0.67637
C	0.61021	0.57868	-1.51904
C	0.61021	0.57868	1.51904
C	0.05418	-0.6014	-1.19368
C	0.05418	-0.6014	1.19368
H	0.69105	2.72773	1.19091
H	0.69105	2.72773	-1.19091
H	1.12518	0.64113	-2.47565
H	1.12518	0.64113	2.47565
H	0.1529	-1.46083	-1.86048
H	0.1529	-1.46083	1.86048
N	-0.68116	-0.8274	0.
C	-1.52746	-2.0092	0.
H	-0.95778	-2.95729	0.
H	-2.17143	-1.99535	0.88425
H	-2.17143	-1.99535	-0.88425

N-methylazepine
The number of imaginary frequencies: 0
Total Energy (M06/6-311G+**)= -326.67492642
ZPE= -326.532731
Enthalpy= -326.524386
Gibb's Free Energy= -326.563984

C	0.62245	1.75336	0.67222
C	0.62245	1.75336	-0.67222
C	0.62245	0.56474	-1.50646
C	0.62245	0.56474	1.50646
C	0.04331	-0.59604	-1.18419
C	0.04331	-0.59604	1.18419
H	0.70572	2.70987	1.18507
H	0.70572	2.70987	-1.18507
H	1.14071	0.61519	-2.46069
H	1.14071	0.61519	2.46069
H	0.11856	-1.45761	-1.85176
H	0.11856	-1.45761	1.85176

N	-0.69474	-0.80586	0.
C	-1.53524	-1.98153	0.
H	-0.95466	-2.92166	0.
H	-2.17953	-1.97387	0.88285
H	-2.17953	-1.97387	-0.88285

N-methylazepine N-oxide
The number of imaginary frequencies: 0
Total Energy (B3LYP/6-31G*)= -401.97429587
ZPE= -401.825754
Enthalpy= -401.817041
Gibb’s Free Energy= -401.857431

C	0.82605	0.55465	-0.65489
C	2.17148	0.54639	-0.76242
C	3.08766	1.44622	-0.20978
C	0.02032	1.46506	0.03537
C	2.88442	2.40908	0.72364
C	0.38096	2.42445	0.92372
H	0.31053	-0.22416	-1.17699
H	2.58798	-0.23815	-1.35901
H	4.09119	1.36074	-0.57104
H	-1.02882	1.39218	-0.16184
H	3.7184	3.0554	0.90153
H	-0.40623	3.08073	1.23117
N	1.69221	2.67973	1.54823
C	1.78348	1.84441	2.75439
H	2.70185	2.05143	3.26295
H	0.96013	2.06213	3.40215
H	1.75489	0.81166	2.476
O	1.72659	3.99895	1.87699

N-methylazepine N-oxide
The number of imaginary frequencies: 0
Total Energy (B3LYP/6-31G**)= -401.98779312
ZPE= -401.839695
Enthalpy= -401.830971
Gibb’s Free Energy= -401.871365

C	0.75349	1.83108	0.68076
C	0.75349	1.83108	-0.68076
C	0.75349	0.64759	-1.50595
C	0.75349	0.64759	1.50595
C	0.29957	-0.58124	-1.1975
C	0.29957	-0.58124	1.1975
H	0.87786	2.78202	1.19381
H	0.87786	2.78202	-1.19381
H	1.21424	0.74192	-2.48817
H	1.21424	0.74192	2.48817
H	0.42486	-1.46195	-1.81406
H	0.42486	-1.46195	1.81406

N	-0.50404	-0.94862	0.
C	-1.83882	-0.25012	0.
H	-2.35394	-0.60559	-0.89226
H	-2.35394	-0.60559	0.89226
H	-1.73292	0.83728	0.
O	-0.7138	-2.29727	0.

N-methylazepine N-oxide
The number of imaginary frequencies: 0
Total Energy (M06/6-311G+**)= -401.79814103
ZPE= -401.651151
Enthalpy= -401.642487
Gibb’s Free Energy= -401.682651

C	0.82605	0.55465	-0.65489
C	2.17148	0.54639	-0.76242
C	3.08766	1.44622	-0.20978
C	0.02032	1.46506	0.03537
C	2.88442	2.40908	0.72364
C	0.38096	2.42445	0.92372
H	0.31053	-0.22416	-1.17699
H	2.58798	-0.23815	-1.35901
H	4.09119	1.36074	-0.57104
H	-1.02882	1.39218	-0.16184
H	3.7184	3.0554	0.90153
H	-0.40623	3.08073	1.23117
N	1.69221	2.67973	1.54823
C	1.78348	1.84441	2.75439
H	2.70185	2.05143	3.26295
H	0.96013	2.06213	3.40215
H	1.75489	0.81166	2.476
O	1.72659	3.99895	1.87699

1,3-Cyclopentadiene
The number of imaginary frequencies: 0
Total Energy (B3LYP/6-31G*)= -194.10105812
ZPE= -194.008165
Enthalpy= -194.003078
Gibb’s Free Energy= -194.034762

C	-0.03204	0.83323	-0.57221
C	1.30621	0.8271	-0.35761
C	1.75514	2.2525	0.01266
C	0.56916	3.10434	-0.4754
C	-0.50584	2.29711	-0.64793
H	-0.65144	-0.03154	-0.68801
H	1.94617	-0.02657	-0.43883
H	1.85482	2.33968	1.07444
H	2.68483	2.53007	-0.43847
H	0.5915	4.15886	-0.65532
H	-1.51004	2.62121	-0.82523

1,3-Cyclopentadiene
The number of imaginary frequencies: 0
Total Energy (B3LYP/6-31G**)= -194.11069027
ZPE= -194.017995
Enthalpy= -194.012901
Gibb's Free Energy= -194.044595

C	-0.03204	0.83323	-0.57221
C	1.30621	0.8271	-0.35761
C	1.75514	2.2525	0.01266
C	0.56916	3.10434	-0.4754
C	-0.50584	2.29711	-0.64793
H	-0.65144	-0.03154	-0.68801
H	1.94617	-0.02657	-0.43883
H	1.85482	2.33968	1.07444
H	2.68483	2.53007	-0.43847
H	0.5915	4.15886	-0.65532
H	-1.51004	2.62121	-0.82523

1,3-Cyclopentadiene
The number of imaginary frequencies: 0
Total Energy (M06/6-311G+**)= -193.99039929
ZPE= -193.898575
Enthalpy= -193.893442
Gibb's Free Energy= -193.925181

C	-0.03204	0.83323	-0.57221
C	1.30621	0.8271	-0.35761
C	1.75514	2.2525	0.01266
C	0.56916	3.10434	-0.4754
C	-0.50584	2.29711	-0.64793
H	-0.65144	-0.03154	-0.68801
H	1.94617	-0.02657	-0.43883
H	1.85482	2.33968	1.07444
H	2.68483	2.53007	-0.43847
H	0.5915	4.15886	-0.65532
H	-1.51004	2.62121	-0.82523

1,3,5-Cycloheptatriene
The number of imaginary frequencies: 0
Total Energy (B3LYP/6-31G*)= -271.50958752
ZPE=-271.381192
Enthalpy= -271.374505
Gibb's Free Energy= -271.41033

C	-0.20071	-0.81856	0.32651
C	1.06397	-0.49637	-0.04424
C	1.68974	0.89155	-0.31285
C	-1.34606	0.10738	0.76151
C	1.19882	2.07325	0.5547
C	-1.27725	1.41867	1.06715
C	-0.03587	2.32272	1.05869
H	1.5021	1.14001	-1.33655
H	-0.43367	-1.86275	0.30957
H	1.721	-1.32797	-0.19136
H	2.74077	0.79537	-0.13682
H	-2.31134	-0.34609	0.84812
H	1.9384	2.81469	0.77422
H	-2.19448	1.88081	1.36717
H	-0.16354	3.28474	1.50936

1,3,5-Cycloheptatriene
The number of imaginary frequencies: 0
Total Energy (B3LYP/6-31G**)= -271.52195835
ZPE=-271.393853
Enthalpy= -271.387159
Gibb's Free Energy= -271.422993

C	-0.20071	-0.81856	0.32651
C	1.06397	-0.49637	-0.04424
C	1.68974	0.89155	-0.31285
C	-1.34606	0.10738	0.76151
C	1.19882	2.07325	0.5547
C	-1.27725	1.41867	1.06715
C	-0.03587	2.32272	1.05869
H	1.5021	1.14001	-1.33655
H	-0.43367	-1.86275	0.30957
H	1.721	-1.32797	-0.19136
H	2.74077	0.79537	-0.13682
H	-2.31134	-0.34609	0.84812
H	1.9384	2.81469	0.77422
H	-2.19448	1.88081	1.36717
H	-0.16354	3.28474	1.50936

1,3,5-Cycloheptatriene
The number of imaginary frequencies: 0
Total Energy (M06/6-311G+**)= -271.35461618
ZPE=-271.227611
Enthalpy= -271.22088
Gibb's Free Energy=-271.256734

C	-0.20071	-0.81856	0.32651
C	1.06397	-0.49637	-0.04424
C	1.68974	0.89155	-0.31285
C	-1.34606	0.10738	0.76151
C	1.19882	2.07325	0.5547
C	-1.27725	1.41867	1.06715
C	-0.03587	2.32272	1.05869
H	1.5021	1.14001	-1.33655
H	-0.43367	-1.86275	0.30957
H	1.721	-1.32797	-0.19136
H	2.74077	0.79537	-0.13682
H	-2.31134	-0.34609	0.84812
H	1.9384	2.81469	0.77422

H	-2.19448	1.88081	1.36717
H	-0.16354	3.28474	1.50936

2,4-Cyclopentadienone
The number of imaginary frequencies: 0
Total Energy (B3LYP/6-31G*)= -268.10366059
ZPE=-268.029249
Enthalpy= -268.023604
Gibb's Free Energy= -268.057109

C	-0.01597	0.56911	-0.63699
C	1.53525	0.57813	-0.59521
C	1.96549	1.79004	-0.15552
C	0.72782	2.6481	0.11742
C	-0.48331	1.77579	-0.22148
H	-0.62226	-0.25716	-0.94455
H	2.16679	-0.24094	-0.86942
H	2.9842	2.08978	-0.02407
H	-1.51104	2.06363	-0.14516
O	0.70962	3.83439	0.53688

2,4-Cyclopentadienone
The number of imaginary frequencies: 0
Total Energy (B3LYP/6-31G**)= -268.11018199
ZPE=-268.035857
Enthalpy= -268.030211
Gibb's Free Energy= -268.063716

C	-0.01597	0.56911	-0.63699
C	1.53525	0.57813	-0.59521
C	1.96549	1.79004	-0.15552
C	0.72782	2.6481	0.11742
C	-0.48331	1.77579	-0.22148
H	-0.62226	-0.25716	-0.94455
H	2.16679	-0.24094	-0.86942
H	2.9842	2.08978	-0.02407
H	-1.51104	2.06363	-0.14516
O	0.70962	3.83439	0.53688

2,4-Cyclopentadienone
The number of imaginary frequencies: 0
Total Energy (M06/6-311G+**)=-267.98852408
ZPE=-267.914874
Enthalpy= -267.909179
Gibb's Free Energy= -267.942763

C	-0.01597	0.56911	-0.63699
C	1.53525	0.57813	-0.59521
C	1.96549	1.79004	-0.15552
C	0.72782	2.6481	0.11742
C	-0.48331	1.77579	-0.22148
H	-0.62226	-0.25716	-0.94455
H	2.16679	-0.24094	-0.86942
H	2.9842	2.08978	-0.02407
H	-1.51104	2.06363	-0.14516
O	0.70962	3.83439	0.53688

2,4,6-Cycloheptatrienone
The number of imaginary frequencies: 0
Total Energy (B3LYP/6-31G*)= -345.53739483
ZPE= -345.426757
Enthalpy= -345.41951
Gibb's Free Energy= -345.457457

C	0.4044	-0.40001	-0.15775
C	1.77241	-0.40055	-0.15529
C	2.69105	0.61692	0.1737
C	-0.51461	0.61819	0.16794
C	2.45079	1.88455	0.58091
C	-0.2748	1.88563	0.576
C	1.08787	2.54419	0.79042
H	-0.04191	-1.32554	-0.45626
H	2.21906	-1.32643	-0.4522
H	3.72353	0.34896	0.08952
H	-1.54699	0.35106	0.08004
H	3.3092	2.49215	0.77797
H	-1.13344	2.49392	0.76997
O	1.08765	3.74217	1.17569

2,4,6-Cycloheptatrienone
The number of imaginary frequencies: 0
Total Energy (B3LYP/6-31G**)= -345.54656227
ZPE=-345.436187
Enthalpy=-345.428926
Gibb's Free Energy= -345.466909

C	0.4044	-0.40001	-0.15775
C	1.77241	-0.40055	-0.15529
C	2.69105	0.61692	0.1737
C	-0.51461	0.61819	0.16794
C	2.45079	1.88455	0.58091
C	-0.2748	1.88563	0.576
C	1.08787	2.54419	0.79042
H	-0.04191	-1.32554	-0.45626
H	2.21906	-1.32643	-0.4522
H	3.72353	0.34896	0.08952
H	-1.54699	0.35106	0.08004
H	3.3092	2.49215	0.77797
H	-1.13344	2.49392	0.76997
O	1.08765	3.74217	1.17569

2,4,6-Cycloheptatrienone
The number of imaginary frequencies: 0

Total Energy (M06/6-311G+**)= -345.37465529
ZPE=-345.265377
Enthalpy=-345.257928
Gibb's Free Energy= -345.297053

C	0.4044	-0.40001	-0.15775
C	1.77241	-0.40055	-0.15529
C	2.69105	0.61692	0.1737
C	-0.51461	0.61819	0.16794
C	2.45079	1.88455	0.58091
C	-0.2748	1.88563	0.576
C	1.08787	2.54419	0.79042
H	-0.04191	-1.32554	-0.45626
H	2.21906	-1.32643	-0.4522
H	3.72353	0.34896	0.08952
H	-1.54699	0.35106	0.08004
H	3.3092	2.49215	0.77797
H	-1.13344	2.49392	0.76997
O	1.08765	3.74217	1.17569

2,3-Epoxide N-Methylazepine
The number of imaginary frequencies: 0
Total Energy (B3LYP/6-31G*)= -402.0126142
ZPE= -401.86298
Enthalpy= -401.854323
Gibb's Free Energy= -401.894916

C	-1.97153	-0.86665	0.21864
C	-2.0792	0.477	0.09836
C	-0.81657	1.03296	-0.45425
C	-0.76447	-1.68529	-0.03687
C	0.35442	0.76433	0.37226
C	0.56633	-1.4101	-0.22187
H	-2.85437	-1.44535	0.48705
H	-2.99559	1.0283	0.28163
H	-0.97814	-2.75026	-0.09528
H	1.23067	-2.25728	-0.38004
N	1.2452	-0.18133	-0.21943
C	2.65217	-0.16427	0.16546
H	2.80272	-0.32861	1.24518
H	3.19049	-0.94245	-0.38388
H	3.08022	0.8047	-0.10428
O	0.07826	2.101	-0.03692
H	0.21628	0.62946	1.44876
H	-0.68167	0.83496	-1.51815

2,3-Epoxide N-Methylazepine
The number of imaginary frequencies: 0
Total Energy (B3LYP/6-31G**)= -402.02568814
ZPE= -401.876485
Enthalpy= -401.867821
Gibb's Free Energy= -401.908422

C	0.98337	1.56503	0.95991
C	1.51419	1.48097	-0.29992
C	0.77098	0.59913	-1.22425
C	-0.27211	0.73245	1.52549
C	0.33821	-0.65376	-0.82677
C	-1.08316	-0.29294	0.95543
H	1.45087	2.2507	1.63534
H	2.39553	2.01271	-0.59215
H	-0.54747	0.99384	2.52587
H	-1.84555	-0.69634	1.58863
N	-0.9916	-0.87598	-0.41373
C	-1.31788	-2.30927	-0.40307
H	-0.64737	-2.82283	0.25387
H	-2.32339	-2.44364	-0.06277
H	-1.22039	-2.70462	-1.39256
O	1.10315	-0.57651	-2.16386
H	0.91807	-1.18081	-0.09815
H	0.06457	1.25633	-1.68681

2,3-Epoxide N-Methylazepine
The number of imaginary frequencies: 0
Total Energy (M06/6-311G+**)= -401.83976324
ZPE= -401.691752
Enthalpy= -401.683102
Gibb's Free Energy= -401.72367

C	-1.97153	-0.86665	0.21864
C	-2.0792	0.477	0.09836
C	-0.81657	1.03296	-0.45425
C	-0.76447	-1.68529	-0.03687
C	0.35442	0.76433	0.37226
C	0.56633	-1.4101	-0.22187
H	-2.85437	-1.44535	0.48705
H	-2.99559	1.0283	0.28163
H	-0.97814	-2.75026	-0.09528
H	1.23067	-2.25728	-0.38004
N	1.2452	-0.18133	-0.21943
C	2.65217	-0.16427	0.16546
H	2.80272	-0.32861	1.24518
H	3.19049	-0.94245	-0.38388
H	3.08022	0.8047	-0.10428
O	0.07826	2.101	-0.03692
H	0.21628	0.62946	1.44876
H	-0.68167	0.83496	-1.51815

4,5-Epoxide N-Methylazepine
The number of imaginary frequencies: 0

Total Energy (B3LYP/6-31G*)= -402.04752008
ZPE= -401.897386
Enthalpy= -401.888458
Gibb’s Free Energy= -401.930104

C	-1.64868	-0.71215	0.39745
C	-1.655	0.71913	0.35793
C	-0.44281	1.62606	0.11899
C	-0.44434	-1.62081	0.12824
C	0.86032	1.29035	-0.06609
C	0.85605	-1.28843	-0.07968
H	-2.26809	-1.18847	1.12845
H	-0.65255	2.67515	0.10108
H	-0.6576	-2.66917	0.10912
H	1.55506	2.09233	0.07199
H	1.54753	-2.09766	0.02944
N	1.47105	0.0019	-0.46648
C	2.81324	-0.00515	0.133
H	3.34229	-0.87836	-0.18718
H	3.34692	0.86891	-0.177
H	2.72748	-0.01113	1.19955
H	-2.23316	1.23268	1.09746
O	-2.37974	-0.01144	-0.75584

4,5-Epoxide N-Methylazepine
The number of imaginary frequencies: 0
Total Energy (B3LYP/6-31G**)= -402.0607848
ZPE= -401.911089
Enthalpy=-401.902152
Gibb’s Free Energy= -401.943808

C	-1.62994	-0.73255	0.39014
C	-1.62993	0.73255	0.39016
C	-0.4817	1.5723	-0.0053
C	-0.48168	-1.57231	-0.00529
C	0.81741	1.23806	-0.12102
C	0.81742	-1.23805	-0.12103
H	-2.3009	-1.21971	1.10274
H	-0.71282	2.61654	-0.20338
H	-0.7128	-2.61655	-0.20337
H	1.52499	2.03453	-0.34095
H	1.52502	-2.03452	-0.34094
N	1.44508	0.00001	-0.0493
C	2.8978	0.	0.11175
H	3.32482	-0.88576	-0.36736
H	3.3248	0.88584	-0.36723
H	3.19608	-0.00007	1.16878
H	-2.30097	1.21969	1.1027
O	-2.35501	-0.00001	-0.63029

4,5-Epoxide N-Methylazepine
The number of imaginary frequencies: 0

Total Energy (M06/6-311G+**)= -401.87335865
ZPE= -401.724678
Enthalpy= -401.715624
Gibb’s Free Energy= -401.757962

C	-1.62994	-0.73255	0.39014
C	-1.62993	0.73255	0.39016
C	-0.4817	1.5723	-0.0053
C	-0.48168	-1.57231	-0.00529
C	0.81741	1.23806	-0.12102
C	0.81742	-1.23805	-0.12103
H	-2.3009	-1.21971	1.10274
H	-0.71282	2.61654	-0.20338
H	-0.7128	-2.61655	-0.20337
H	1.52499	2.03453	-0.34095
H	1.52502	-2.03452	-0.34094
N	1.44508	0.00001	-0.0493
C	2.8978	0.	0.11175
H	3.32482	-0.88576	-0.36736
H	3.3248	0.88584	-0.36723
H	3.19608	-0.00007	1.16878
H	-2.30097	1.21969	1.1027
O	-2.35501	-0.00001	-0.63029

N-methylpyrrolidine
The number of imaginary frequencies: 0
Total Energy (B3LYP/6-31G*)= -251.88956837
ZPE= -251.731361
Enthalpy= -251.724164
Gibb’s Free Energy=-251.761212

C	-1.15663	-0.45327	0.19297
C	1.15639	-0.45362	0.19329
C	0.77798	1.0303	-0.07053
C	-0.77756	1.03066	-0.07012
H	-2.05637	-0.7771	-0.33946
H	-1.33544	-0.61146	1.26475
H	1.33453	-0.61147	1.26524
H	2.05631	-0.77799	-0.33851
H	1.19788	1.70083	0.6869
H	1.1653	1.36132	-1.04085
H	-1.19669	1.70085	0.68805
H	-1.16528	1.36262	-1.03996
N	-0.00017	-1.27389	-0.20026
C	0.00003	-1.3903	-1.66564
H	0.87482	-1.91947	-1.98127
H	-0.87248	-1.92325	-1.98122
H	-0.0021	-0.41291	-2.10107

N-methylpyrrolidine
The number of imaginary frequencies: 0

Total Energy (B3LYP/6-31G**)= -251.904379
ZPE= -251.746764
Enthalpy= -251.739572
Gibb's Free Energy=-251.776517

C	-0.07138	1.15044	-0.53372
C	-0.07129	-1.15029	-0.53401
C	-1.2628	-0.77695	0.3945
C	-1.26312	0.77669	0.39438
H	0.45507	2.05955	-0.22703
H	-0.43402	1.30923	-1.55803
H	-0.43417	-1.30864	-1.55831
H	0.45527	-2.0595	-0.22783
H	-2.20509	-1.19792	0.02894
H	-1.11008	-1.16732	1.4062
H	-2.20542	1.19716	0.02825
H	-1.11112	1.16732	1.40608
N	0.84465	0.00011	-0.54566
C	1.76509	0.	0.59063
H	2.40898	-0.88422	0.53277
H	2.40905	0.88419	0.53288
H	1.27997	-0.00004	1.58496

N-methylpyrrolidine
The number of imaginary frequencies: 0
Total Energy (M06/6-311G+**)= -251.7568498
ZPE= -251.601035
Enthalpy= -251.593775
Gibb's Free Energy= -251.630876

C	-0.07138	1.15044	-0.53372
C	-0.07129	-1.15029	-0.53401
C	-1.2628	-0.77695	0.3945
C	-1.26312	0.77669	0.39438
H	0.45507	2.05955	-0.22703
H	-0.43402	1.30923	-1.55803
H	-0.43417	-1.30864	-1.55831
H	0.45527	-2.0595	-0.22783
H	-2.20509	-1.19792	0.02894
H	-1.11008	-1.16732	1.4062
H	-2.20542	1.19716	0.02825
H	-1.11112	1.16732	1.40608
N	0.84465	0.00011	-0.54566
C	1.76509	0.	0.59063
H	2.40898	-0.88422	0.53277
H	2.40905	0.88419	0.53288
H	1.27997	-0.00004	1.58496

N-methylpyrrolidine N-oxide
The number of imaginary frequencies: 0

Total Energy (B3LYP/6-31G*)= -327.03549275
ZPE= -326.872485
Enthalpy= -326.864715
Gibb's Free Energy= -326.90296

C	-1.15663	-0.45327	0.19297
C	1.15639	-0.45362	0.19329
C	0.77798	1.0303	-0.07053
C	-0.77756	1.03066	-0.07012
H	-2.05637	-0.7771	-0.33946
H	-1.33544	-0.61146	1.26475
H	1.33453	-0.61147	1.26524
H	2.05631	-0.77799	-0.33851
H	1.19788	1.70083	0.6869
H	1.1653	1.36132	-1.04085
H	-1.19669	1.70085	0.68805
H	-1.16528	1.36262	-1.03996
N	-0.00017	-1.27389	-0.20026
C	0.00003	-1.3903	-1.66564
H	0.87482	-1.91947	-1.98127
H	-0.87248	-1.92325	-1.98122
H	-0.0021	-0.41291	-2.10107
O	-0.00044	-2.50637	0.37472

N-methylpyrrolidine N-oxide
The number of imaginary frequencies: 0
Total Energy (B3LYP/6-31G**)= -327.05036743
ZPE= -326.888069
Enthalpy= -326.880274
Gibb's Free Energy= -326.918536

C	-0.07138	1.15044	-0.53372
C	-0.07129	-1.15029	-0.53401
C	-1.2628	-0.77695	0.3945
C	-1.26312	0.77669	0.39438
H	0.45507	2.05955	-0.22703
H	-0.43402	1.30923	-1.55803
H	-0.43417	-1.30864	-1.55831
H	0.45527	-2.0595	-0.22783
H	-2.20509	-1.19792	0.02894
H	-1.11008	-1.16732	1.4062
H	-2.20542	1.19716	0.02825
H	-1.11112	1.16732	1.40608
N	0.84465	0.00011	-0.54566
C	1.76509	0.	0.59063
H	2.40898	-0.88422	0.53277
H	2.40905	0.88419	0.53288
H	1.27997	-0.00004	1.58496

N-methylpyrrolidine N-oxide
The number of imaginary frequencies: 0

Total Energy (M06/6-311G+**)= -326.90051885
ZPE= -326.739215
Enthalpy= -326.731606
Gibb's Free Energy= -326.769334

C	-0.20642	1.18006	-0.39819
C	-0.20641	-1.17988	-0.39867
C	-1.60313	-0.78221	0.08929
C	-1.60323	0.78213	0.08935
H	0.22053	2.074	0.05958
H	-0.14015	1.27502	-1.4834
H	-0.14032	-1.27427	-1.48394
H	0.22064	-2.07405	0.05855
H	-2.37717	-1.19037	-0.56706
H	-1.80187	-1.16923	1.09398
H	-2.37711	1.1902	-0.56724
H	-1.80238	1.16905	1.094
N	0.71326	0.00002	-0.10279
C	1.10744	-0.00026	1.34796
H	1.72001	-0.88912	1.49744
H	1.72009	0.8885	1.49774
H	0.24363	-0.00033	2.02335
O	1.82397	0.00017	-0.86024

N-methyl-2-pyrroline
The number of imaginary frequencies: 0
Total Energy (B3LYP/6-31G*)= -250.66822024
ZPE= -250.534319
Enthalpy= -250.527508
Gibb's Free Energy= -250.563344

C	-0.02855	-0.57318	-0.15105
C	1.26736	-0.4801	-0.57951
C	1.79391	0.87643	-0.11602
C	0.48097	1.64558	-0.06848
N	-0.40721	0.67078	0.57784
H	-0.67354	-1.41071	-0.31672
H	1.80823	-1.21651	-1.13634
H	2.51541	1.32205	-0.76853
H	0.13582	1.83115	-1.06414
H	2.23671	0.78728	0.85396
H	0.54979	2.5825	0.44371
C	-1.82735	1.03316	0.46483
H	-2.08752	1.13839	-0.56772
H	-2.42756	0.26588	0.90747
H	-2.00069	1.95899	0.97246

N-methyl-2-pyrroline
The number of imaginary frequencies: 0
Total Energy (B3LYP/6-31G**)= -250.68085752
ZPE= -250.547406
Enthalpy= -250.540583
Gibb's Free Energy= -250.576436

C	0.10596	1.1811	-0.02571
C	1.41809	0.82958	0.13671
C	1.51846	-0.66607	-0.15518
C	0.10274	-1.08391	0.21738
N	-0.69193	-0.01873	-0.4077
H	-0.28835	2.16721	0.10471
H	2.22092	1.47863	0.41794
H	2.26785	-1.18171	0.40825
H	-0.01891	-1.03759	1.27943
H	1.71863	-0.8255	-1.19413
H	-0.1541	-2.0704	-0.10785
C	-2.08449	0.00233	0.06267
H	-2.09981	0.10969	1.12716
H	-2.60019	0.82538	-0.38628
H	-2.5671	-0.91275	-0.21051

N-methyl-2-pyrroline
The number of imaginary frequencies: 0
Total Energy (M06/6-311+*G*)= -250.53777695
ZPE= -250.405638
Enthalpy= -250.398822
Gibb's Free Energy= -250.434595

C	-0.02855	-0.57318	-0.15105
C	1.26736	-0.4801	-0.57951
C	1.79391	0.87643	-0.11602
C	0.48097	1.64558	-0.06848
N	-0.40721	0.67078	0.57784
H	-0.67354	-1.41071	-0.31672
H	1.80823	-1.21651	-1.13634
H	2.51541	1.32205	-0.76853
H	0.13582	1.83115	-1.06414
H	2.23671	0.78728	0.85396
H	0.54979	2.5825	0.44371
C	-1.82735	1.03316	0.46483
H	-2.08752	1.13839	-0.56772
H	-2.42756	0.26588	0.90747
H	-2.00069	1.95899	0.97246

N-methyl-2-pyrroline N-oxide
The number of imaginary frequencies: 0
Total Energy (B3LYP/6-31G*)= -325.80112215
ZPE= -325.662988
Enthalpy= -325.655506
Gibb's Free Energy= -325.693031

C	-0.03777	-0.57726	-0.15465
C	1.26273	-0.48143	-0.57967
C	1.79166	0.88052	-0.11066
C	0.47827	1.64772	-0.07115
N	-0.39638	0.66679	0.57367
H	-0.6877	-1.41074	-0.32137
H	1.8054	-1.21671	-1.13624
H	2.51631	1.32576	-0.75993
H	0.13792	1.82472	-1.07001
H	2.23026	0.79201	0.8613
H	0.5341	2.58902	0.43455
C	-1.81636	1.03147	0.46606
H	-2.07939	1.14064	-0.56534
H	-2.4163	0.26345	0.90778
H	-1.98697	1.95575	0.97742
O	-0.09195	0.52844	1.89192

N-methyl-2-pyrroline N-oxide

The number of imaginary frequencies: 0

Total Energy (B3LYP/6-31G**)= -325.81385944

ZPE= -325.676246

Enthalpy= -325.668741

Gibb’s Free Energy= -325.706303

C	-0.23379	1.18102	-0.17993
C	-1.52502	0.87048	-0.19439
C	-1.72932	-0.61116	0.01612
C	-0.32087	-1.17138	-0.21707
N	0.61792	0.00123	0.08597
H	0.2688	2.13771	-0.18968
H	-2.33435	1.58937	-0.23912
H	-2.45724	-1.05337	-0.67185
H	-0.19276	-1.46986	-1.26185
H	-2.07849	-0.80218	1.03742
H	-0.00953	-1.96861	0.45469
C	1.84379	-0.04037	-0.77097
H	1.5974	-0.0178	-1.83721
H	2.44721	0.82048	-0.48615
H	2.37766	-0.95301	-0.50559
O	0.98089	0.04214	1.39687

N-methyl-2-pyrroline N-oxide

The number of imaginary frequencies: 0

Total Energy (M06/6-311+*G*)= -325.66847024

ZPE= -325.531815

Enthalpy= -325.524448

Gibb’s Free Energy= -325.56161

C	-0.03777	-0.57726	-0.15465
C	1.26273	-0.48143	-0.57967
C	1.79166	0.88052	-0.11066
C	0.47827	1.64772	-0.07115
N	-0.39638	0.66679	0.57367
H	-0.6877	-1.41074	-0.32137
H	1.8054	-1.21671	-1.13624
H	2.51631	1.32576	-0.75993
H	0.13792	1.82472	-1.07001
H	2.23026	0.79201	0.8613
H	0.5341	2.58902	0.43455
C	-1.81636	1.03147	0.46606
H	-2.07939	1.14064	-0.56534
H	-2.4163	0.26345	0.90778
H	-1.98697	1.95575	0.97742
O	-0.09195	0.52844	1.89192

N-methyl-3-pyrroline

The number of imaginary frequencies: 0

Total Energy (B3LYP/6-31G*)= -250.6603928

ZPE= -250.527109

Enthalpy= -250.520299

Gibb’s Free Energy= -250.556164

C	1.07009	-1.23529	-0.0877
C	2.59979	-1.2302	0.08945
C	3.00239	0.06358	0.08894
C	1.74564	0.93562	-0.08855
N	0.70686	0.06853	0.48636
H	0.58497	-2.04788	0.41156
H	3.22822	-2.08949	0.19723
H	4.00746	0.41463	0.19625
H	1.56874	1.11503	-1.12846
C	-0.66822	0.49631	0.19132
H	-0.83888	1.46456	0.61354
H	-0.80863	0.53959	-0.86855
H	-1.35805	-0.20383	0.6142
H	0.82239	-1.28338	-1.12752
H	1.80737	1.88035	0.41002

N-methyl-3-pyrroline

The number of imaginary frequencies: 0

Total Energy (B3LYP/6-31G**)= -250.67291059

ZPE= -250.540105

Enthalpy= -250.533291

Gibb’s Free Energy= -250.569157

C	0.01026	-0.11685	1.19135
C	0.01026	-1.52903	0.66633
C	0.01026	-1.52903	-0.66633
C	0.01026	-0.11685	-1.19135
N	0.32891	0.68022	0.
H	0.74953	0.05319	1.98809
H	-0.01543	-2.40073	1.31123
H	-0.01543	-2.40073	-1.31123
H	-0.98169	0.1315	-1.62443

C -0.18554 2.03252 0.
H 0.1764 2.56965 -0.88334
H -1.29288 2.08671 0.
H 0.1764 2.56965 0.88334
H -0.98169 0.1315 1.62443
H 0.74953 0.05319 -1.98809

N-methyl-3-pyrroline
The number of imaginary frequencies: 0
Total Energy (M06/6-311+*G*)= -250.53030478
ZPE= -250.398839
Enthalpy= -250.392016
Gibb's Free Energy= -250.427847

C -0.00138 -0.1147 1.17913
C -0.00138 -1.51818 0.66335
C -0.00138 -1.51818 -0.66335
C -0.00138 -0.1147 -1.17913
N 0.33927 0.66756 0.
H 0.71625 0.05989 1.99335
H -0.03111 -2.39025 1.3058
H -0.03111 -2.39025 -1.3058
H -1.00567 0.14611 -1.5787
C -0.14704 2.01858 0.
H 0.21801 2.55165 -0.88417
H -1.25452 2.07526 0.
H 0.21801 2.55165 0.88417
H -1.00567 0.14611 1.5787
H 0.71625 0.05989 -1.99335

N-methyl-3-pyrroline N-oxide
The number of imaginary frequencies: 0
Total Energy (B3LYP/6-31G*)= -325.80062068
ZPE= -325.662821
Enthalpy= -325.655372
Gibb's Free Energy= -325.692895

C 1.05926 -1.23237 -0.09866
C 2.58767 -1.2308 0.1087
C 2.99275 0.071 0.10821
C 1.73505 0.93937 -0.09948
N 0.711 0.06723 0.47757
H 0.55417 -2.04303 0.38364
H 3.21271 -2.0904 0.23245
H 3.9952 0.42426 0.2315
H 1.56998 1.10051 -1.14432
C -0.66662 0.4958 0.1958
H -0.83355 1.46287 0.62216
H -0.81634 0.54199 -0.86267
H -1.35271 -0.20552 0.62279
H 0.83169 -1.27213 -1.14343
H 1.7792 1.89382 0.38215
O 0.87454 0.01685 1.82676

N-methyl-3-pyrroline N-oxide
The number of imaginary frequencies: 0
Total Energy (B3LYP/6-31G**)= -325.81340875
ZPE= -325.676093
Enthalpy= -325.668646
Gibb's Free Energy= -325.706136

C 0.22103 0.28223 1.21642
C 0.22103 1.67477 0.66657
C 0.22103 1.67477 -0.66657
C 0.22103 0.28223 -1.21642
N -0.15212 -0.58721 0.
H -0.55767 0.06533 1.94953
H 0.20969 2.55257 1.30413
H 0.20969 2.55257 -1.30413
H 1.20189 -0.01004 -1.61561
C 0.60337 -1.88315 0.
H 0.28233 -2.42675 -0.88876
H 1.68864 -1.73164 0.
H 0.28233 -2.42675 0.88876
H 1.20189 -0.01004 1.61561
H -0.55767 0.06533 -1.94953
O -1.47766 -0.83814 0.

N-methyl-3-pyrroline N-oxide
The number of imaginary frequencies: 0
Total Energy (M06/6-311+*G*)= -325.6680588
ZPE= -325.53171
Enthalpy= -325.524342
Gibb's Free Energy= -325.561595

C 1.05926 -1.23237 -0.09866
C 2.58767 -1.2308 0.1087
C 2.99275 0.071 0.10821
C 1.73505 0.93937 -0.09948
N 0.711 0.06723 0.47757
H 0.55417 -2.04303 0.38364
H 3.21271 -2.0904 0.23245
H 3.9952 0.42426 0.2315
H 1.56998 1.10051 -1.14432
C -0.66662 0.4958 0.1958
H -0.83355 1.46287 0.62216
H -0.81634 0.54199 -0.86267
H -1.35271 -0.20552 0.62279
H 0.83169 -1.27213 -1.14343

H	1.7792	1.89382	0.38215
O	0.87454	0.01685	1.82676

N-methylazepane
The number of imaginary frequencies: 0
Total Energy (B3LYP/6-31G*)= -330.51413505
ZPE= -330.297713
Enthalpy= -330.288427
Gibb's Free Energy= -330.330325

C	0.73212	1.51003	-0.4035
C	-0.71609	1.25523	0.03895
C	1.7927	0.77847	0.43621
C	-0.52079	-1.191	0.31221
C	2.04124	-0.69458	0.05474
C	0.80307	-1.48614	-0.4115
N	-1.26022	-0.06586	-0.27351
C	-2.67256	-0.11883	0.08393
H	-3.21445	0.67915	-0.4361
H	-3.10036	-1.07735	-0.22923
H	-2.85932	0.00024	1.1703
H	-0.35545	-1.0579	1.40146
H	-1.16564	-2.07111	0.20855
H	1.02594	-2.55654	-0.31156
H	0.62107	-1.31016	-1.47825
H	2.74655	1.31783	0.37304
H	1.48852	0.83963	1.4906
H	0.89962	2.59119	-0.30925
H	0.83983	1.27152	-1.46966
H	-0.78672	1.46965	1.12797
H	-1.36351	1.98763	-0.46024
H	2.79112	-0.74141	-0.74624
H	2.49617	-1.20044	0.91699

N-methylazepane
The number of imaginary frequencies: 0
Total Energy (B3LYP/6-31G**)= -330.53806647
ZPE= -330.322355
Enthalpy= -330.313145
Gibb's Free Energy= -330.354675

C	0.7311	1.51068	-0.40214
C	-0.71639	1.25629	0.04082
C	1.79174	0.77753	0.43555
C	-0.51944	-1.18994	0.31525
C	2.04163	-0.69371	0.05083
C	0.80285	-1.48699	-0.41006
N	-1.25882	-0.06547	-0.27216
C	-2.67258	-0.12059	0.08033
H	-3.21461	0.67542	-0.44073
H	-3.09839	-1.07873	-0.23368
H	-2.86468	-0.00198	1.16495
H	-0.35201	-1.05591	1.40334
H	-1.16385	-2.06989	0.21472
H	1.02568	-2.55627	-0.30792
H	0.61762	-1.31452	-1.47605
H	2.74434	1.31751	0.37445
H	1.48778	0.83602	1.48928
H	0.8994	2.59073	-0.30712
H	0.83784	1.27383	-1.468
H	-0.78576	1.47129	1.12915
H	-1.36395	1.98832	-0.45733
H	2.78754	-0.73783	-0.75285
H	2.50134	-1.19937	0.90949

N-methylazepane
The number of imaginary frequencies: 0
Total Energy (M06/6-311G+**)= -330.32162526
ZPE= -330.107821
Enthalpy= -330.09853
Gibb's Free Energy= -330.140342

C	1.51265	0.65777	0.68028
C	1.78687	-0.75362	0.1749
C	0.55687	-1.62184	-0.03627
C	0.78962	1.53617	-0.33554
C	-0.53615	-0.9995	-0.90604
C	-0.73095	1.41941	-0.33053
H	0.94042	0.62182	1.62108
H	2.33902	-0.67556	-0.77611
H	0.12066	-1.891	0.93777
H	-0.10723	-0.64267	-1.8541
H	1.18303	1.31841	-1.33996
H	-1.12031	1.98155	-1.19752
H	2.47396	1.12315	0.93659
H	2.46827	-1.2637	0.87008
H	0.86559	-2.57467	-0.49074
H	1.02842	2.59515	-0.16187
H	-1.25018	-1.7895	-1.18581
H	-1.11088	1.94537	0.55708
N	-1.29038	0.07892	-0.30783
C	-2.04279	-0.26955	0.86647
H	-2.8496	0.45522	1.02999
H	-2.51372	-1.25069	0.72696
H	-1.45141	-0.31833	1.80185

N-methylazepane N-oxide
The number of imaginary frequencies: 0
Total Energy (B3LYP/6-31G*)= -405.65530091
ZPE= -405.433782
Enthalpy= -405.424009

Gibb’s Free Energy= -405.46685

C	-0.68389	-0.0867	-0.0655
C	0.86341	-0.02909	-0.0243
C	1.52087	1.3621	0.05709
C	-1.21057	0.349	1.28887
C	1.10761	2.30191	1.22874
C	-1.33626	1.86899	1.50908
H	-1.10813	0.4975	-0.85521
H	1.16889	-0.58993	0.83421
H	1.29525	1.88657	-0.84785
H	1.22426	1.8509	2.19201
H	-0.53585	-0.07286	2.00419
H	-1.42434	1.98096	2.56955
H	-0.94122	-1.11544	-0.20832
H	1.22986	-0.48397	-0.92079
H	2.57332	1.18802	0.14045
H	-2.1988	-0.03456	1.43448
H	1.74807	3.15582	1.15428
H	-2.21801	2.2191	1.01425
N	-0.26066	2.75574	1.02141
C	-0.38413	2.94763	-0.43077
H	-1.35701	3.33045	-0.6585
H	0.35913	3.64182	-0.76328
H	-0.2444	2.01031	-0.92755
O	-0.45463	3.92978	1.6799

N-methylazepane N-oxide

The number of imaginary frequencies: 0

Total Energy (B3LYP/6-31G**)= -405.68570133

ZPE= -405.465245

Enthalpy= -405.455415

Gibb’s Free Energy= -405.498192

C	-0.87731	1.5007	0.36873
C	0.51088	1.33419	-0.23712
C	-2.00082	0.77897	-0.39556
C	0.32701	-1.17285	-0.5794
C	-2.18916	-0.71157	-0.04683
C	-0.90207	-1.5005	0.25743
N	1.14604	-0.0214	0.04394
C	2.50298	-0.03427	-0.59869
H	3.06617	0.79804	-0.17874
H	2.97609	-0.96957	-0.30406
H	2.43584	0.04876	-1.68895
H	0.10455	-0.91863	-1.62206
H	1.01517	-2.01981	-0.56491
H	-1.11006	-2.57108	0.1513
H	-0.57176	-1.34045	1.28934
H	-2.95098	1.29611	-0.21842
H	-1.80879	0.88583	-1.47256
H	-1.08295	2.57776	0.37236
H	-0.81883	1.18252	1.41452
H	0.49467	1.50062	-1.32195
H	1.20591	2.03715	0.22537
H	-2.84025	-0.79465	0.83193
H	-2.73665	-1.18964	-0.86925
O	1.2963	-0.19264	1.37965

N-methylazepane N-oxide

The number of imaginary frequencies: 0

Total Energy (M06/6-311G+**)= -405.47684909

ZPE= -405.258482

Enthalpy= -405.24872

Gibb’s Free Energy= -405.291518

C	-0.68389	-0.0867	-0.0655
C	0.86341	-0.02909	-0.0243
C	1.52087	1.3621	0.05709
C	-1.21057	0.349	1.28887
C	1.10761	2.30191	1.22874
C	-1.33626	1.86899	1.50908
H	-1.10813	0.4975	-0.85521
H	1.16889	-0.58993	0.83421
H	1.29525	1.88657	-0.84785
H	1.22426	1.8509	2.19201
H	-0.53585	-0.07286	2.00419
H	-1.42434	1.98096	2.56955
H	-0.94122	-1.11544	-0.20832
H	1.22986	-0.48397	-0.92079
H	2.57332	1.18802	0.14045
H	-2.1988	-0.03456	1.43448
H	1.74807	3.15582	1.15428
H	-2.21801	2.2191	1.01425
N	-0.26066	2.75574	1.02141
C	-0.38413	2.94763	-0.43077
H	-1.35701	3.33045	-0.6585
H	0.35913	3.64182	-0.76328
H	-0.2444	2.01031	-0.92755
O	-0.45463	3.92978	1.6799

1-methyl-2,3,4,5-tetrahydro-1H-azepine

The number of imaginary frequencies: 0

Total Energy (B3LYP/6-31G*)= -329.29734188

ZPE=-329.104678

Enthalpy= -329.095641

Gibb’s Free Energy= -329.137249

C	-1.93668	0.79405	-0.16536
C	-1.91369	-0.66619	0.29955
C	-0.79253	-1.50046	-0.32623
C	-0.69176	1.60985	0.07132

C	0.60281	-1.21219	0.23494
C	0.60875	1.26272	0.01777
H	-2.16893	0.8014	-1.24481
H	-1.81819	-0.70387	1.39418
H	-0.7804	-1.37039	-1.41717
H	0.55268	-1.24755	1.34067
H	-2.78411	1.30138	0.31486
H	-2.88152	-1.12282	0.05332
H	-0.99385	-2.56353	-0.13769
H	-0.86179	2.67477	0.21549
H	1.27277	-2.02244	-0.07532
H	1.32978	2.07134	0.1237
N	1.23158	0.03078	-0.20714
C	2.67576	0.02755	-0.00353
H	3.11431	0.93087	-0.43659
H	2.96525	-0.02091	1.0617
H	3.11702	-0.83567	-0.51311

1-methyl-2,3,4,5-tetrahydro-1H-azepine
The number of imaginary frequencies: 0
Total Energy (B3LYP/6-31G**)= -329.31548004
ZPE=-329.123508
Enthalpy= -329.114461
Gibb’s Free Energy= -329.156073

C	-1.93249	0.79521	-0.17278
C	-1.91545	-0.66385	0.29603
C	-0.79336	-1.50269	-0.32111
C	-0.69029	1.61075	0.07493
C	0.60107	-1.21171	0.23984
C	0.60955	1.26312	0.02435
H	-2.15202	0.79989	-1.25399
H	-1.826	-0.69823	1.39045
H	-0.77885	-1.37979	-1.41203
H	0.54996	-1.2447	1.34503
H	-2.78407	1.30421	0.29592
H	-2.88213	-1.11899	0.04648
H	-0.99535	-2.56362	-0.12654
H	-0.86042	2.67389	0.22642
H	1.27056	-2.02283	-0.06704
H	1.33035	2.07028	0.13838
N	1.22934	0.03054	-0.20594
C	2.67513	0.02562	-0.01156
H	3.11207	0.92845	-0.44494
H	2.97241	-0.02535	1.05051
H	3.11319	-0.83562	-0.52531

1-methyl-2,3,4,5-tetrahydro-1H-azepine
The number of imaginary frequencies: 0
Total Energy (M06/6-311G+**)= -329.12249802
ZPE=-328.932494
Enthalpy= -328.923377
Gibb’s Free Energy= -328.965286

C	-1.93321	0.77636	-0.14385
C	-1.88554	-0.6686	0.31918
C	-0.78336	-1.47754	-0.34095
C	-0.69705	1.5925	0.05953
C	0.60182	-1.20425	0.21188
C	0.5986	1.25289	-0.00482
H	-2.18579	0.77908	-1.21868
H	-1.7509	-0.70474	1.41034
H	-0.78202	-1.3067	-1.42726
H	0.55727	-1.23898	1.31985
H	-2.7735	1.28283	0.34757
H	-2.85654	-1.13455	0.10971
H	-0.98156	-2.54636	-0.19233
H	-0.86444	2.65919	0.18934
H	1.27299	-2.01417	-0.09815
H	1.31253	2.07096	0.08133
N	1.22736	0.03279	-0.21823
C	2.65472	0.03368	0.02568
H	3.1068	0.94119	-0.38139
H	2.90006	-0.0243	1.10077
H	3.11779	-0.8232	-0.47342

1-methyl-2,3,4,5-tetrahydro-1H-azepine N-oxide
The number of imaginary frequencies: 0
Total Energy (B3LYP/6-31G*)= -404.42502271
ZPE=-404.228193
Enthalpy= -404.218652
Gibb’s Free Energy= -404.26106

C	0.49565	0.4599	-0.25257
C	1.9606	0.36039	0.11322
C	2.62584	1.76027	0.11912
C	-0.31101	1.01637	0.91325
C	2.07129	2.81315	1.12848
C	-0.36455	2.31347	1.21761
H	0.40807	1.08914	-1.11355
H	2.05677	-0.11513	1.06691
H	2.5273	2.17619	-0.8618
H	2.12361	2.40779	2.11735
H	0.10558	-0.51126	-0.47523
H	2.46182	-0.22127	-0.63201
H	3.65685	1.60579	0.36006
H	-0.87414	0.33125	1.5119
H	2.7101	3.66688	1.03929
H	-1.22433	2.65914	1.75256
N	0.67244	3.30432	0.89169
C	0.52912	3.73516	-0.50642
H	-0.45373	4.12962	-0.65912

H	1.25453	4.49139	-0.72275
H	0.68223	2.89808	-1.15508
O	0.47962	4.35813	1.72949

1-methyl-2,3,4,5-tetrahydro-1H-azepine N-oxide

The number of imaginary frequencies: 0
Total Energy (B3LYP/6-31G**)= -404.44322686
ZPE=-404.247194
Enthalpy= -404.237619
Gibb's Free Energy= -404.280085

C	1.96356	0.81312	0.33863
C	2.10879	-0.60245	-0.23877
C	0.95175	-1.54601	0.10421
C	0.81163	1.62637	-0.20456
C	-0.39354	-1.20835	-0.53642
C	-0.47022	1.30415	-0.36417
H	1.87606	0.74597	1.43407
H	2.21458	-0.53759	-1.32906
H	0.8498	-1.63399	1.19366
H	-0.30582	-1.05072	-1.61285
H	2.89003	1.36631	0.15354
H	3.04175	-1.03582	0.14115
H	1.20446	-2.55412	-0.24839
H	1.0502	2.64764	-0.4964
H	-1.11915	-2.00795	-0.38101
H	-1.21661	1.9886	-0.74614
N	-1.16189	0.01815	-0.06544
C	-1.45306	-0.0504	1.41038
H	-2.09483	0.80177	1.62617
H	-2.01237	-0.97213	1.57198
H	-0.5458	-0.01981	2.02088
O	-2.35157	0.01453	-0.74067

1-methyl-2,3,4,5-tetrahydro-1H-azepine N-oxide

The number of imaginary frequencies: 0
Total Energy (M06/6-311G+**)= -404.2475018
ZPE=-404.053202
Enthalpy= -404.043727
Gibb's Free Energy=-404.085774

C	1.77906	0.80797	0.47768
C	2.12165	-0.49627	-0.20889
C	0.96929	-1.52143	-0.05614
C	0.75836	1.59069	-0.33779
C	-0.4141	-1.14154	-0.66999
C	-0.54738	1.32049	-0.33598
H	1.399	0.58295	1.45227
H	2.33546	-0.30886	-1.24042
H	0.81786	-1.69384	0.98897
H	-0.28559	-0.92713	-1.71039
H	2.65349	1.41851	0.56438
H	2.98187	-0.91903	0.26673
H	1.2937	-2.41794	-0.54186
H	1.10631	2.40827	-0.93395
H	-1.03706	-2.00156	-0.5389
H	-1.21606	2.1179	-0.5848
N	-1.14134	0.00879	-0.03599
C	-1.20649	-0.18842	1.41927
H	-1.76721	0.60792	1.86236
H	-1.68405	-1.12208	1.63164
H	-0.21563	-0.19464	1.82306
O	-2.39439	0.01988	-0.56451

1-methyl-2,3,4,7-tetrahydro-1H-azepine

The number of imaginary frequencies: 0
Total Energy (B3LYP/6-31G*)= -329.28700224
ZPE=-329.094545
Enthalpy= -329.085733
Gibb's Free Energy= -329.126397

C	0.46295	0.17234	0.24131
C	1.97537	0.39078	0.04423
C	2.50256	1.83166	-0.01181
C	-0.37683	0.88382	1.02888
C	2.13658	2.73783	1.18466
C	-0.37265	2.36391	1.46212
H	2.45636	-0.09513	0.8673
H	2.13696	2.3141	-0.89413
H	2.30148	2.24591	2.12046
H	-0.43758	2.35657	2.53013
H	0.04036	-0.67463	-0.25765
H	2.25285	-0.06973	-0.88088
H	3.56837	1.74437	-0.04804
H	-1.18608	0.31194	1.43255
H	2.76419	3.60186	1.11792
H	-1.23298	2.83435	1.03385
N	0.75876	3.21509	1.05177
C	0.62038	3.49949	-0.38381
H	-0.33935	3.93424	-0.57039
H	1.38631	4.1821	-0.68762
H	0.71346	2.58915	-0.93835

1-methyl-2,3,4,7-tetrahydro-1H-azepine

The number of imaginary frequencies: 0
Total Energy (B3LYP/6-31G**)= -329.30507753
ZPE=-329.113362

Enthalpy= -329.104525
Gibb's Free Energy= -329.145233

C	-1.51433	0.99972	-0.29773
C	-1.85588	-0.45955	0.03708
C	-0.7246	-1.48048	-0.17996
C	-0.38439	1.68338	-0.01036
C	0.52496	-1.30198	0.70533
C	0.87964	1.19436	0.72044
H	-2.16918	-0.50204	1.05931
H	-0.41509	-1.44656	-1.20366
H	0.23039	-1.23137	1.73156
H	0.68845	1.21373	1.77305
H	-2.27829	1.55365	-0.80214
H	-2.65614	-0.75274	-0.60986
H	-1.13997	-2.43881	0.05236
H	-0.37641	2.71001	-0.31182
H	1.1295	-2.17173	0.55372
H	1.66412	1.87817	0.47169
N	1.36727	-0.14493	0.35048
C	1.56196	-0.17639	-1.10623
H	2.22667	0.61105	-1.39432
H	1.98159	-1.11897	-1.38971
H	0.61935	-0.04415	-1.59499

1-methyl-2,3,4,7-tetrahydro-1H-azepine
The number of imaginary frequencies: 0
Total Energy (M06/6-311G+**)= -329.11354081
ZPE=-328.923838
Enthalpy= -328.915004
Gibb's Free Energy= -328.955568

C	0.46295	0.17234	0.24131
C	1.97537	0.39078	0.04423
C	2.50256	1.83166	-0.01181
C	-0.37683	0.88382	1.02888
C	2.13658	2.73783	1.18466
C	-0.37265	2.36391	1.46212
H	2.45636	-0.09513	0.8673
H	2.13696	2.3141	-0.89413
H	2.30148	2.24591	2.12046
H	-0.43758	2.35657	2.53013
H	0.04036	-0.67463	-0.25765
H	2.25285	-0.06973	-0.88088
H	3.56837	1.74437	-0.04804
H	-1.18608	0.31194	1.43255
H	2.76419	3.60186	1.11792
H	-1.23298	2.83435	1.03385
N	0.75876	3.21509	1.05177
C	0.62038	3.49949	-0.38381
H	-0.33935	3.93424	-0.57039
H	1.38631	4.1821	-0.68762
H	0.71346	2.58915	-0.93835

1-methyl-2,3,4,7-tetrahydro-1H-azepine N-oxide
The number of imaginary frequencies: 0
Total Energy (B3LYP/6-31G*)= -404.43129113
ZPE=-404.234067
Enthalpy= -404.224616
Gibb's Free Energy= -404.266611

C	0.45874	0.16613	0.2198
C	1.97398	0.36932	0.06143
C	2.50955	1.80472	0.00757
C	-0.37234	0.87672	1.00869
C	2.14031	2.74606	1.1813
C	-0.34893	2.34664	1.4712
H	2.4313	-0.11719	0.89753
H	2.15919	2.27549	-0.88715
H	2.30708	2.2883	2.13395
H	-0.37312	2.30691	2.54019
H	0.03122	-0.66405	-0.30263
H	2.27038	-0.09692	-0.8549
H	3.57512	1.70908	-0.01004
H	-1.19202	0.30979	1.39806
H	2.77068	3.60456	1.07872
H	-1.22489	2.82669	1.0876
N	0.76135	3.22591	1.04414
C	0.60023	3.53211	-0.38456
H	-0.36828	3.95516	-0.55165
H	1.35178	4.23135	-0.68644
H	0.6999	2.63269	-0.95554
O	0.62535	4.34769	1.80092

1-methyl-2,3,4,7-tetrahydro-1H-azepine N-oxide
The number of imaginary frequencies: 0
Total Energy (B3LYP/6-31G**)= -404.44946699
ZPE=-404.253037
Enthalpy= -404.243554
Gibb's Free Energy= -404.285602

C	1.92307	0.87597	0.11409
C	2.09044	-0.62982	-0.10959
C	0.90465	-1.50203	0.33143
C	0.83399	1.6357	-0.12024
C	-0.4226	-1.28929	-0.42733
C	-0.54165	1.21328	-0.66868
H	2.27679	-0.7957	-1.1501
H	0.72165	-1.33798	1.37282
H	-0.2463	-1.3535	-1.48075

H	-0.45947	1.11461	-1.73094
H	2.78523	1.39413	0.47889
H	2.93107	-0.94405	0.47308
H	1.2025	-2.51523	0.15937
H	0.94975	2.68046	0.07972
H	-1.07357	-2.0774	-0.11109
H	-1.2242	1.99791	-0.41691
N	-1.1286	-0.02679	-0.1228
C	-1.22486	0.09889	1.33866
H	-1.79047	0.97314	1.58497
H	-1.71157	-0.76551	1.73969
H	-0.24261	0.18052	1.75509
O	-2.36594	-0.12909	-0.67789

1-methyl-2,3,4,7-tetrahydro-1H-azepine N-oxide

The number of imaginary frequencies: 0
Total Energy (M06/6-311G+**)= -404.25465228
ZPE=-404.059795
Enthalpy= -404.050431
Gibb’s Free Energy=-404.092144

C	0.45874	0.16613	0.2198
C	1.97398	0.36932	0.06143
C	2.50955	1.80472	0.00757
C	-0.37234	0.87672	1.00869
C	2.14031	2.74606	1.1813
C	-0.34893	2.34664	1.4712
H	2.4313	-0.11719	0.89753
H	2.15919	2.27549	-0.88715
H	2.30708	2.2883	2.13395
H	-0.37312	2.30691	2.54019
H	0.03122	-0.66405	-0.30263
H	2.27038	-0.09692	-0.8549
H	3.57512	1.70908	-0.01004
H	-1.19202	0.30979	1.39806
H	2.77068	3.60456	1.07872
H	-1.22489	2.82669	1.0876
N	0.76135	3.22591	1.04414
C	0.60023	3.53211	-0.38456
H	-0.36828	3.95516	-0.55165
H	1.35178	4.23135	-0.68644
H	0.6999	2.63269	-0.95554
O	0.62535	4.34769	1.80092

1-methyl-2,3,6,7-tetrahydro-1H-azepine

The number of imaginary frequencies: 0
Total Energy (B3LYP/6-31G*)= -329.28622475
ZPE=-329.093718
Enthalpy= -329.084945
Gibb’s Free Energy= -329.125486

C	0.65622	0.04817	0.22592
C	1.80932	0.47645	-0.33881
C	2.60467	1.76621	-0.02668
C	-0.01152	0.68273	1.44487
C	2.19411	2.5403	1.25704
C	-0.26028	2.20759	1.41592
H	2.46107	2.44806	-0.83869
H	2.22732	1.94966	2.14863
H	0.62496	0.45232	2.27355
H	-0.52473	2.45334	2.42318
H	0.2084	-0.83519	-0.17907
H	2.23622	-0.17011	-1.07679
H	3.63467	1.48634	0.04875
H	-0.97652	0.23944	1.57593
H	2.87517	3.35964	1.3557
H	-1.06514	2.43583	0.74883
N	0.8605	3.06522	1.00312
C	0.85443	3.27624	-0.45164
H	-0.07965	3.7075	-0.74559
H	1.65233	3.93692	-0.71954
H	0.9862	2.33791	-0.9487

1-methyl-2,3,6,7-tetrahydro-1H-azepine

The number of imaginary frequencies: 0
Total Energy (B3LYP/6-31G**)= -329.30420567
ZPE= -329.112519
Enthalpy= -329.103711
Gibb’s Free Energy= -329.144319

C	1.99797	-0.4302	-0.07796
C	1.76928	0.8603	-0.34826
C	0.45115	1.58846	-0.22273
C	0.97454	-1.48002	0.30279
C	-0.51524	0.94658	0.79829
C	-0.42038	-1.271	-0.29984
H	-0.03894	1.68785	-1.20393
H	0.0587	0.71542	1.70449
H	0.89381	-1.58222	1.39549
H	-0.97602	-2.21782	-0.25329
H	3.0231	-0.79045	-0.16407
H	2.61791	1.47108	-0.65701
H	0.65873	2.61831	0.10046
H	1.34125	-2.45151	-0.05297
H	-1.27386	1.68024	1.09353
H	-0.29162	-1.03931	-1.37605
N	-1.23345	-0.25848	0.37624
C	-2.43499	0.02608	-0.39246

H	-2.9834	-0.90701	-0.56853
H	-3.09029	0.69863	0.17161
H	-2.23924	0.48507	-1.38242

1-methyl-2,3,6,7-tetrahydro-1H-azepine
The number of imaginary frequencies: 0
Total Energy (M06/6-311G+**)= -329.11349961
ZPE=-328.923679
Enthalpy= -328.914937
Gibb’s Free Energy= -328.955271

C	1.98583	-0.41519	-0.07008
C	1.7457	0.86549	-0.34366
C	0.42498	1.56747	-0.23043
C	0.97413	-1.46395	0.2974
C	-0.51329	0.93413	0.79927
C	-0.40349	-1.25533	-0.30763
H	-0.07178	1.63208	-1.21126
H	0.06905	0.7131	1.70383
H	0.88737	-1.5684	1.38944
H	-0.95658	-2.20448	-0.29285
H	3.01627	-0.76022	-0.14325
H	2.59056	1.48325	-0.64527
H	0.61224	2.60884	0.06172
H	1.34832	-2.43297	-0.05422
H	-1.27575	1.66095	1.10211
H	-0.27248	-0.99307	-1.37912
N	-1.21805	-0.26762	0.3788
C	-2.41151	0.01528	-0.38202
H	-2.94669	-0.91825	-0.58815
H	-3.08156	0.66692	0.18734
H	-2.20669	0.49821	-1.35893

1-methyl-2,3,6,7-tetrahydro-1H-azepine N-oxide
The number of imaginary frequencies: 0
Total Energy (B3LYP/6-31G*)= -404.42326899
ZPE=-404.225979
Enthalpy= -404.216486
Gibb’s Free Energy= -404.259077

C	0.63577	0.05068	0.19107
C	1.81061	0.46269	-0.32535
C	2.616	1.74208	0.00561
C	-0.01818	0.66913	1.41353
C	2.20367	2.57041	1.26497
C	-0.23454	2.19901	1.44017
H	2.50056	2.40935	-0.82284
H	2.24317	2.03388	2.18989
H	0.61702	0.39986	2.23141
H	-0.43813	2.40267	2.47069
H	0.16758	-0.80395	-0.25087
H	2.25465	-0.192	-1.04585
H	3.63859	1.44571	0.11224
H	-0.99328	0.2458	1.53552
H	2.88742	3.39231	1.308
H	-1.06983	2.46574	0.82695
N	0.86699	3.07773	0.99765
C	0.83582	3.27861	-0.45823
H	-0.1133	3.68284	-0.74226
H	1.61213	3.95846	-0.74117
H	0.98595	2.34076	-0.95097
O	0.65027	4.25335	1.64615

1-methyl-2,3,6,7-tetrahydro-1H-azepine N-oxide
The number of imaginary frequencies: 0
Total Energy (B3LYP/6-31G**)= -404.44160371
ZPE=-404.245096
Enthalpy= -404.23558
Gibb’s Free Energy= -404.278124

C	2.00147	0.51596	0.29064
C	1.72629	-0.78975	0.30569
C	0.67339	-1.51602	-0.58728
C	1.07751	1.51412	-0.35375
C	-0.55367	-0.67522	-1.07595
C	-0.41156	1.41351	0.09795
H	0.29215	-2.31051	0.01964
H	-0.3307	0.01214	-1.86508
H	1.14759	1.35966	-1.41022
H	-0.93173	2.05724	-0.58024
H	2.91412	0.85939	0.73113
H	2.29074	-1.39738	0.98178
H	1.15724	-1.90733	-1.45772
H	1.39723	2.50376	-0.10218
H	-1.27141	-1.39032	-1.42004
H	-0.52046	1.75918	1.1047
N	-1.05058	0.07145	0.04715
C	-0.77633	-0.74779	1.23649
H	-1.12619	-0.23751	2.10948
H	-1.28047	-1.68727	1.14643
H	0.2773	-0.9149	1.31926
O	-2.3855	0.31311	-0.04871

1-methyl-2,3,6,7-tetrahydro-1H-azepine N-oxide
The number of imaginary frequencies: 0
Total Energy (M06/6-311G+**)= -404.25593024
ZPE=-404.061382

Enthalpy= -404.05197
Gibb’s Free Energy=-404.093798

C	-2.13243	-0.62773	-0.00619
C	-2.1129	0.69917	0.0899
C	-0.89012	1.57286	0.06504
C	-0.93812	-1.53555	-0.02644
C	0.26186	1.00473	-0.74248
C	0.2522	-1.00399	0.73689
H	-0.57255	1.84981	1.08181
H	-0.10086	0.52604	-1.65426
H	-0.61122	-1.78372	-1.04644
H	0.94894	-1.80485	0.99417
H	-3.1038	-1.11763	-0.04322
H	-3.06576	1.22109	0.15518
H	-1.16244	2.52684	-0.4037
H	-1.22239	-2.49315	0.42425
H	0.97609	1.77506	-1.04426
H	-0.06828	-0.49279	1.65335
N	1.10204	-0.06467	-0.08457
C	2.08847	0.57282	0.8329
H	2.6448	-0.23081	1.31627
H	2.76704	1.15301	0.20788
H	1.59677	1.20693	1.57869
O	1.76069	-0.74713	-1.04068

Triplet Oxygen
The number of imaginary frequencies: 0
Total Energy (B3LYP/6-31G*)= -75.06062312
ZPE= -75.060623
Enthalpy= -75.058263
Gibb’s Free Energy= -75.075575
O 0. 0. 0.

Triplet Oxygen
The number of imaginary frequencies: 0
Total Energy (B3LYP/6-31G**)= -75.06062312
ZPE= -75.060623
Enthalpy= -75.058263
Gibb’s Free Energy= -75.075575
O 0. 0. 0.

Triplet Oxygen
The number of imaginary frequencies: 0
Total Energy (M06/6-311G+**)= -75.05344297
ZPE= -75.053443
Enthalpy= -75.051083
Gibb’s Free Energy= -75.068395
O 0. 0. 0.

Singlet Oxygen
The number of imaginary frequencies: 0
Total Energy (B3LYP/6-31G*)= -74.95739789
ZPE= -74.957398
Enthalpy= -74.955037
Gibb’s Free Energy= -74.971313
O 0. 0. 0.

Singlet Oxygen
The number of imaginary frequencies: 0
Total Energy (B3LYP/6-31G**)= -74.95739789
ZPE= -74.957398
Enthalpy= -74.955037
Gibb’s Free Energy= -74.971313
O 0. 0. 0.

Singlet Oxygen
The number of imaginary frequencies: 0
Total Energy (M06/6-311G+**)= -74.95543188
ZPE= -74.955432
Enthalpy= -74.953071
Gibb’s Free Energy= -74.969347
O 0. 0. 0.

7-methyl-7-azanorcaradiene
The number of imaginary frequencies: 0
Total Energy (B3LYP/6-31G*)=-326.83454058
ZPE= -326.689984
Enthalpy=-326.682268
Gibb’s Free Energy= -326.720491

C	1.96758	-0.78106	-0.2494
C	1.9667	0.61802	-0.34996
C	0.88802	1.40813	-0.02297
C	-0.41571	0.93722	0.70222
C	-0.35664	-0.57731	0.48024
C	0.86155	-1.41833	0.14091
H	2.85043	-1.33594	-0.48935
H	2.85186	1.09785	-0.71211
H	0.94559	2.44192	-0.29287
H	0.83621	-2.48521	0.21865
H	-0.39413	0.92431	1.77192
H	-0.82068	-1.13466	1.26695
N	-1.35975	0.10157	-0.24957
C	-2.7971	-0.13006	-0.45273
H	-3.23944	0.73849	-0.89414
H	-2.93485	-0.96948	-1.1018
H	-3.26323	-0.3278	0.48988

7-methyl-7-azanorcaradiene
The number of imaginary frequencies: 0
Total Energy (B3LYP/6-31G**)= -326.84784237
ZPE= -326.703582
Enthalpy= -326.695868
Gibb’s Free Energy= -326.734078

C	1.86658	-0.72695	-0.30896
C	1.86652	0.72697	-0.30892
C	0.82595	1.43776	0.17935
C	-0.43635	0.77203	0.53789
C	-0.43622	-0.77204	0.53792
C	0.82604	-1.43774	0.17938
H	2.78195	-1.23639	-0.59808
H	2.78187	1.23649	-0.59798
H	0.91474	2.50678	0.35361
H	0.91476	-2.50677	0.35368
H	-1.07173	1.2685	1.2729
H	-1.07168	-1.26843	1.27288
N	-1.12942	0.00003	-0.49691
C	-2.58389	-0.00006	-0.38983
H	-2.97899	0.88754	-0.89449
H	-2.97884	-0.88773	-0.89446
H	-2.95796	-0.00007	0.64929

7-methyl-7-azanorcaradiene
The number of imaginary frequencies: 0
Total Energy (M06/6-311G+**)= -326.6688665
ZPE= -326.525862
Enthalpy= -326.51812
Gibb’s Free Energy= -326.556372

C	1.86736	-0.72717	-0.30848
C	1.86727	0.72699	-0.3085
C	0.82599	1.43804	0.17889
C	-0.43647	0.77206	0.53738
C	-0.43635	-0.77192	0.53743
C	0.82597	-1.43801	0.179
H	2.78349	-1.23668	-0.59757
H	2.78347	1.23657	-0.5974
H	0.91428	2.50789	0.35285
H	0.91386	-2.5079	0.35303
H	-1.07253	1.26895	1.27227
H	-1.07282	-1.26867	1.272
N	-1.13026	0.0002	-0.49695
C	-2.58436	-0.00015	-0.38897
H	-2.97867	0.88861	-0.89404
H	-2.97838	-0.88884	-0.89439
H	-2.95739	-0.0004	0.65145

7-methyl-7-azanorcaradiene N-oxide
The number of imaginary frequencies: 0
Total Energy (B3LYP/6-31G*)= -401.97620874
ZPE= -401.828039
Enthalpy=-401.81928
Gibb’s Free Energy= -401.859745

C	1.90702	-0.76695	-0.26941
C	1.92231	0.77444	-0.25264
C	0.86515	1.47579	0.22253
C	-0.47927	0.78862	0.52423
C	-0.47307	-0.74424	0.56603
C	0.82831	-1.46426	0.16868
H	2.76561	-1.29679	-0.62576
H	2.78738	1.29707	-0.60395
H	0.96946	2.52581	0.39994
H	0.87985	-2.53127	0.22968
H	-0.99883	1.39917	1.23287
H	-1.02755	-1.30025	1.29287
N	-1.19431	-0.01676	-0.49064
C	-2.6622	-0.01992	-0.41196
H	-3.0487	0.83602	-0.92467
H	-3.0419	-0.91034	-0.86789
H	-2.96445	0.01226	0.61396
O	-0.81195	-0.0583	-1.79513

7-methyl-7-azanorcaradiene N-oxide
The number of imaginary frequencies: 0
Total Energy (B3LYP/6-31G**)= -401.98966351
ZPE= -401.841848
Enthalpy= -401.83308
Gibb’s Free Energy= -401.873557

C	1.90489	0.72696	0.28203
C	1.90523	-0.72705	0.28062
C	0.94617	-1.43689	-0.35466
C	-0.25337	-0.76724	-0.84109
C	-0.2525	0.76857	-0.84149
C	0.94613	1.43748	-0.35318
H	2.76352	1.23737	0.70874
H	2.76448	-1.23784	0.70567
H	1.02988	-2.51044	-0.48731
H	1.02931	2.51139	-0.48328
H	-0.87801	-1.28311	-1.56504
H	-0.87785	1.28469	-1.56453
N	-1.06841	-0.00086	0.23813
C	-2.53029	0.00055	0.01848
H	-2.92878	-0.89135	0.50462

H	-2.92741	0.89181	0.50682
H	-2.77843	0.00199	-1.04642
O	-0.71442	-0.00159	1.48871

7-methyl-7-azanorcaradiene N-oxide
The number of imaginary frequencies: 0
Total Energy (M06/6-311G+**)= -401.80644197
ZPE= -401.659005
Enthalpy= -401.650406
Gibb's Free Energy= -401.690561

C	-1.9366	-0.69675	0.32594
C	-1.91983	0.72422	0.30748
C	-0.97434	1.43326	-0.37774
C	0.29984	0.74459	-0.87686
C	0.29666	-0.79492	-0.8178
C	-0.99917	-1.44498	-0.32655
H	-2.72311	-1.19699	0.85135
H	-2.68794	1.25638	0.82873
H	-1.12616	2.47491	-0.56963
H	-1.17031	-2.48784	-0.49408
H	0.79835	1.24075	-1.68322
H	0.73905	-1.36195	-1.61006
N	1.09013	-0.00025	0.11397
C	2.56009	-0.00873	0.1194
H	2.92013	0.87937	0.59538
H	2.90984	-0.86691	0.65429
H	2.92028	-0.04482	-0.88751
O	0.72863	0.04609	1.42422

Pyridine
The number of imaginary frequencies: 0
Total Energy (B3LYP/6-31G**)= -248.29259287
ZPE= -248.203718
Enthalpy= -248.198501
Gibb's Free Energy= -248.231119 C
-1.14249 -0.72173 -0.00022

C	-1.19869	0.67323	-0.00012
C	0.0002	1.38545	0.0001
C	1.19888	0.67291	0.00022
C	1.14229	-0.72204	0.00013
N	-0.0002	-1.42093	-0.0001
H	0.00032	2.47233	0.00016
H	-2.05988	-1.30864	-0.00037
H	-2.15775	1.18284	-0.00023
H	2.1581	1.18221	0.00036
H	2.05951	-1.3092	0.00019

Pyridine N-oxide
The number of imaginary frequencies: 0
Total Energy (B3LYP/6-31G**)= -323.45500559
ZPE= -323.361911
Enthalpy= -323.355908
Gibb's Free Energy= -323.390607

C	0.28383	-1.18039	-0.00018
C	-1.09931	-1.19458	-0.00009
C	-1.82222	0.	0.00012
C	-1.09931	1.19458	0.00024
C	0.28383	1.18039	0.00014
N	0.98713	0.	-0.00007
H	-2.90654	0.	0.0002
H	0.91433	-2.05913	-0.00034
H	-1.60303	-2.15629	-0.00019
H	-1.60303	2.15629	0.00041
H	0.91433	2.05913	0.00022
O	2.26163	0.	-0.00015

Trimethylamine
The number of imaginary frequencies: 0
Total Energy (B3LYP/6-31G**)= -174.4861591
ZPE= -174.365595
Enthalpy= -174.359219
Gibb's Free Energy= -174.391860

N	0.	0.	0.37142
C	0.	1.38908	-0.06099
H	-0.88689	1.89889	0.33175
H	0.88689	1.89889	0.33175
H	0.	1.5053	-1.16421
C	-1.20298	-0.69454	-0.06099
H	-1.20104	-1.71751	0.33175
H	-2.08793	-0.18138	0.33175
H	-1.30363	-0.75265	-1.16421
C	1.20298	-0.69454	-0.06099
H	2.08793	-0.18138	0.33175
H	1.20104	-1.71751	0.33175
H	1.30363	-0.75265	-1.16421

Trimethylamine N-oxide
The number of imaginary frequencies: 0
Total Energy (B3LYP/6-31G**)= -249.62754733
ZPE= -249.502133
Enthalpy= -249.495143
Gibb's Free Energy= -249.529500

N	0.	0.	0.08312
C	0.	1.41754	-0.41881

H	-0.88839	1.89255	-0.0028
H	0.88839	1.89255	-0.0028
H	0.	1.4678	-1.51383
C	-1.22763	-0.70877	-0.41881
H	-1.1948	-1.71564	-0.0028
H	-2.08319	-0.1769	-0.0028
H	-1.27115	-0.7339	-1.51383
C	1.22763	-0.70877	-0.41881
H	2.08319	-0.1769	-0.0028
H	1.1948	-1.71564	-0.0028
H	1.27115	-0.7339	-1.51383
O	0.	0.	1.43938

N-methylaziridine
The number of imaginary frequencies: 0
Total Energy (B3LYP/6-31G*)= -173.23135212
ZPE= -173.13292
Enthalpy= -173.127558
Gibb's Free Energy= -173.159205

C	-0.81263	0.01988	-0.00942
C	0.69188	0.01646	0.01901
H	-1.31927	-0.34205	-0.8796
H	-1.36422	-0.28283	0.85604
H	1.30576	-0.2704	-0.80909
H	1.16767	-0.2429	0.94165
N	-0.06563	1.32111	-0.03859
C	-0.13378	2.19462	-1.21895
H	-1.01422	2.80019	-1.16415
H	0.73094	2.82418	-1.24716
H	-0.16768	1.59529	-2.1047

N-methylaziridine N-oxide
The number of imaginary frequencies: 0
Total Energy (B3LYP/6-31G*)= -248.36984065
ZPE= -248.267481
Enthalpy= -248.261373
Gibb's Free Energy= -248.295118

C	0.91154	0.77392	0.11511
C	0.89824	-0.77227	0.10721
H	0.7465	1.28391	1.04117
H	1.58558	1.27929	-0.54457
H	0.78061	-1.29457	1.03364
H	1.59987	-1.28788	-0.51468
N	-0.20087	0.00088	-0.47225
C	-1.55546	0.00289	0.09869
H	-2.07808	0.87829	-0.22604
H	-2.08198	-0.86901	-0.22913
H	-1.49231	0.00086	1.16682
O	-0.38148	0.00368	-1.8202

N-methylaziridine
The number of imaginary frequencies: 0
Total Energy (B3LYP/6-31G**)= -173.23135212
ZPE= -173.13292
Enthalpy= -173.127558
Gibb's Free Energy= -173.159205

C	0.9011	0.74593	0.12103
C	0.90182	-0.74557	0.12108
H	0.65933	1.24157	1.06284
H	1.57628	1.30308	-0.52521
H	0.66068	-1.24143	1.06294
H	1.57806	-1.30166	-0.52499
N	-0.1564	-0.00029	-0.55436
C	-1.45929	-0.00019	0.09732
H	-2.01925	0.88759	-0.21681
H	-2.0191	-0.88812	-0.2166
H	-1.40297	0.00001	1.20182

N-methylaziridine N-oxide
The number of imaginary frequencies: 0
Total Energy (B3LYP/6-31G**)= -248.36984065
ZPE= -248.267481
Enthalpy= -248.261373
Gibb's Free Energy= -248.295118

C	0.91197	0.77358	0.11543
C	0.89812	-0.77261	0.10775
H	0.74695	1.28376	1.04138
H	1.58629	1.27861	-0.54422
H	0.78015	-1.29473	1.03424
H	1.59968	-1.28856	-0.51395
N	-0.20061	0.00085	-0.47201
C	-1.5553	0.00342	0.0987
H	-2.07756	0.87895	-0.22625
H	-2.08207	-0.86834	-0.22908
H	-1.49234	0.00152	1.16684
O	-0.38099	0.0035	-1.81999

N-methylaziridine
The number of imaginary frequencies: 0
Total Energy (M06/6-311G+**)= -173.14757437
ZPE= -173.050266
Enthalpy= -173.044925
Gibb's Free Energy= -173.076507

C	-0.93158	-0.75352	0.1074
C	-0.93188	0.74813	0.12172
H	-0.69467	-1.35042	0.96325

H	-1.68532	-1.25779	-0.46048
H	-0.82027	1.35583	0.99531
H	-1.61782	1.23386	-0.54044
N	0.19908	0.00654	-0.54255
C	1.51627	0.00101	0.11005
H	2.06081	-0.86878	-0.193
H	2.05878	0.87842	-0.1741
H	1.388	-0.01062	1.17227

N-methylaziridine N-oxide

The number of imaginary frequencies: 0

Total Energy (M06/6-311G+**)= -248.28292193

ZPE= -248.181688

Enthalpy= -248.175647

Gibb's Free Energy= -248.209266

C	0.89389	0.78517	0.1286
C	0.91063	-0.76037	0.13715
H	0.78837	1.31758	1.05072
H	1.55257	1.28652	-0.5494
H	0.69589	-1.26128	1.05795
H	1.65022	-1.27839	-0.43692
N	-0.19019	-0.00268	-0.47178
C	-1.55579	-0.0167	0.07213
H	-2.08411	0.84935	-0.26804
H	-2.06334	-0.89781	-0.26095
H	-1.51393	-0.01187	1.1413
O	-0.34374	-0.00959	-1.82307

Cartesian Coordinates (Å) and energies (Hartrees) for all structures calculated using Gaussian 16.

N-methylazepine N-oxide

The number of imaginary frequencies: 0

B3LYP/6-31G**

Enthalpy = -401.987790971

6	1.969668	-0.680418	0.208142
6	1.969400	0.680840	0.208107
6	0.928373	1.505631	-0.354441
6	0.928837	-1.505655	-0.354106
6	-0.368340	1.197552	-0.538867
6	-0.368016	-1.198005	-0.538327
1	2.865300	-1.192417	0.550726
1	2.864894	1.193165	0.550561
1	1.229467	2.487553	-0.714713
1	1.230211	-2.487594	-0.714117
1	-1.084432	1.813237	-1.067255
1	-1.084025	-1.814112	-1.066334
7	-1.072525	-0.000194	-0.007446
8	-2.358751	-0.000265	-0.468970
6	-1.098760	0.000428	1.498914
1	-1.659893	-0.889641	1.780260
1	-0.094888	-0.001947	1.928068
1	-1.655926	0.893004	1.780144

N-methylazepine N-oxide

The number of imaginary frequencies: 0

APFD/aug-cc-pVDZ

Enthalpy = -401.683840334

6	0.267783	-0.765912	-0.850829
6	0.266149	0.768828	-0.851379
1	-1.024147	-2.511770	-0.522626
6	-0.930445	-1.434678	-0.374622
6	-0.930346	1.435990	-0.371472
6	-1.873434	0.725588	0.290486
6	-1.874281	-0.725756	0.287379
1	-1.023187	2.513828	-0.514689
1	-2.730252	1.239817	0.732943
1	-2.732553	-1.240742	0.726194
7	1.044658	-0.001765	0.246063
6	2.499037	0.001073	0.057002
1	2.886681	0.896917	0.558671
1	2.889451	-0.896205	0.554123
1	2.772801	0.004022	-1.007715
1	0.911605	-1.285576	-1.565411
1	0.911128	1.288788	-1.564505
8	0.659887	-0.003440	1.470147

N-methylazepine N-oxide

The number of imaginary frequencies: 0

APFD/aug-cc-pVTZ

Enthalpy = -401.788822197

6	1.936336	-0.677553	0.211900
6	1.936345	0.677535	0.211970
6	0.910417	1.491864	-0.369788
6	0.910389	-1.491833	-0.369887
6	-0.376347	1.175332	-0.561500
6	-0.376385	-1.175288	-0.561548
1	2.827436	-1.187874	0.561969
1	2.827444	1.187808	0.562110
1	1.212970	2.464927	-0.745423
1	1.212936	-2.464858	-0.745620
1	-1.089698	1.768307	-1.115084
1	-1.089730	-1.768214	-1.115195
7	-1.066001	0.000025	0.000727
8	-2.357261	0.000062	-0.397984

6 -1.018836 -0.000100 1.490928
1 -1.558371 -0.890416 1.801015
1 0.004805 0.000220 1.861470
1 -1.559211 0.889686 1.801089

1-methyl-2,3,4,5-tetrahydro-1H-azepine N-oxide
The number of imaginary frequencies: 0
B3LYP/6-31G**
Enthalpy= -404.439377914
6 2.023212 -0.761124 0.181705
6 1.777779 0.725632 0.493583
6 0.968236 1.412198 -0.618470
6 0.889170 -1.523008 -0.448140
6 -0.551882 1.280359 -0.480836
6 -0.405432 -1.224603 -0.528919
1 2.877818 -0.848634 -0.504799
1 2.750829 1.219588 0.596870
1 1.178119 2.488396 -0.631154
1 1.166901 -2.471881 -0.905311
1 -1.034354 1.436125 -1.452725
7 -1.047619 -0.042198 0.106373
8 -0.851457 -0.062281 1.463086
6 -2.530440 -0.120374 -0.136997
1 -2.769823 -0.148817 -1.205644
1 -2.872303 -1.020394 0.371731
1 -2.970022 0.752761 0.341584
1 -1.109400 -1.896943 -1.006396
1 2.327864 -1.292376 1.094489
1 1.233461 0.813478 1.434658
1 1.286555 1.030340 -1.596322
1 -0.944518 1.997513 0.242163

1-methyl-2,3,4,5-tetrahydro-1H-azepine N-oxide
The number of imaginary frequencies: 0
APFD/aug-cc-pVDZ
Enthalpy= -404.123157784
6 1.941457 -0.789644 0.253272
6 1.779678 0.726556 0.468894
6 0.962522 1.389394 -0.641095
6 0.859247 -1.497314 -0.506966
6 -0.548692 1.281066 -0.459394
6 -0.430196 -1.180479 -0.608579
1 2.884612 -0.981306 -0.288916
1 2.784230 1.180227 0.502029
1 1.188853 2.467696 -0.692664
1 1.157943 -2.407691 -1.037976
1 -1.066516 1.463152 -1.415800
7 -1.031542 -0.038921 0.113207
8 -0.752767 -0.110983 1.433507
6 -2.511481 -0.098481 -0.054054
1 -2.808945 -0.080569 -1.114444
1 -2.834200 -1.022026 0.438811
1 -2.921344 0.760541 0.487075
1 -1.134485 -1.782349 -1.184919
1 2.050641 -1.296140 1.229102
1 1.281831 0.907106 1.430062
1 1.253863 0.970370 -1.620094
1 -0.908765 1.994716 0.294767

1-methyl-2,3,4,5-tetrahydro-1H-azepine N-oxide
The number of imaginary frequencies: 0
APFD/aug-cc-pVTZ
Enthalpy= -404.240506936
6 1.978245 -0.771779 0.209821
6 1.777074 0.723720 0.477927
6 0.963680 1.392242 -0.627109
6 0.866795 -1.501145 -0.473464
6 -0.544562 1.273814 -0.456823
6 -0.417313 -1.195643 -0.560395
1 2.869416 -0.906540 -0.414722
1 2.761400 1.195441 0.543579
1 1.183005 2.463067 -0.668433
1 1.147926 -2.429522 -0.963314
1 -1.047170 1.443774 -1.412615
7 -1.037342 -0.040732 0.113352
8 -0.812266 -0.095833 1.449193
6 -2.507697 -0.100082 -0.110604
1 -2.752460 -0.105179 -1.175110
1 -2.855852 -1.004500 0.380636
1 -2.935467 0.765894 0.386018
1 -1.120898 -1.834517 -1.076943
1 2.198355 -1.295440 1.147590
1 1.267225 0.860499 1.430010
1 1.259835 0.983476 -1.598280
1 -0.913122 1.988571 0.278456

1-methyl-2,3,6,7-tetrahydro-1H-azepine N-oxide
The number of imaginary frequencies: 0
B3LYP/6-31G**
Enthalpy= -404.433978813
6 -0.576807 1.842742 0.666223
6 -0.576807 1.842742 -0.666223
6 0.508217 1.117225 -1.423688
6 0.508217 1.117225 1.423688
6 0.508217 -0.419616 -1.292730
6 0.508217 -0.419616 1.292730
1 0.449230 1.341231 -2.493823

1 1.525868 -0.802219 -1.437872
7 -0.065214 -1.005938 -0.000000
8 -1.418000 -0.893579 -0.000000
6 0.277768 -2.475051 -0.000000
1 1.359134 -2.651960 -0.000000
1 -0.190055 -2.900739 0.886380
1 -0.190055 -2.900739 -0.886380
1 1.525868 -0.802219 1.437872
1 -1.366336 2.335326 1.225810
1 -1.366336 2.335326 -1.225810
1 1.485460 1.495474 -1.093683
1 -0.154553 -0.864943 -2.034635
1 0.449230 1.341231 2.493823
1 -0.154553 -0.864943 2.034635
1 1.485460 1.495474 1.093683

1-methyl-2,3,6,7-tetrahydro-1H-azepine N-oxide
The number of imaginary frequencies: 0
APFD/aug-cc-pVDZ
Enthalpy=-404.118296570
6 -0.580349 1.816900 0.667109
6 -0.580349 1.816900 -0.667109
6 0.516659 1.107151 -1.410951
6 0.516659 1.107151 1.410951
6 0.516659 -0.422673 -1.278441
6 0.516659 -0.422673 1.278441
1 0.478492 1.336464 -2.487119
1 1.540076 -0.813529 -1.409237
7 -0.070970 -0.994026 -0.000000
8 -1.403662 -0.848300 -0.000000
6 0.245188 -2.455492 -0.000000
1 1.329340 -2.652048 -0.000000
1 -0.233266 -2.876859 0.890642
1 -0.233266 -2.876859 -0.890642
1 1.540076 -0.813529 1.409237
1 -1.388686 2.290435 1.228873
1 -1.388686 2.290435 -1.228873
1 1.490200 1.493395 -1.057620
1 -0.141820 -0.873389 -2.031483
1 0.478492 1.336464 2.487119
1 -0.141820 -0.873389 2.031483
1 1.490200 1.493395 1.057620

1-methyl-2,3,6,7-tetrahydro-1H-azepine N-oxide
The number of imaginary frequencies: 0
APFD/aug-cc-pVTZ
Enthalpy=-404.236049675
6 -0.574719 1.823334 0.662558
6 -0.574719 1.823334 -0.662558
6 0.510721 1.106581 -1.409081
6 0.510721 1.106581 1.409081
6 0.510721 -0.421084 -1.273461
6 0.510721 -0.421084 1.273461
1 0.463560 1.331838 -2.476481
1 1.528360 -0.803455 -1.394617
7 -0.071517 -0.995877 -0.000000
8 -1.410929 -0.869542 0.000000
6 0.259926 -2.450388 -0.000000
1 1.338205 -2.625857 -0.000000
1 -0.206064 -2.876044 0.884313
1 -0.206064 -2.876044 -0.884313
1 1.528360 -0.803455 1.394617
1 -1.369087 2.304794 1.219809
1 -1.369087 2.304794 -1.219809
1 1.479264 1.487992 -1.065618
1 -0.131227 -0.870277 -2.028562
1 0.463560 1.331838 2.476481
1 -0.131227 -0.870277 2.028562
1 1.479264 1.487992 1.065618

1-methyl-2,3,4,7-tetrahydro-1H-azepine N-oxide
The number of imaginary frequencies: 0
B3LYP/6-31G**
Enthalpy= -404.444680715
6 -1.958168 0.798791 0.156899
6 -1.884165 -0.677640 0.437836
6 -1.005620 -1.427777 -0.577975
6 -0.979128 1.559437 -0.336153
6 0.502697 -1.340071 -0.351742
6 0.427614 1.166537 -0.695513
1 -2.896859 -1.096736 0.403419
1 -1.252424 -2.496320 -0.561356
1 1.032607 -1.620590 -1.270068
7 1.016583 0.015684 0.124606
8 0.769016 0.175018 1.446679
6 2.509277 0.029320 -0.083156
1 2.780036 -0.002802 -1.144270
1 2.871169 0.938979 0.392858
1 2.909483 -0.834453 0.446194
1 0.532992 0.920207 -1.761754
1 -2.901162 1.289462 0.392927
1 -1.489531 -0.825230 1.449740
1 -1.255107 -1.074703 -1.586040
1 0.817881 -1.993295 0.462973
1 -1.176692 2.619443 -0.484723
1 1.084359 2.014525 -0.486750

1-methyl-2,3,4,7-tetrahydro-1H-azepine N-oxide
The number of imaginary frequencies: 0

APFD/aug-cc-pVDZ
Enthalpy= -404.129255205
6 -1.943774 0.792334 0.160870
6 -1.864537 -0.678237 0.440491
6 -0.997440 -1.416429 -0.583048
6 -0.967470 1.553729 -0.340857
6 0.504767 -1.327512 -0.356899
6 0.432762 1.153455 -0.697393
1 -2.882164 -1.102341 0.421575
1 -1.245076 -2.491298 -0.582848
1 1.043561 -1.600439 -1.279999
7 1.007662 0.015371 0.129391
8 0.738792 0.173323 1.430003
6 2.488691 0.030164 -0.062706
1 2.770414 0.012281 -1.127500
1 2.848037 0.938204 0.432983
1 2.887481 -0.844567 0.462825
1 0.536299 0.894306 -1.767247
1 -2.890611 1.288014 0.402619
1 -1.450380 -0.820152 1.453266
1 -1.252459 -1.046847 -1.591906
1 0.822725 -1.990523 0.458506
1 -1.168570 2.619345 -0.492161
1 1.098777 2.004814 -0.498624

1-methyl-2,3,4,7-tetrahydro-1H-azepine N-oxide
The number of imaginary frequencies: 0
APFD/aug-cc-pVTZ
Enthalpy= -404.246538534
6 -1.937752 0.796618 0.159803
6 -1.877343 -0.673056 0.427883
6 -0.995392 -1.413871 -0.575379
6 -0.964637 1.545607 -0.342658
6 0.499574 -1.331134 -0.323577
6 0.426871 1.135744 -0.707140
1 -2.890669 -1.082608 0.379541
1 -1.246806 -2.478591 -0.577394
1 1.044330 -1.624119 -1.225637
7 1.009679 0.016386 0.131013
8 0.760071 0.200090 1.436158
6 2.484110 0.024091 -0.082977
1 2.739314 -0.012614 -1.144578
1 2.855564 0.932333 0.382703
1 2.887891 -0.837101 0.442810
1 0.515419 0.851957 -1.762379
1 -2.869250 1.295662 0.412803
1 -1.503932 -0.830847 1.444706
1 -1.227361 -1.045564 -1.579855
1 0.796683 -1.977416 0.500914
1 -1.153116 2.606147 -0.478459
1 1.091029 1.983349 -0.537259

N-methylazepane N-oxide
The number of imaginary frequencies: 0
B3LYP/6-31G**
Enthalpy = -405.675354659
6 -0.607085 1.792740 0.777922
6 -0.607085 1.792740 -0.777922
6 0.568106 1.038282 -1.419616
6 0.568106 1.038282 1.419616
6 0.568106 -0.490602 -1.278837
6 0.568106 -0.490602 1.278837
1 -0.562496 2.826694 1.142109
1 -0.562496 2.826694 -1.142109
1 0.568826 1.238510 -2.498135
1 1.516849 1.443877 1.051641
1 1.587025 -0.881026 -1.390306
1 -0.069590 -0.940647 2.040868
7 -0.037297 -1.055132 -0.000000
8 -1.387282 -0.860707 -0.000000
6 0.213796 -2.540083 -0.000000
1 1.282444 -2.779776 -0.000000
1 -0.277798 -2.937635 0.886526
1 -0.277798 -2.937635 -0.886526
1 1.587025 -0.881026 1.390306
1 0.568826 1.238510 2.498135
1 -1.540526 1.353638 1.131392
1 -1.540526 1.353638 -1.131392
1 1.516849 1.443877 -1.051641
1 -0.069590 -0.940647 -2.040868

N-methylazepane N-oxide
The number of imaginary frequencies: 0
APFD/aug-cc-pVDZ
Enthalpy = -405.357516692
6 -0.608566 1.775840 0.774611
6 -0.608566 1.775840 -0.774611
6 0.569551 1.031910 -1.406300
6 0.569551 1.031910 1.406300
6 0.569551 -0.490908 -1.264864
6 0.569551 -0.490908 1.264864
1 -0.572153 2.815685 1.142885
1 -0.572153 2.815685 -1.142885
1 0.584187 1.234582 -2.490714
1 1.519222 1.442856 1.026234
1 1.594027 -0.887282 -1.366734
1 -0.065050 -0.946299 2.036392
7 -0.042627 -1.047369 0.000000
8 -1.373301 -0.839667 -0.000000
6 0.200612 -2.520014 0.000000
1 1.273957 -2.766223 0.000000
1 -0.296638 -2.918428 0.890819
1 -0.296638 -2.918428 -0.890819
1 1.594027 -0.887282 1.366734

1	0.584187	1.234582	2.490714
1	-1.543225	1.325445	1.133449
1	-1.543225	1.325445	-1.133449
1	1.519222	1.442856	-1.026234
1	-0.065050	-0.946299	-2.036392

N-methylazepane N-oxide
The number of imaginary frequencies: 0
APFD/aug-cc-pVTZ
Enthalpy = -405.475774305

6	-0.608880	1.778496	0.773162
6	-0.608880	1.778496	-0.773162
6	0.564014	1.032637	-1.404527
6	0.564014	1.032637	1.404527
6	0.564014	-0.487644	-1.261500
6	0.564014	-0.487644	1.261500
1	-0.571183	2.809741	1.137832
1	-0.571183	2.809741	-1.137832
1	0.574914	1.233755	-2.480047
1	1.505771	1.438754	1.027194
1	1.582187	-0.875613	-1.357279
1	-0.058439	-0.939919	2.031967
7	-0.041192	-1.048597	0.000000
8	-1.378162	-0.860724	0.000000
6	0.221575	-2.514703	0.000000
1	1.290643	-2.737348	0.000000
1	-0.262589	-2.919327	0.884357
1	-0.262589	-2.919327	-0.884357
1	1.582187	-0.875613	1.357279
1	0.574914	1.233755	2.480047
1	-1.538770	1.337450	1.128002
1	-1.538770	1.337450	-1.128002
1	1.505771	1.438754	-1.027194
1	-0.058439	-0.939919	-2.031967

N-methylazepine
The number of imaginary frequencies: 0
B3LYP/6-31G**
Enthalpy = -326.859656161

6	-0.609487	-1.771782	0.676167
6	-0.609487	-1.771782	-0.676167
6	-0.609487	-0.579396	-1.518592
6	-0.609487	-0.579396	1.518592
6	-0.053329	0.600352	-1.193902
6	-0.053329	0.600352	1.193902
1	-0.689963	-2.728232	1.190057
1	-0.689963	-2.728232	-1.190057
1	-1.123176	-0.641556	-2.475142
1	-1.123176	-0.641556	2.475142
1	-0.151617	1.457795	-1.862488
1	-0.151617	1.457795	1.862488
7	0.681491	0.827495	0.000000
6	1.523504	2.013041	0.000000
1	0.950836	2.958234	0.000000
1	2.167430	2.002472	0.883203
1	2.167430	2.002472	-0.883203

N-methylazepine
The number of imaginary frequencies: 0
APFD/aug-cc-pVDZ
Enthalpy = -326.594812304

6	-0.634887	-1.747142	0.677373
6	-0.634887	-1.747142	-0.677373
6	-0.634887	-0.554397	-1.512618
6	-0.634887	-0.554397	1.512618
6	-0.050612	0.611857	-1.181001
6	-0.050612	0.611857	1.181001
1	-0.727264	-2.709068	1.193506
1	-0.727264	-2.709068	-1.193506
1	-1.168036	-0.600160	-2.466926
1	-1.168036	-0.600160	2.466926
1	-0.142786	1.489207	-1.836567
1	-0.142786	1.489207	1.836567
7	0.706137	0.791799	0.000000
6	1.580894	1.940897	0.000000
1	1.035579	2.909451	0.000000
1	2.228447	1.909404	0.887501
1	2.228447	1.909404	-0.887501

N-methylazepine
The number of imaginary frequencies: 0
APFD/zug-cc-pVTZ
Enthalpy = -326.682346079

6	-0.621889	-1.751999	0.672556
6	-0.621889	-1.751999	-0.672556
6	-0.621889	-0.563235	-1.506411
6	-0.621889	-0.563235	1.506411
6	-0.045794	0.598633	-1.181834
6	-0.045794	0.598633	1.181834
1	-0.705900	-2.706045	1.185584
1	-0.705900	-2.706045	-1.185584
1	-1.139456	-0.614618	-2.458628
1	-1.139456	-0.614618	2.458628
1	-0.127540	1.459628	-1.844287
1	-0.127540	1.459628	1.844287
7	0.693358	0.805277	0.000000
6	1.539113	1.974540	0.000000
1	0.970210	2.917998	0.000000
1	2.181132	1.959552	0.880610
1	2.181132	1.959552	-0.880610

1-methyl-2,3,4,5-tetrahydro-1H-azepine
The number of imaginary frequencies: 0
B3LYP/6-31G**
Enthalpy= -329.310375943

6	-1.660774	-0.814901	-0.590395
6	-2.050443	0.546084	0.020195
6	-0.847314	1.381365	0.510666
6	-0.607181	-1.584990	0.176633
6	0.455989	1.204551	-0.281741
6	0.652078	-1.179921	0.419891
1	-2.561653	-1.430564	-0.686480
1	-2.731133	0.391165	0.865819
1	-1.121086	2.442831	0.493684
1	-0.875705	-2.567305	0.554317
1	1.085612	2.088935	-0.123744
7	1.244816	0.060230	0.179915
6	2.629155	0.050140	-0.259049
1	3.103870	1.009002	-0.022126
1	3.175884	-0.739000	0.266142
1	2.744132	-0.126581	-1.342437
1	1.333690	-1.868786	0.918603
1	-1.313274	-0.652734	-1.619447
1	-2.618875	1.113319	-0.728478
1	-0.622567	1.139710	1.554890
1	0.258342	1.164435	-1.367346

1-methyl-2,3,4,5-tetrahydro-1H-azepine
The number of imaginary frequencies: 0
APFD/aug-cc-pVDZ
Enthalpy= -329.040184702

6	-1.633891	-0.805465	-0.607611
6	-2.037924	0.542103	0.004637
6	-0.846602	1.361647	0.525568
6	-0.611450	-1.578749	0.186986
6	0.452563	1.204956	-0.264153
6	0.648827	-1.172085	0.432424
1	-2.534883	-1.422368	-0.753229
1	-2.743157	0.379817	0.837815
1	-1.122150	2.429166	0.544853
1	-0.894314	-2.555576	0.587196
1	1.087822	2.089626	-0.087390
7	1.235104	0.059056	0.173200
6	2.605490	0.049902	-0.272368
1	3.090609	1.009164	-0.028756
1	3.159006	-0.751845	0.239068
1	2.709858	-0.114779	-1.365143
1	1.334187	-1.856290	0.947418
1	-1.238966	-0.631742	-1.624588
1	-2.593302	1.122752	-0.753707
1	-0.621824	1.085202	1.569104
1	0.259301	1.189624	-1.357934

1-methyl-2,3,4,5-tetrahydro-1H-azepine
The number of imaginary frequencies: 0
APFD/aug-cc-pVTZ
Enthalpy= -329.132074646

6	-1.631766	-0.804752	-0.602114
6	-2.033773	0.543621	0.003461
6	-0.843468	1.355649	0.528558
6	-0.607016	-1.571976	0.188732
6	0.448144	1.201462	-0.268138
6	0.647505	-1.169017	0.426087
1	-2.525747	-1.418677	-0.735123
1	-2.737962	0.385343	0.825777
1	-1.115489	2.414309	0.558836
1	-0.884640	-2.543172	0.580897
1	1.079520	2.078817	-0.097631
7	1.229899	0.055877	0.162681
6	2.603797	0.053700	-0.264817
1	3.076679	1.007055	-0.015750
1	3.148743	-0.740833	0.247504
1	2.717513	-0.108027	-1.347490
1	1.328222	-1.849614	0.932174
1	-1.246927	-0.637754	-1.613791
1	-2.575874	1.121057	-0.753304
1	-0.620765	1.067129	1.559085
1	0.246905	1.181100	-1.350566

1-methyl-2,3,6,7-tetrahydro-1H-azepine
The number of imaginary frequencies: 0
B3LYP/6-31G**
Enthalpy= -329.298190209

6	-0.117334	-1.817960	0.668012
6	-0.117334	-1.817960	-0.668012
6	-0.795123	-0.702640	-1.426087
6	-0.795123	-0.702640	1.426087
6	-0.117334	0.685480	-1.243606
6	-0.117334	0.685480	1.243606
1	-0.837360	-0.923310	-2.497320
1	-0.887863	1.472632	-1.374158
7	0.631652	0.846287	-0.000000
6	1.381910	2.091312	-0.000000
1	0.743786	2.997527	-0.000000
1	2.024232	2.137182	0.885874
1	2.024232	2.137182	-0.885874
1	-0.887863	1.472632	1.374158
1	0.382770	-2.607309	1.224157
1	0.382770	-2.607309	-1.224157
1	-1.839519	-0.633339	-1.093264
1	0.608080	0.830160	-2.052764
1	-0.837360	-0.923310	2.497320
1	0.608080	0.830160	2.052764

1 -1.839519 -0.633339 1.093264

1-methyl-2,3,6,7-tetrahydro-1H-azepine
The number of imaginary frequencies: 0
APFD/aug-cc-pVDZ
Enthalpy= -329.028619850

6 -0.117214 -1.802277 0.669165
6 -0.117214 -1.802277 -0.669165
6 -0.803991 -0.690553 -1.413492
6 -0.803991 -0.690553 1.413492
6 -0.117214 0.684780 -1.232261
6 -0.117214 0.684780 1.232261
1 -0.871377 -0.908076 -2.490298
1 -0.882342 1.488555 -1.351028
7 0.635103 0.828533 -0.000000
6 1.394302 2.055214 -0.000000
1 0.760324 2.972453 -0.000000
1 2.040767 2.097090 0.890427
1 2.040767 2.097090 -0.890427
1 -0.882342 1.488555 1.351028
1 0.395616 -2.590891 1.227973
1 0.395616 -2.590891 -1.227973
1 -1.846947 -0.621006 -1.054953
1 0.608872 0.830894 -2.049088
1 -0.871377 -0.908076 2.490298
1 0.608872 0.830894 2.049088
1 -1.846947 -0.621006 1.054953

1-methyl-2,3,6,7-tetrahydro-1H-azepine
The number of imaginary frequencies: 0
APFD/aug-cc-pVTZ
Enthalpy= -329.120153118
6 -0.115721 -1.800088 0.664583
6 -0.115721 -1.800088 -0.664583
6 -0.799039 -0.692754 -1.410560
6 -0.799039 -0.692754 1.410560
6 -0.115721 0.681737 -1.231163
6 -0.115721 0.681737 1.231163
1 -0.859878 -0.911409 -2.478286
1 -0.878052 1.475381 -1.347917
7 0.631095 0.832311 0.000000
6 1.384059 2.060551 0.000000
1 0.747002 2.964152 0.000000
1 2.024250 2.104781 0.883534
1 2.024250 2.104781 -0.883534
1 -0.878052 1.475381 1.347917
1 0.392059 -2.582268 1.218953
1 0.392059 -2.582268 -1.218953
1 -1.834151 -0.623112 -1.058122
1 0.604144 0.826434 -2.041318
1 -0.859878 -0.911409 2.478286
1 0.604144 0.826434 2.041318
1 -1.834151 -0.623112 1.058122

1-methyl-2,3,4,7-tetrahydro-1H-azepine
The number of imaginary frequencies: 0
B3LYP/6-31G**
Enthalpy= -329.304274524
6 1.833298 0.796941 -0.311605
6 1.748059 -0.695644 -0.533662
6 0.866298 -1.402891 0.509448
6 0.867290 1.577518 0.179704
6 -0.642176 -1.269839 0.261397
6 -0.555417 1.179060 0.506237
1 2.762488 -1.109411 -0.480336
1 1.104056 -2.473073 0.539757
1 -1.178569 -1.523506 1.199666
7 -1.050247 0.032799 -0.259575
6 -2.492718 0.078889 -0.449834
1 -3.068776 0.001519 0.493455
1 -2.776431 1.012719 -0.945075
1 -2.803289 -0.750401 -1.094210
1 -0.677565 1.037558 1.601189
1 2.790792 1.261848 -0.542959
1 1.384903 -0.914766 -1.547568
1 1.121763 -1.005611 1.498725
1 -0.952149 -2.016047 -0.482058
1 1.091063 2.630467 0.347851
1 -1.194361 2.034910 0.258478

1-methyl-2,3,4,7-tetrahydro-1H-azepine
The number of imaginary frequencies: 0
APFD/aug-cc-pVDZ
Enthalpy= -329.034597535
6 1.823517 0.786564 -0.317485
6 1.719667 -0.698792 -0.545825
6 0.860971 -1.392823 0.515894
6 0.864605 1.570924 0.186838
6 -0.641458 -1.258127 0.275438
6 -0.551759 1.169588 0.508676
1 2.736338 -1.125749 -0.529289
1 1.101803 -2.467998 0.565011
1 -1.185867 -1.494335 1.221682
7 -1.034729 0.032347 -0.259505
6 -2.464660 0.082312 -0.461276
1 -3.049428 0.018332 0.485324
1 -2.744602 1.015251 -0.973467
1 -2.778119 -0.758139 -1.099903
1 -0.676766 1.018477 1.608760
1 2.788360 1.250660 -0.552126
1 1.318455 -0.906143 -1.555104
1 1.129319 -0.974515 1.501246

1 -0.961500 -2.015588 -0.461592
1 1.098782 2.626912 0.365175
1 -1.198967 2.028534 0.267271

1-methyl-2,3,4,7-tetrahydro-1H-azepine
The number of imaginary frequencies: 0
APFD/aug-cc-pVTZ
Enthalpy= -329.126037128
6 1.819980 0.784657 -0.316078
6 1.720924 -0.698150 -0.537649
6 0.855284 -1.387898 0.516836
6 0.866581 1.564342 0.182039
6 -0.641341 -1.259149 0.260372
6 -0.545663 1.165131 0.508275
1 2.730083 -1.119362 -0.509365
1 1.097178 -2.453102 0.572715
1 -1.188598 -1.502230 1.191995
7 -1.036478 0.034366 -0.258923
6 -2.465314 0.086886 -0.452967
1 -3.036045 0.015298 0.490714
1 -2.744277 1.017512 -0.949909
1 -2.779700 -0.741011 -1.091816
1 -0.662443 1.006823 1.598721
1 2.775630 1.244882 -0.553316
1 1.332289 -0.908651 -1.540997
1 1.107009 -0.965185 1.493915
1 -0.947250 -2.003837 -0.481906
1 1.095623 2.613578 0.350556
1 -1.186864 2.019811 0.276185

N-methylazepane
The number of imaginary frequencies: 0
B3LYP/6-31G**
Enthalpy = -330.534354811
6 -0.123724 -1.768559 0.779985
6 -0.123724 -1.768559 -0.779985
6 -0.850466 -0.578342 -1.425059
6 -0.850466 -0.578342 1.425059
6 -0.123724 0.768839 -1.236262
6 -0.123724 0.768839 1.236262
1 -0.594277 -2.690315 1.142355
1 -0.594277 -2.690315 -1.142355
1 -0.951502 -0.757329 -2.502387
1 -1.875529 -0.518078 1.044370
1 -0.851133 1.600321 -1.335329
1 0.595416 0.893796 2.055068
7 0.652331 0.862959 -0.000000
6 1.492463 2.051620 -0.000000
1 0.917986 2.998952 -0.000000
1 2.136105 2.052321 0.885746
1 2.136105 2.052321 -0.885746
1 -0.851133 1.600321 1.335329
1 -0.951502 -0.757329 2.502387
1 0.908855 -1.787038 1.144541
1 0.908855 -1.787038 -1.144541
1 -1.875529 -0.518078 -1.044370
1 0.595416 0.893796 -2.055068

N-methylazepane
The number of imaginary frequencies: 0
APFD/aug-cc-pVDZ
Enthalpy = -330.262189970
6 -0.124173 -1.749599 0.776635
6 -0.124173 -1.749599 -0.776635
6 -0.857783 -0.567553 -1.412621
6 -0.857783 -0.567553 1.412621
6 -0.124173 0.767764 -1.224833
6 -0.124173 0.767764 1.224833
1 -0.586118 -2.681431 1.144048
1 -0.586118 -2.681431 -1.144048
1 -0.976436 -0.744531 -2.494881
1 -1.884294 -0.506792 1.016680
1 -0.844754 1.615359 -1.311636
1 0.595917 0.894194 2.050876
7 0.653599 0.843264 -0.000000
6 1.503736 2.011210 -0.000000
1 0.935913 2.970142 -0.000000
1 2.151151 2.006138 0.890306
1 2.151151 2.006138 -0.890306
1 -0.844754 1.615359 1.311636
1 -0.976436 -0.744531 2.494881
1 0.914548 -1.756732 1.145893
1 0.914548 -1.756732 -1.145893
1 -1.884294 -0.506792 -1.016680
1 0.595917 0.894194 -2.050876

N-methylazepane
The number of imaginary frequencies: 0
APFD/aug-cc-pVTZ
Enthalpy = -330.355334937
6 -0.121742 -1.749206 0.775353
6 -0.121742 -1.749206 -0.775353
6 -0.853859 -0.569530 -1.410241
6 -0.853859 -0.569530 1.410241
6 -0.121742 0.763356 -1.224235
6 -0.121742 0.763356 1.224235
1 -0.578486 -2.673973 1.139279
1 -0.578486 -2.673973 -1.139279
1 -0.970148 -0.746248 -2.483503
1 -1.870596 -0.507585 1.015801
1 -0.838599 1.601933 -1.310440
1 0.593033 0.887046 2.043155
7 0.650728 0.846893 0.000000

6 1.490725 2.019665 0.000000
1 0.915859 2.963631 0.000000
1 2.132120 2.019859 0.883400
1 2.132120 2.019859 -0.883400
1 -0.838599 1.601933 1.310440
1 -0.970148 -0.746248 2.483503
1 0.909085 -1.753690 1.138273
1 0.909085 -1.753690 -1.138273
1 -1.870596 -0.507585 -1.015801
1 0.593033 0.887046 -2.043155

N-Methylpyrrole N-oxide
The number of imaginary frequencies: 0
B3LYP/6-31G**
Enthalpy= -324.573601410
6 1.618482 0.737646 0.097116
6 1.618414 -0.737501 0.098435
6 0.355178 -1.157788 -0.009827
6 0.355196 1.158013 -0.012549
1 2.501313 1.363007 0.116943
1 2.501241 -1.362844 0.118411
1 -0.094951 -2.133540 -0.104607
1 -0.095166 2.133563 -0.108432
7 -0.562707 -0.000157 -0.096040
8 -1.231366 -0.001924 -1.299503
6 -1.552719 0.001445 1.033634
1 -2.170095 0.888862 0.898347
1 -2.170107 -0.886355 0.900952
1 -1.049673 0.002897 2.005836

N-Methylpyrrole N-oxide
The number of imaginary frequencies: 0
APFD/aug-cc-pVDZ
Enthalpy= -324.322161700
6 1.610683 0.736027 0.095017
6 1.610665 -0.736086 0.095032
6 0.345214 -1.151806 -0.017876
6 0.345274 1.151814 -0.017496
1 2.496476 1.368927 0.116952
1 2.496422 -1.369018 0.117473
1 -0.112032 -2.131874 -0.113321
1 -0.111836 2.131884 -0.113512
7 -0.564700 0.000018 -0.102412
8 -1.230268 0.000086 -1.284835
6 -1.525150 -0.000050 1.037748
1 -2.149438 0.892362 0.915318
1 -2.149489 -0.892440 0.915342
1 -0.995171 -0.000046 2.002758

N-Methylpyrrole N-oxide
The number of imaginary frequencies: 0
APFD/aug-cc-pVTZ
Enthalpy= -324.417780070
6 1.602973 0.734263 0.088198
6 1.602915 -0.734116 0.089678
6 0.346661 -1.149278 -0.016996
6 0.346632 1.149492 -0.019776
1 2.481669 1.360937 0.117130
1 2.481615 -1.360728 0.119017
1 -0.106248 -2.121296 -0.109862
1 -0.106523 2.121310 -0.113620
7 -0.561759 -0.000171 -0.104665
8 -1.240514 -0.001994 -1.282137
6 -1.510277 0.001515 1.044399
1 -2.129362 0.887896 0.934686
1 -2.129527 -0.885055 0.937175
1 -0.968622 0.002830 1.992206

N-methyl-2-pyrroline N-oxide
The number of imaginary frequencies: 0
B3LYP/6-31G**
Enthalpy= -325.813525020
6 1.724798 0.621573 0.102158
6 1.525707 -0.878176 0.092168
6 0.240457 -1.196333 0.009033
6 0.351491 1.129373 -0.362271
1 1.988975 0.981248 1.105913
1 -0.268072 -2.148829 -0.029761
1 0.299008 1.241941 -1.443708
7 -0.647327 -0.014763 -0.108996
8 -1.525491 -0.166833 -1.127449
6 -1.401523 0.197798 1.174408
1 -2.060329 1.048181 0.998616
1 -2.003484 -0.695379 1.337294
1 -0.727303 0.375710 2.019018
1 2.339449 -1.592086 0.147047
1 2.526514 0.944681 -0.570564
1 -0.005125 2.037122 0.125734

N-methyl-2-pyrroline N-oxide
The number of imaginary frequencies: 0
APFD/aug-cc-pVDZ
Enthalpy= -325.559554428
6 1.699228 0.626505 0.099662
6 1.519410 -0.869653 0.081635
6 0.234035 -1.192443 -0.005468
6 0.338910 1.105798 -0.400816
1 1.929546 0.986067 1.119459
1 -0.279209 -2.150390 -0.043246
1 0.302095 1.165155 -1.494631
7 -0.648950 -0.016217 -0.112443
8 -1.552804 -0.168406 -1.083635

6 -1.335260 0.208120 1.193122
1 -2.009696 1.058643 1.037583
1 -1.929268 -0.689577 1.398113
1 -0.614306 0.399022 2.003893
1 2.341219 -1.584606 0.139856
1 2.522242 0.966507 -0.548808
1 -0.035484 2.039994 0.035154

N-methyl-2-pyrroline N-oxide
The number of imaginary frequencies: 0
APFD/aug-cc-pVTZ
Enthalpy= -325.657265137
6 1.700621 0.619649 0.098995
6 1.511624 -0.871104 0.073950
6 0.234307 -1.188048 -0.011483
6 0.337682 1.114584 -0.374683
1 1.948166 0.967604 1.108245
1 -0.276017 -2.136720 -0.053793
1 0.296959 1.220866 -1.455370
7 -0.644978 -0.014008 -0.111152
8 -1.543340 -0.154901 -1.096392
6 -1.341990 0.189963 1.188782
1 -2.008656 1.037663 1.049725
1 -1.929672 -0.704090 1.379165
1 -0.626813 0.366110 1.995222
1 2.323617 -1.583048 0.131502
1 2.506973 0.954709 -0.557000
1 -0.026455 2.023903 0.098124

N-methyl-3-pyrroline N-oxide
The number of imaginary frequencies: 0
B3LYP/6-31G**
Enthalpy= -325.814117087
6 1.659112 0.667757 0.078666
6 1.659089 -0.667824 0.078611
6 0.306599 -1.205279 -0.282567
6 0.306694 1.205298 -0.282477
1 0.205106 -1.468262 -1.338934
1 0.204970 1.468429 -1.338757
7 -0.643806 0.000008 -0.112990
8 -1.660544 0.000082 -0.983480
6 -1.186601 -0.000046 1.292211
1 -1.811178 0.888082 1.377663
1 -1.811409 -0.888032 1.377394
1 -0.387746 -0.000248 2.040368
1 2.513539 -1.301454 0.291465
1 2.513650 1.301364 0.291211
1 -0.052479 2.039171 0.326025
1 -0.052812 -2.039200 0.325669

N-methyl-3-pyrroline N-oxide
The number of imaginary frequencies: 0
APFD/aug-cc-pVDZ
Enthalpy= -325.560494446
6 1.645691 0.668572 0.073567
6 1.645454 -0.668675 0.073390
6 0.296916 -1.192751 -0.296104
6 0.297074 1.193022 -0.295011
1 0.199026 -1.441229 -1.363506
1 0.199004 1.442315 -1.362187
7 -0.643142 0.000052 -0.113380
8 -1.670807 0.000594 -0.948348
6 -1.141950 -0.000658 1.295399
1 -1.768933 0.891751 1.398233
1 -1.769241 -0.892986 1.397129
1 -0.315842 -0.001240 2.023173
1 2.503806 -1.307034 0.291256
1 2.504150 1.306504 0.292150
1 -0.071332 2.040938 0.299241
1 -0.071305 -2.041200 0.297504

N-methyl-3-pyrroline N-oxide
The number of imaginary frequencies: 0
APFD/aug-cc-pVTZ
Enthalpy= -325.657786634
6 1.645070 0.663747 0.071136
6 1.644692 -0.663946 0.071139
6 0.296218 -1.193971 -0.276184
6 0.296470 1.194302 -0.274766
1 0.197894 -1.476386 -1.325430
1 0.197546 1.478063 -1.323537
7 -0.637502 0.000082 -0.112470
8 -1.651287 0.000779 -0.974962
6 -1.162751 -0.000816 1.282626
1 -1.783542 0.885363 1.380999
1 -1.784105 -0.886783 1.379457
1 -0.349976 -0.001704 2.011704
1 2.500305 -1.297236 0.266788
1 2.500777 1.296522 0.267837
1 -0.061962 2.016985 0.344062
1 -0.062319 -2.017520 0.341402

N-methylpyrrolidine N-oxide
The number of imaginary frequencies: 0
B3LYP/6-31G**
Enthalpy= -327.050367463
6 1.603663 0.781996 0.090072
6 1.603543 -0.782088 0.090005
6 0.207002 -1.179916 -0.397304
6 0.206897 1.180170 -0.396436
1 1.804230 1.168334 1.093772
1 1.803291 -1.168510 1.093835
1 0.140440 -1.273784 -1.482064

1 0.140075 1.275167 -1.481082
7 -0.712799 0.000065 -0.102554
8 -1.821085 0.000278 -0.863836
6 -1.111849 -0.000465 1.347292
1 -1.725599 0.887101 1.494613
1 -1.724655 -0.888716 1.494402
1 -0.251770 -0.000117 2.025907
1 -0.218069 -2.074408 0.059459
1 2.376478 -1.189930 -0.566460
1 2.376240 1.189714 -0.566904
1 -0.217920 2.074288 0.061322

N-methylpyrrolidine N-oxide
The number of imaginary frequencies: 0
APFD/aug-cc-pVDZ
Enthalpy= -326.793508853
6 1.589304 0.777796 0.086399
6 1.589429 -0.777691 0.086687
6 0.199576 -1.167377 -0.402643
6 0.199576 1.167034 -0.403601
1 1.787755 1.170543 1.095653
1 1.788462 -1.170027 1.095978
1 0.130996 -1.252875 -1.494831
1 0.131267 1.251498 -1.495891
7 -0.711118 -0.000052 -0.102188
8 -1.817101 -0.000383 -0.838490
6 -1.078865 0.000575 1.343453
1 -1.693654 0.893068 1.502963
1 -1.693458 -0.891899 1.503806
1 -0.199700 0.000991 2.007486
1 -0.233713 -2.070548 0.044126
1 2.368126 -1.192045 -0.570014
1 2.368265 1.192085 -0.570035
1 -0.233833 2.070615 0.042220

N-methylpyrrolidine N-oxide
The number of imaginary frequencies: 0
APFD/aug-cc-pVTZ
Enthalpy= -326.893346480
6 1.583689 0.776507 0.082222
6 1.583959 -0.776276 0.082592
6 0.195638 -1.166252 -0.404176
6 0.195629 1.165640 -0.405808
1 1.779868 1.164568 1.083134
1 1.781225 -1.163802 1.083488
1 0.131965 -1.257010 -1.487102
1 0.132421 1.254540 -1.488921
7 -0.707597 -0.000090 -0.099394
8 -1.821665 -0.000610 -0.832887
6 -1.063806 0.000937 1.345564
1 -1.668754 0.887275 1.514681
1 -1.668737 -0.885172 1.515927
1 -0.180650 0.001392 1.988105
1 -0.230929 -2.060249 0.045229
1 2.355238 -1.185419 -0.570063
1 2.355403 1.185689 -0.569891
1 -0.231199 2.060364 0.041897

N-Methylpyrrole
The number of imaginary frequencies: 0
B3LYP/6-31G**
Enthalpy= -249.488688331
6 1.491542 0.711677 0.016064
6 1.491605 -0.711621 0.016055
6 0.175200 -1.119627 -0.014329
6 0.175126 1.119618 -0.014423
1 2.354859 1.362200 0.022539
1 2.354977 -1.362074 0.022380
1 -0.261470 -2.107877 -0.023476
1 -0.261582 2.107854 -0.023313
7 -0.625375 -0.000019 -0.039882
6 -2.073948 -0.000003 0.026688
1 -2.465245 0.883791 -0.482987
1 -2.464783 -0.886868 -0.477929
1 -2.436274 0.002840 1.061627

N-Methylpyrrole
The number of imaginary frequencies: 0
APFD/aug-cc-pVDZ
Enthalpy= -249.288183211
6 1.489036 0.710705 0.015404
6 1.488925 -0.710786 0.015503
6 0.170288 -1.116176 -0.014162
6 0.170438 1.116181 -0.014125
1 2.356556 1.366817 0.022926
1 2.356410 -1.366932 0.022099
1 -0.271895 -2.109242 -0.022631
1 -0.271591 2.109316 -0.023091
7 -0.623432 0.000002 -0.038810
6 -2.062873 0.000080 0.026085
1 -2.457582 0.887737 -0.486729
1 -2.457117 -0.891483 -0.480206
1 -2.425637 0.003751 1.067072

N-Methylpyrrole
The number of imaginary frequencies: 0
APFD/aug-cc-pVTZ
Enthalpy= -249.356058342
6 1.482743 0.707757 0.014014
6 1.482795 -0.707706 0.014044
6 0.170933 -1.111677 -0.012696
6 0.170896 1.111651 -0.012853
1 2.343550 1.357411 0.020707
1 2.343654 -1.357296 0.020302

1 -0.266361 -2.096940 -0.021290
1 -0.266420 2.096907 -0.021004
7 -0.620071 -0.000019 -0.035081
6 -2.057917 0.000016 0.023689
1 -2.446181 0.881288 -0.485809
1 -2.445715 -0.884811 -0.479914
1 -2.418727 0.003332 1.055381

N-methyl-2-pyrroline
The number of imaginary frequencies: 0
B3LYP/6-31G**
Enthalpy= -250.680844113
6 -1.580164 -0.617095 -0.028381
6 -1.374505 0.875399 0.139141
6 -0.078183 1.152311 -0.057952
6 -0.126568 -1.141239 0.048724
1 -2.221183 -1.073692 0.733501
1 0.400225 2.125893 -0.075806
1 0.069547 -1.998032 -0.603363
7 0.703034 0.017737 -0.334592
6 2.082715 -0.009804 0.113135
1 2.605033 -0.853831 -0.348712
1 2.590996 0.907491 -0.198498
1 2.181199 -0.106315 1.209794
1 -2.167178 1.601922 0.257257
1 -2.031563 -0.845992 -1.005419
1 0.111921 -1.439029 1.085388

N-methyl-2-pyrroline
The number of imaginary frequencies: 0
APFD/aug-cc-pVDZ
Enthalpy= -250.474897760
6 -1.568707 -0.618424 -0.041824
6 -1.373422 0.867641 0.143669
6 -0.075428 1.146756 -0.055101
6 -0.122719 -1.129168 0.063129
1 -2.232117 -1.089974 0.700208
1 0.406399 2.126023 -0.067721
1 0.090651 -2.007614 -0.564609
7 0.697385 0.016705 -0.335908
6 2.068680 -0.009515 0.106068
1 2.586282 -0.873344 -0.338055
1 2.588705 0.899868 -0.229335
1 2.166937 -0.082383 1.211143
1 -2.171183 1.598095 0.266286
1 -1.996673 -0.835249 -1.040073
1 0.108883 -1.396092 1.117864

N-methyl-2-pyrroline
The number of imaginary frequencies: 0
APFD/aug-cc-pVTZ
Enthalpy= -250.545365644
6 -1.566783 -0.615622 -0.040830
6 -1.366675 0.867463 0.140258
6 -0.075913 1.142950 -0.054270
6 -0.124034 -1.129298 0.062163
1 -2.223076 -1.076798 0.699968
1 0.401825 2.114080 -0.065610
1 0.085885 -1.996953 -0.565256
7 0.694942 0.016811 -0.330911
6 2.066120 -0.009751 0.104384
1 2.579776 -0.862289 -0.343329
1 2.577202 0.896919 -0.221821
1 2.162310 -0.089243 1.199546
1 -2.156465 1.592280 0.264383
1 -1.994180 -0.832908 -1.028568
1 0.105839 -1.397218 1.106833

N-methyl-3-pyrroline
The number of imaginary frequencies: 0
B3LYP/6-31G**
Enthalpy= -250.672907553
6 1.528447 -0.666301 -0.042760
6 1.528344 0.666445 -0.042837
6 0.117095 1.191194 0.005632
6 0.117228 -1.191234 0.005572
1 -0.027772 1.988358 0.749633
1 -0.027693 -1.988552 0.749369
7 -0.668125 -0.000121 0.352374
6 -2.037816 -0.000011 -0.113710
1 -2.562067 -0.882936 0.267128
1 -2.561699 0.883243 0.266871
1 -2.131388 -0.000136 -1.218156
1 2.398683 1.311000 -0.099085
1 2.398909 -1.310726 -0.098700
1 -0.164736 -1.623648 -0.977650
1 -0.165147 1.623686 -0.977416

N-methyl-3-pyrroline
The number of imaginary frequencies: 0
APFD/aug-cc-pVDZ
Enthalpy= -250.466571606
6 1.521902 -0.667274 -0.035307
6 1.521939 0.667277 -0.035246
6 0.112892 1.181910 -0.002914
6 0.112879 -1.181905 -0.002892
1 -0.043965 1.994056 0.731027
1 -0.043930 -1.994057 0.731061
7 -0.661297 -0.000001 0.350387
6 -2.024341 0.000009 -0.104413
1 -2.550089 -0.887833 0.280100

1 -2.550051 0.887912 0.280008
1 -2.122706 -0.000046 -1.215231
1 2.398626 1.315138 -0.086399
1 2.398551 -1.315206 -0.086377
1 -0.164501 -1.601313 -1.001119
1 -0.164484 1.601249 -1.001150

N-methyl-3-pyrroline
The number of imaginary frequencies: 0
APFD/aug-cc-pVTZ
Enthalpy= -250.536929435
6 1.518221 -0.662893 -0.036200
6 1.518247 0.662871 -0.036217
6 0.114080 1.180486 -0.001852
6 0.114036 -1.180456 -0.001870
1 -0.039768 1.983059 0.728936
1 -0.039806 -1.983093 0.728869
7 -0.660293 -0.000017 0.348406
6 -2.022204 0.000017 -0.104755
1 -2.543078 -0.881206 0.275184
1 -2.543166 0.881056 0.275492
1 -2.113460 0.000194 -1.206318
1 2.388019 1.304497 -0.087063
1 2.387994 -1.304525 -0.086916
1 -0.164467 -1.595390 -0.990852
1 -0.164497 1.595380 -0.990808

N-methylpyrrolidine
The number of imaginary frequencies: 0
B3LYP/6-31G**
Enthalpy= -251.909652936
6 1.497577 -0.778513 -0.057205
6 1.497372 0.778557 -0.058524
6 0.020846 1.157260 0.162650
6 0.020546 -1.157422 0.161652
1 1.855186 -1.162445 -1.016488
1 1.852351 1.160794 -1.019470
1 -0.174773 1.320605 1.242580
1 -0.176284 -1.322363 1.241118
7 -0.713585 0.000201 -0.346134
6 -2.126797 0.000069 -0.023954
1 -2.324282 -0.001009 1.066758
1 -2.607763 0.885506 -0.452595
1 -2.607865 -0.884463 -0.454333
1 -0.279500 2.069013 -0.365571
1 2.140735 1.200005 0.718830
1 2.139154 -1.198258 0.722541
1 -0.279129 -2.068498 -0.368147

N-methylpyrrolidine
The number of imaginary frequencies: 0
APFD/aug-cc-pVDZ
Enthalpy= -251.695112499

6 1.244938 -0.772727 -0.398674
6 1.244436 0.773124 -0.398851
6 0.070332 1.139457 0.542006
6 0.070461 -1.139704 0.541547
1 1.078591 -1.168279 -1.412898
1 1.076987 1.168300 -1.413042
1 0.444189 1.283586 1.570522
1 0.443966 -1.284497 1.570097
7 -0.842947 -0.000173 0.548978
6 -1.734764 -0.000001 -0.598183
1 -2.384292 -0.887405 -0.557362
1 -2.384301 0.887383 -0.557085
1 -1.224334 0.000150 -1.586110
1 -0.457805 2.060597 0.254965
1 2.196103 1.199785 -0.046899
1 2.196639 -1.198606 -0.045870
1 -0.457536 -2.060693 0.253766

N-methylpyrrolidine
The number of imaginary frequencies: 0
APFD/aug-cc-pVTZ
Enthalpy= -251.767456643
6 1.242315 -0.771295 -0.397569
6 1.241516 0.771955 -0.397780
6 0.069801 1.139314 0.541076
6 0.070235 -1.139596 0.540488
1 1.077140 -1.162833 -1.403038
1 1.074833 1.162985 -1.403207
1 0.440998 1.284660 1.560273
1 0.441155 -1.285562 1.559710
7 -0.839682 -0.000299 0.547297
6 -1.732126 -0.000122 -0.597051
1 -2.374768 -0.881005 -0.556023
1 -2.374482 0.880962 -0.555981
1 -1.220270 -0.000179 -1.573315
1 -0.454804 2.050376 0.250511
1 2.185336 1.192933 -0.047769
1 2.186243 -1.191140 -0.046466
1 -0.454047 -2.050634 0.249240

7-methyl-7-azanorcaradiene
The number of imaginary frequencies: 0
B3LYP/6-31G**
Enthalpy=-326.847852849
6 -0.436335 -0.772082 0.538090
6 -0.436471 0.772316 0.537614
1 0.915223 -2.506512 0.354302
6 0.825961 -1.437596 0.179389

6 0.826126 1.437617 0.179439
6 1.866633 0.726845 -0.309153
6 1.866540 -0.726960 -0.309293
1 0.915317 2.506552 0.354254
1 2.781998 1.236432 -0.598102
1 2.781823 -1.236652 -0.598315
7 -1.129485 -0.000070 -0.496887
6 -2.583903 -0.000008 -0.389471
1 -2.978859 0.887802 -0.893875
1 -2.978971 -0.887530 -0.894316
1 -2.957761 -0.000254 0.649659
1 -1.071784 -1.269090 1.272531
1 -1.071898 1.268947 1.272386

7-methyl-7-azanorcaradiene
The number of imaginary frequencies: 0
APFD/aug-cc-pVDZ
Enthalpy=-326.587401162
6 -0.440908 -0.771702 0.540513
6 -0.441103 0.771773 0.540032
1 0.915933 -2.507708 0.378060
6 0.818491 -1.435448 0.187430
6 0.818462 1.435488 0.187555
6 1.854984 0.725602 -0.315009
6 1.854986 -0.725524 -0.315176
1 0.915700 2.507750 0.378255
1 2.774525 1.239771 -0.607674
1 2.774473 -1.239686 -0.608029
7 -1.113813 -0.000122 -0.493746
6 -2.558149 0.000035 -0.400311
1 -2.951795 0.891930 -0.911486
1 -2.951887 -0.892435 -0.910438
1 -2.943607 0.000631 0.641665
1 -1.088457 -1.270433 1.272807
1 -1.088778 1.269698 1.272864

7-methyl-7-azanorcaradiene
The number of imaginary frequencies: 0
APFD/aug-cc-pVTZ
Enthalpy=-326.673466629
6 -0.436903 -0.768745 0.535988
6 -0.437204 0.768606 0.535592
1 0.913056 -2.492779 0.369844
6 0.818727 -1.428362 0.184642
6 0.818439 1.428441 0.184832
6 1.849643 0.723140 -0.312072
6 1.849784 -0.722830 -0.312262
1 0.912374 2.492856 0.370199
1 2.762042 1.233209 -0.600944
1 2.762210 -1.232713 -0.601385
7 -1.114056 -0.000207 -0.491731
6 -2.556809 0.000055 -0.393940
1 -2.948535 0.885330 -0.898164
1 -2.948769 -0.885248 -0.897951
1 -2.928430 0.000200 0.642626
1 -1.079516 -1.263954 1.260498
1 -1.080095 1.262722 1.260705

7-methyl-7-azanorcaradiene N-oxide
The number of imaginary frequencies: 0
B3LYP/6-31G**
Enthalpy=-401.989673111
6 0.254634 -0.764735 -0.841923
6 0.252136 0.771185 -0.840037
1 -1.028447 -2.509205 -0.492686
6 -0.945139 -1.435976 -0.356914
6 -0.946719 1.438057 -0.350508
6 -1.905222 0.725971 0.283316
6 -1.905061 -0.727865 0.278690
1 -1.030156 2.512205 -0.478847
1 -2.764047 1.235150 0.711207
1 -2.764700 -1.239780 0.701733
7 1.068366 -0.002117 0.237861
6 2.529856 0.001846 0.018614
1 2.925968 0.891977 0.509904
1 2.929522 -0.891087 0.502142
1 2.778608 0.006678 -1.046313
1 0.878737 -1.280348 -1.566656
1 0.877476 1.288921 -1.561995
8 0.713946 -0.006324 1.488632

7-methyl-7-azanorcaradiene N-oxide
The number of imaginary frequencies: 0
APFD/aug-cc-pVDZ
Enthalpy=-401.683840334
6 0.267783 -0.765912 -0.850829
6 0.266149 0.768828 -0.851379
1 -1.024147 -2.511770 -0.522626
6 -0.930445 -1.434678 -0.374622
6 -0.930346 1.435990 -0.371472
6 -1.873434 0.725588 0.290486
6 -1.874281 -0.725756 0.287379
1 -1.023187 2.513828 -0.514689
1 -2.730252 1.239817 0.732943
1 -2.732553 -1.240742 0.726194
7 1.044658 -0.001765 0.246063
6 2.499037 0.001073 0.057002
1 2.886681 0.896917 0.558671
1 2.889451 -0.896205 0.554123
1 2.772801 0.004022 -1.007715
1 0.911605 -1.285576 -1.565411
1 0.911128 1.288788 -1.564505
8 0.659887 -0.003440 1.470147

7-methyl-7-azanorcaradiene N-oxide
The number of imaginary frequencies: 0
APFD/aug-cc-pVTZ
Enthalpy=-401.795253802
6 0.263928 -0.763667 -0.830803
6 0.262819 0.766694 -0.829889
1 -1.020975 -2.497238 -0.498128
6 -0.934220 -1.427234 -0.358865
6 -0.934720 1.428339 -0.355848
6 -1.888396 0.722350 0.276093
6 -1.888491 -0.722963 0.273851
1 -1.021181 2.498858 -0.491328
1 -2.745236 1.233196 0.700001
1 -2.745925 -1.234922 0.695253
7 1.051552 -0.001200 0.238865
6 2.499999 0.000973 0.024261
1 2.896081 0.890650 0.510218
1 2.898170 -0.888494 0.509050
1 2.746718 0.001794 -1.038041
1 0.897166 -1.279772 -1.542379
1 0.897013 1.283080 -1.540378
8 0.693974 -0.003214 1.478860

Benzene
The number of imaginary frequencies: 0
B3LYP/6-31G**
Enthalpy= -232.258203982
6 0.814686 -1.134019 -0.000280
6 -0.574873 -1.272443 0.000166
1 2.470537 0.246283 0.000304
6 1.389539 0.138440 0.000083
6 -1.389553 -0.138436 -0.000008
6 -0.814646 1.134003 -0.000131
6 0.574828 1.272409 0.000062
1 -2.470548 -0.246213 0.000090
1 -1.448104 2.016576 -0.000143
1 1.021853 2.262547 0.000193
1 1.448267 -2.016423 -0.000160
1 -1.021898 -2.262492 0.000362

Benzene
The number of imaginary frequencies: 0
APFD/aug-cc-pVDZ
Enthalpy= -232.065334419
6 1.311421 -0.479269 0.000555
6 0.240609 -1.375214 -0.000334
1 1.909110 1.597376 -0.000326
6 1.070691 0.896171 -0.000158
6 -1.070981 -0.895852 0.000030
6 -1.311271 0.479172 0.000275
6 -0.240382 1.375153 -0.000145
1 -1.908984 -1.597560 -0.000090
1 -2.337620 0.855130 0.000015
1 -0.429291 2.451781 -0.000324
1 2.337521 -0.855853 -0.000059
1 0.428743 -2.451840 -0.000558

Benzene
The number of imaginary frequencies: 0
APFD/aug-cc-pVTZ
Enthalpy= -232.124979909
6 0.746072 -1.171792 -0.000259
6 -0.641901 -1.231916 0.000215
1 2.471100 0.107159 0.000252
6 1.387945 0.060138 0.000090
6 -1.387961 -0.060107 -0.000025
6 -0.746027 1.171770 -0.000111
6 0.641863 1.231872 0.000077
1 -2.471107 -0.107112 -0.000005
1 -1.327926 2.086560 -0.000240
1 1.142586 2.193496 0.000142
1 1.328037 -2.086456 -0.000386
1 -1.142632 -2.193439 0.000322

Trimethylamine
The number of imaginary frequencies: 0
B3LYP/6-31G**
Enthalpy= -169.795990862
6 0.955144 -0.570336 0.000000
1 1.975859 -0.187524 0.000000
1 0.766308 -1.179993 0.889170
1 0.766308 -1.179993 -0.889170
7 -0.000000 0.590677 -0.000000
8 -1.154917 0.229348 -0.000000

Trimethylamine
The number of imaginary frequencies: 0
APFD/aug-cc-pVDZ
Enthalpy= -169.665311125
6 0.946529 -0.566157 0.000000
1 1.974762 -0.185648 0.000000
1 0.754389 -1.180394 0.893556
1 0.754389 -1.180394 -0.893556
7 -0.000000 0.584434 0.000000
8 -1.145339 0.231542 -0.000000

Trimethylamine
The number of imaginary frequencies: 0
APFD/aug-cc-pVTZ
Enthalpy= -169.716346849
6 0.945294 -0.566816 -0.000000
1 1.963677 -0.186875 -0.000000
1 0.755055 -1.174879 0.886696

1 0.755055 -1.174879 -0.886696
7 -0.000000 0.579570 0.000000
8 -1.143194 0.235067 0.000000

Propane
The number of imaginary frequencies: 0
Total Energy (B3LYP/6-31G*)= -119.14424274
ZPE= -119.040122
Enthalpy= -119.034676
Gibb’s Free Energy= -119.065764

C	1.27719	-0.25986	0.00002
C	0.	0.5865	0.
H	2.1765	0.36717	0.00003
H	1.32187	-0.90744	0.8847
H	1.3219	-0.90745	-0.88465
C	-1.27719	-0.25986	-0.00002
H	-0.00001	1.24736	0.8776
H	0.00001	1.24735	-0.87762
H	-2.1765	0.36717	-0.00004
H	-1.32187	-0.90745	-0.88469
H	-1.3219	-0.90744	0.88467

Propane
The number of imaginary frequencies: 0
Total Energy (B3LYP/6-31G**)= -119.15535976
ZPE= -119.051645
Enthalpy= -119.046182
Gibb’s Free Energy= -119.077296

C	1.27719	-0.25986	0.00002
C	0.	0.5865	0.
H	2.1765	0.36717	0.00003
H	1.32187	-0.90744	0.8847
H	1.3219	-0.90745	-0.88465
C	-1.27719	-0.25986	-0.00002
H	-0.00001	1.24736	0.8776
H	0.00001	1.24735	-0.87762
H	-2.1765	0.36717	-0.00004
H	-1.32187	-0.90745	-0.88469
H	-1.3219	-0.90744	0.88467

Propane
The number of imaginary frequencies: 0
Total Energy (M06/6-311G+(d,p))= -119.06693725
ZPE= -118.964632
Enthalpy= -118.959147
Gibb’s Free Energy= -118.990276

C	1.27719	-0.25986	0.00002
C	0.	0.5865	0.
H	2.1765	0.36717	0.00003
H	1.32187	-0.90744	0.8847
H	1.3219	-0.90745	-0.88465
C	-1.27719	-0.25986	-0.00002
H	-0.00001	1.24736	0.8776
H	0.00001	1.24735	-0.87762
H	-2.1765	0.36717	-0.00004
H	-1.32187	-0.90745	-0.88469
H	-1.3219	-0.90744	0.88467
